# High-pressure reactions between the pnictogens: the rediscovery of BiN

**DOI:** 10.3389/fchem.2023.1257942

**Published:** 2023-10-12

**Authors:** K. Glazyrin, A. Aslandukov, A. Aslandukova, T. Fedotenko, S. Khandarkhaeva, D. Laniel, M. Bykov, L. Dubrovinsky

**Affiliations:** ^1^ Deutsches Elektronen-Synchrotron DESY, Hamburg, Germany; ^2^ Material Physics and Technology at Extreme Conditions, Laboratory of Crystallography, University of Bayreuth, Bayreuth, Germany; ^3^ Bayerisches Geoinstitut, University of Bayreuth, Bayreuth, Germany; ^4^ Centre for Science at Extreme Conditions, School of Physics and Astronomy, University of Edinburgh, Edinburgh, United Kingdom; ^5^ Institute of Inorganic Chemistry, University of Cologne, Cologne, Germany

**Keywords:** pnictogens, binary nitrides, high-pressure chemistry, diamond anvil cell, single crystal, X-ray diffraction, high-pressure synthesis

## Abstract

We explore chemical reactions within pnictogens with an example of bismuth and nitrogen under extreme conditions. Understanding chemical reactions between Bi and N, elements representing the first and the last stable elements of the nitrogen group, and the physical properties of their compounds under ambient and high pressure is far from being complete. Here, we report the high-pressure high-temperature synthesis of orthorhombic *Pbcn* BiN (S.G. #60) from Bi and N_2_ precursors at pressures above 40 GPa. Using synchrotron single-crystal X-ray diffraction on the polycrystalline sample, we solved and refined the compound’s structure and studied its behavior and compressibility on decompression to ambient pressure. We confirm the stability of *Pbcn* BiN to pressures as low as 12.5(4) GPa. Below that pressure value, a group–subgroup phase transformation occurs, resulting in the formation of a non-centrosymmetric BiN solid with a space group *Pca*2_1_ (S.G. #29). We use *ab initio* calculations to characterize the polymorphs of BiN. They also provide support and explanation for our experimental observations, in particular those corresponding to peculiar Bi–N bond evolution under pressure, resulting in a change in the coordination numbers of Bi and N as a function of pressure within the explored stability field of *Pbcn* BiN.

## 1 Introduction

Theoretical and experimental studies under extreme conditions have recently rejuvenated the interest in the chemistry of pnictogens and their compounds (Young et al., 2006; Niwa et al., 2014; Wang et al., 2016; Laniel et al., 2018; Bykov et al., 2020; Laniel et al., 2022a; Laniel et al., 2022b; Aslandukov et al., 2022; Zhang et al., 2022). Each of the elements belonging to the nitrogen group is fascinating in its pure form; however, their compounds are equally important either for our everyday life or industry, and consequently, it results in an active multi-billion market either for raw and purified materials or compounds and derivatives.

Unlike the unhealthier high-Z counterparts of the pnictogen group (e.g., As and Sb), Bi has very low toxicity for a heavy Z element. It reacts with oxygen, sulfur, and halogens, but at the same time, it is somewhat reluctant to form stable compounds with carbon (H. G. N., 1896) and nitrogen at ambient pressure (Franklin, 1905). More than a century ago, Franklin was the first to synthesize the binary BiN, but its spontaneous decomposition in air and reaction with water prevented its further investigation. We know that BiN, similar to HgN, can be used as a nitridizing agent (Schurman and Fernblius, 1930); however, until now, the crystal structure has not been reported.

In contrast to Franklin, who first synthesized BiN through a reaction between BiBr_3_ and NH_3_ at ambient pressure, here, we report the synthesis of the BiN compound, obtained by compressing the Bi and N_2_ precursors to high-pressure conditions and laser heating. In the following paragraphs, we present a description of our experiments up to 50 GPa and discuss the experimentally determined structure of BiN, its striking evolution under stress and decompression. At last, but not the least, we compare our experimental observations with the results of our *ab initio* calculations.

Being discovered more than a century ago, BiN has been a challenge for chemists and material scientists. From Cheetham et al. (2022), we know that synthesis discovery may precede functional implementation by many decades. We hope that the study presented in the subsequent sections may not only fill blanks in our understanding of pnictogen and metal–nitrogen chemistry but lay the ground work for future practical applications.

## 2 Materials and methods

All high-pressure experiments conducted during these studies were prepared and conducted at the P02.2 beamline of PETRA III, DESY (Liermann et al., 2015). Small traces of Bi powder (Sigma-Aldrich 264008, ≥99.99% trace metals basis) were loaded into the sample chambers of several diamond anvil cells (DACs) equipped with diamond anvils having a culet diameter of 300 μm. Sample chambers of 150 μm in diameter were produced by drilling holes using an electrical discharge machine in rhenium gaskets indented to a thickness of 40–50 μm (initial thickness of 250 μm). The sample size varied between loadings but never exceeded 60∙60∙3 μm^3^. Molecular nitrogen gas was loaded at pressures of 1.4–1.5 kbar into the sample chamber using the P02.2 beamline’s high-pressure gas loader (Liermann et al., 2015). For the synthesis of BiN, we used either the pulse or the continuous wave mode of a near-infrared (NIR) laser described by Konôpková et al. (2021). Bismuth, a metallic element, was used as the NIR laser absorber. We used screw-driven symmetric diamond anvil cells (DACs), and the sample pressure was determined using the Raman signal of diamond measured at its tip (Akahama and Kawamura, 2006). During decompression below 10 GPa, where the signal from the diamond Raman edge becomes unreliable, we used the textbook reference of N_2_ Raman shift (2,330 cm^−1^) to confirm full quenching to ambient conditions. The temperatures achieved by laser heating were measured by means of spectral radiometry (Konôpková et al., 2021).

X-ray diffraction patterns were recorded using the Perkin Elmer XRD1621 detector of P02.2 with the sample illuminated by an incident X-ray beam with a wavelength of either 0.4839, 0.2904, or 0.2909 Å (depending on the experiment) focused down to either 0.9∙0.9 or 2∙2 μm^2^ spot (horizontal∙vertical, full width at half maximum) by means of compound refractive lenses (CRLs, for 0.4839 Å) or Kirkpatrick–Baez mirrors (KBs, for 0.2904 or 0.2909 Å). Each synthesis attempt was followed by a 2D mapping of the heated area, revealing the most promising sample positions for a subsequent multi-grain single-crystal data collection. The latter data were acquired with a step of 0.5° during a continuous rotation of ±30° along the ω rotation axis.

The following software applications were used for the data analysis, structure solution, refinement, and visualization: DIOPTAS (Prescher and Prakapenka, 2015), CrysAlis^PRO^ (Rigaku Crysalis, 2020), XDI (Hrubiak et al., 2019), DAFi by Aslandukov et al. (2022), JANA 2006 (Petricek et al., 2006), Olex2 (Dolomanov et al., 2009), SHELX (Muller, 2006; Sheldrick, 2008), VESTA (Momma and Izumi, 2011), EosFit (Gonzalez-Platas et al., 2016), and CrystalMaker X (Palmer, 2022). The resulting CIF files describing parameters of single-crystal data solution and refinement are available via the Cambridge Crystallographic Data Centre deposition (CCDC, 2023).

First-principles calculations were performed using the framework of density functional theory (DFT), as implemented in the Vienna Ab initio Simulation Package (VASP) (Kresse and Furthmüller, 1996). The projector augmented-wave (PAW) method (Blochl, 1994; Kresse and Joubert, 1999) was used to expand the electronic wave function in plane waves. The calculations were carried out using two approaches: (A1) one neglecting the contribution of the van der Waals (VdW) forces and (A2) one taking them into account. For the former, the exchange-correlation functional described by the Perdew–Burke–Ernzerhof (PBE) formulation (Perdew et al., 1996) under the generalized gradient approximation (GGA) was used. In the second approach, van der Waals forces were captured employing the VdW-optB88 functional (Klimeš et al., 2010; Klimeš et al., 2011). The “*Bi_d*” (ENMAX = 242.839 eV) and “*N*” (ENMAX = 400 eV) PAW potentials with valence configurations of 6*s* ^2^5*d* ^10^6*p* ^3^ for Bi and 2*s* ^2^2*p* ^3^ for N were used. For the sake of clarity, here and in the following paragraphs, when necessary, we will use the superscript VdW, indicating results obtained by (A2). We employed the Monkhorst–Pack scheme with 8 × 4×4 for *Pbcn-*BiN or 4 × 8×4 for *Pca*2_1_ *-*BiN *k*-points for Brillouin zone sampling, and the plane–wave kinetic energy cutoff was set to 800 eV, ensuring total energy convergence to better than 0.5 meV/atom. For electron band structure calculations, the two-fold denser *k*-point grids were used. The finite displacement method, as implemented in Phonopy (Togo and Tanaka, 2015), was used to calculate phonon frequencies and phonon band structures. The 2 × 2 × 2 supercells with 4 × 2 × 2 and 2 × 4 × 2 *k*-point grids for *Pbcn-*BiN and *Pca*2_1_ *-*BiN, respectively, were used for phonon calculations, and the displacement amplitude was 0.01 Å.

## 3 Results and discussion

### 3.1 Phase stability field of BiN below 50 GPa

Attempts to obtain a chemical reaction between pure Bi and N_2_ were made to pressures of up to 50 GPa through sample laser heating. The first synthesis attempts at pressures of 12–15 GPa and ∼30 GPa were unsuccessful. Indeed, although at these pressure ranges, laser heating to temperatures of ∼1800 (200) K resulted in bismuth melting, the collected X-ray diffraction data did not reveal any signs of a new phase being produced. However, upon further increasing the pressure to 42.5 (3) GPa and laser heating, new diffraction lines, suggestive of a chemical reaction and the formation of a novel compound, were observed. Although all synthesis attempts were conducted at high temperature, the data discussed here and in the following paragraphs were collected at room temperature. The analysis of the thereafter obtained single-crystal X-ray diffraction data enabled to unambiguously index the produced solid to have lattice parameters of *a* = 4.8941(5) Å, *b* = 9.4020(10) Å, and *c* = 9.176(4) Å, and the *Pbcn* space group (S.G. #60). The *R*-factors of the best solution are as follows: *R*_*int*_ *=* 2.5% and *R*_*1*_ = 2.6% for *I > 2σ(I)*, with *I* and *σ* being the integrated intensity and its standard deviation value, respectively. More information on the single-crystal data, along with the tables containing the crystallographic parameters for this and other pressure points, is shown in Supplementary Material.

**FIGURE 1:**
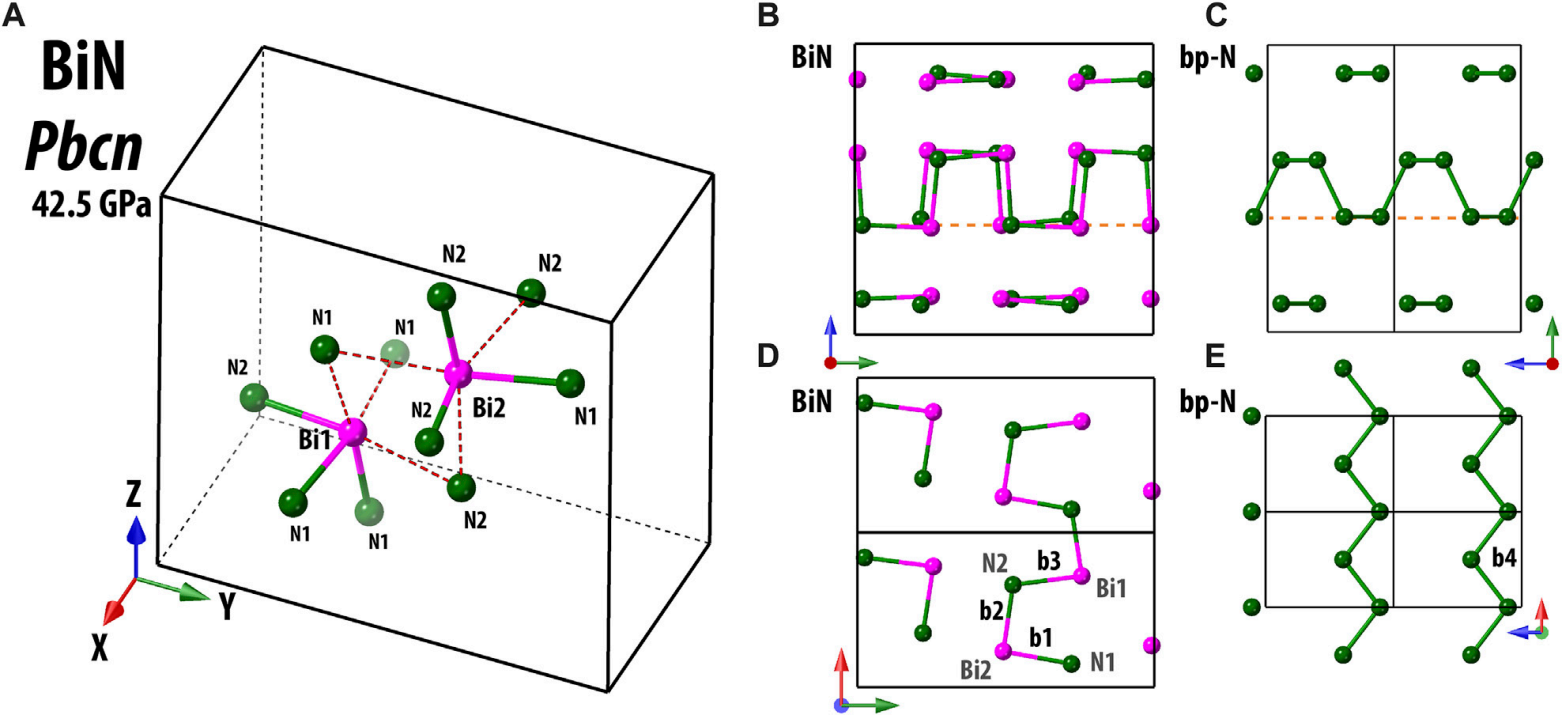
Structure of *Pbcn* BiN at 42.5(3) GPa and ambient temperature is shown in **(A)**. Here and in the following, the frame of the unit cell is indicated by solid and dashed black lines. Bi and N atoms are illustrated using pink and dark green spheres, respectively. The closest inter-atomic bonds are indicated by solid double color lines, and the red dashed lines are guides to the eye, underlining the octahedral shape of Bi sites. In panels **(B–E)**, we illustrate a comparison of the atomic arrangements between *Pbcn* BiN at 42.5(3) GPa and black phosphorus-type polymeric nitrogen (bp-N) at 140 GPa (Laniel et al., 2020). Considering the projection shown in **(B, C)**, for both structures, we see a similar “arm-chair” pattern, a bit more irregular for the case of BiN. However, if we take a closer look at the contents of a shallow atomic layer indicated by dashed orange lines, then, the arrangement is different, namely, the “arm-chair” in **(D)** and a “zig-zag” in **(E)**, respectively. Using the labels of b1, b2, b3, and b4, we indicate some characteristic interatomic bonds. b1, b2, and b3 belong to Bi–N and have values of 2.2028(10), 2.1282(3), and 2.1987(3) Å, respectively. The reported value of bp-N b4 is 1.345 (6) Å.

The structure of *Pbcn* BiN is constituted of four crystallographically independent atoms, two Bi atoms (Bi1 and Bi2) and two N atoms (N1 and N2) (Figure 1). The Bi1, Bi2, N1, and N2 atoms occupy Wyckoff site 8*d*, and the number of formula units per unit cell is *Z* = 16. The structure of the compound can be visualized as consisting of edge-sharing octahedra that are distorted to accommodate the Bi–N bonding characteristics.

Considering that N and Bi have similar electronic configurations of the outer electronic shells, it would be natural to draw a comparison of BiN with other pnictogen phases, including the black phosphorous-type phase of nitrogen (*Cmce, bp*-N) (Laniel et al., 2020), where each N has a direct bond with three other neighbors. As for *Pbcn* BiN, we can easily imagine that Bi is also bound to three closest neighbors. Then, the structure can be described as polymeric, with weakly interconnected Bi–N layers with an “arm-chair” arrangement along the *y*-axis of the unit cell. A similar, but more regular, arrangement is seen in *bp*-N. Nevertheless, within the specified individual layers, we observe a pronounced distinctiveness, as illustrated in Figures 1D, E.

We can also compare *Pbcn* BiN with cubic gauche polymeric nitrogen (*I*2_1_3*,* S.G. #199, *cg*-N) (Eremets et al., 2004) and cubic single-bonded AsN (*P*2_1_3*,* S.G. #198) (Ceppatelli et al., 2022). The synthesized orthorhombic polymorph of BiN is of lower symmetry and features a fairly specific 3D arrangement. Although the local tetrahedral environment of nitrogen binding to the electrons of nearest As and Bi looks alike, the Bi–N–Bi angles are generally lower than As–N–As angles. From Cepatelli *et al.*, we know that at 38.6 GPa, the As–N–As angles spread from 103.4 (4)° to 112.6 (4)°. At the same time with *Pbcn* BiN, at a pressure of 42.5 (3) GPa, the Bi–N1–Bi angles have values of 98.8 (6)°, 105.1 (6)°, and 108.3 (4)°, while the values of Bi–N2–Bi angles are 94.2 (6)°, 102.4 (7)°, and 105.6 (4)°. Some of these angles are somewhat smaller than the reference angles of 108.8 (3)° in cg-N (115.4 GPa) (Eremets et al., 2004) and 105.1 (1)° of *bp*-N (140 GPa) (Laniel et al., 2020). This evidence emphasizes the importance of electronic density contrast between different elements in the nitrogen group, resulting in distinct crystal chemistry and in a strong influence on the properties of synthesized materials. The peculiarity of the *Pbcn* BiN structural framework is likely related to an increased electronic density featured by our electronic localization function calculations, which could be attributed to the nitrogen lone pair (Supplementary Figure S7).

**FIGURE 2:**
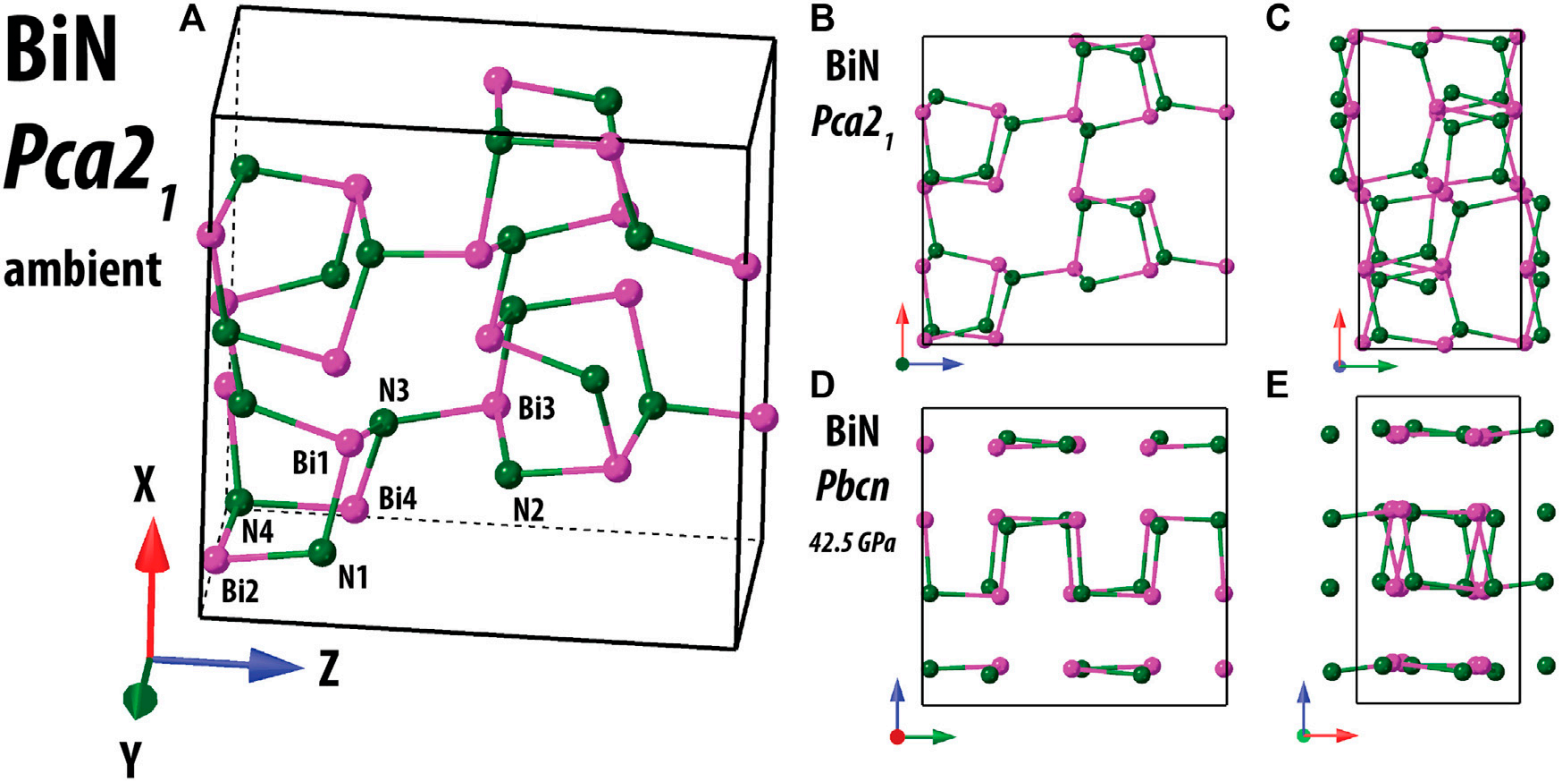
Structure of *Pca*2_1_ BiN at ambient conditions in comparison with *Pbcn* BiN measured at 42.5(3) GPa and at ambient temperature. To the left, in **(A)**, we show the 3D bonding network of the compound. Independent crystallographics sites are indicated. As expected, Bi is connected with 3 N if we consider the shortest Bi–N distances. In the panels **(B–E)**, we compare the similar projections for *Pca*2_1_ and *Pbcn* structures. It should be noted that *Pca*2_1_ can be converted into *Pbc*2_1_, which will have the same unit cell basis as *Pbcn*; however, we prefer to use the standard *Pca*2_1_ setting. Analysis of the projections allows us to confirm that the group–subgroup transformation *Pbcn→ Pca*2_1_ results in a strong distortion of the 3D bonding network.

Our observations indicate that in *Pbcn* BiN, Bi should have three single bonds with N (*sp* ^3^ hybridization), and as we will show in the upcoming section, this should hold true for Bi1 for *Pbcn* BiN below 50 GPa, but the situation for Bi2 requires an additional discussion and comparison with *ab initio* calculations. In addition to synthesizing *Pbcn* BiN at 42.5(3) GPa, we also successfully produced it at pressures of 47.4(2) GPa and 51(1) GPa. In all three syntheses, we could not find any other Bi–N compounds than *Pbcn* BiN. We also collected the data on decompression and report that upon quenching to 1 bar, *Pbcn* BiN undergoes a solid–solid phase transition toward a phase with the space group *Pca*2_1_, presumably via a group–subgroup transformation. It is interesting to note that similar behavior is observed for δ-P_3_N_5_ (Laniel et al., 2022a), spontaneously shifting into α′-P_3_N_5_ upon decompression below 7 GPa (*C*2/*c → P*2_1_/*c*). The crystallographic details of *Pca*2_1_ BiN are provided in the Supplementary Material. We verified the formation of *Pca*2_1_ in two separate different loadings: once by means of powder diffraction (the sample stabilized by an Ar atmosphere after being decompressed in a glovebox) and once by means of single-crystal diffraction and subsequent data analysis (the sample was carefully decompressed until the Raman shift of molecular nitrogen corresponded to ambient pressure). The *R*-factors of the best *Pca*2_1_ solution are as follows: *R*_*int*_ = 1.4% and *R*_*1*_ = 2.9% for *I > 2σ(I)*. Figure 2 shows the structures of *Pca*2_1_ and *Pbcn* BiN side-by-side.

Comparative analysis of *Pca*2_1_ and *Pbcn* BiN shows that a loss of a symmetry element on decompression (*Pbcn → Pca*2_1_ *= Pbc*2_1_) resulted in a significant structural reorganization. We obtain twice more independent Bi and N positions (Wyckoff sites 4*a*) without a change in formula units per unit cell (i.e., *Z* = 16). Considering the local nitrogen environment of *Pca*2_1_ BiN and the Bi–N–Bi angles, we see a relatively narrow distribution of their values around 111° (a value close to an optimal angle for *sp* ^*3*^ -hybridized N), with the smallest and the largest values being 100.9 (5)° and 114.6 (7)°, respectively. These values are in a closer agreement with the As–N–As angles reported for the cubic AsN (Ceppatelli et al., 2022).

Although our knowledge of pnictogen chemistry is still far from being complete, the comparison of the known structures reveals some interesting observations in relation to their crystal chemistry. As shown in the Supplementary Material, there is a direct group–subgroup path *Cmce→ Pbcn →Pca*2_1_ with the first space group being that adopted by *bp*-N and the others for Bi–N. If we compare AsN with *cg*-N, we also see that *P*2_1_3 is also a subgroup of *I*2_1_3. It is conceivable that several yet unexplored high-pressure phases of pnictogens with the 1:1 ratio may reside within the designated crystallographic group–subgroup space. Moreover, it is impossible to make a group–subgroup transformation from *I*2_1_3 *or P*2_1_3 to *Pca*2_1_, suggesting that binary BiN and AsN should belong to different classes, considering the crystal chemistry point of view.

**FIGURE 3:**
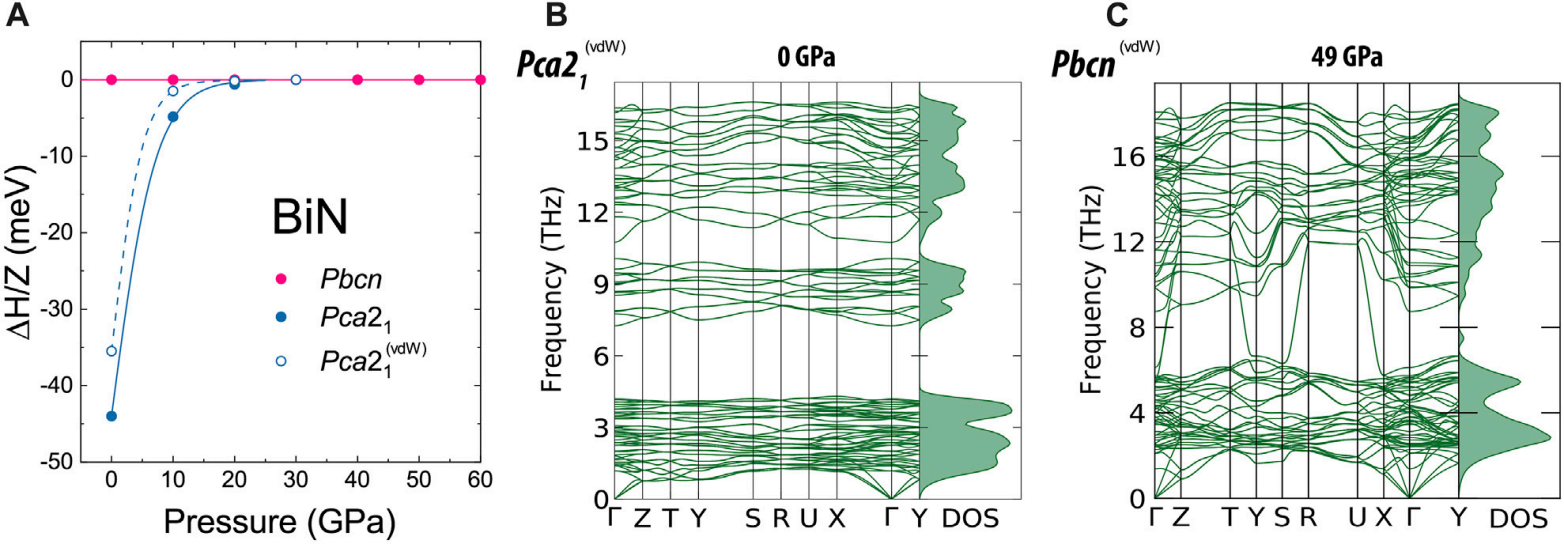
Results of *ab initio* calculations at 0 K with respect to the phase stability of *Pca*2_1_ and *Pbcn* phases of BiN. In **(A)**, we show the calculations of relative enthalpy between the two phases normalized to a molecular unit value. Considering both approximations, namely, with and without the involvement of the van der Waals forces, the enthalpy of *Pbcn* calculated in both approximations is taken as a reference. Both types of calculations predict the phase transition *Pbcn→ Pca*2_1_ in between 10 and 20 GPa. It is worth noting that the structure relaxation of *Pca*2_1_ above 20 GPa spontaneously transforms *Pca*2_1_ to *Pbcn*. The calculations involving van der Waals contribution have a better agreement with respect to the experimental volume of *Pca*2_1_. The calculations suggest that van der Waals forces play a bigger role for *Pca*2_1_ BiN and its stability field. Considering the calculations on *Pbcn*, we did not see a significant difference between calculations including van der Waals forces and those without them. Our phonon dispersion calculations support the stability of the phases, as calculated here for **(B)** *Pca*2_1_ at 1 bar and **(C)** *Pbcn* at 49 GPa. Additional results of phonon dispersion calculations for pressure points 12 and 30 GPa are shown in the Supplementary Material.

Concerning the stability of the individual *Pbcn* and *Pca*2_1_ BiN phases, we experimentally confirmed the presence of *Pbcn* at pressures as low as 12.5(4) GPa. Unfortunately, we do not have experimental datapoints between 12.5(4) GPa and ambient pressure. Here, we performed conventional *ab initio* calculations investigating the stability of the discovered BiN polymorphs with and without the contribution of van der Waals forces. It was indicated by Ceppatelli et al. (2022) that calculations of pnictogen materials with a larger number of electrons per atom are intrinsically hard and may not capture all the features of the materials at ambient temperature, and indeed, we will see some discrepancies in our discussion of BiN compressibility. At the same time, our *ab initio* calculations strongly support the experimental observations with respect to the phase stability; Figure 3 shows the plot of relative enthalpy between the compounds and the phonon dispersion plots calculated for *Pca*2_1_ at 1 bar and for *Pbcn* at 49 GPa. At the same time, the enthalpy calculations, shown in Figure 3, suggest the thermodynamic stability of the *Pca*2_1_ and *Pbcn* BiN polymorphs at lower and higher pressures, respectively (Figure 3A). The phonon calculations provide additional support for the polymorph dynamic stability (Figures 3B, C), and in the Supplementary Material, we show the results of our calculations, indicating the semiconducting nature of BiN with a band gap decreasing from 1.13 eV (*Pca*2_1_^VdW^) to 0.45 eV (*Pbcn* ^VdW^) if we consider 0 and 49 GPa, respectively. Both approximations employed in *ab initio* calculations confirmed the dynamical stability of the phases in their corresponding stability fields. The calculation including van der Waals forces provides better agreement with an experimental unit cell volume for *Pca*2_1_ BiN. We did not see a considerable difference between the results produced by different approximations for the *Pbcn* phase. In the Supplementary Material, we also demonstrate the results of the structure mechanical stability calculation additionally supporting our conclusion.

### 3.2 Peculiar compressibility of BiN

**FIGURE 4:**
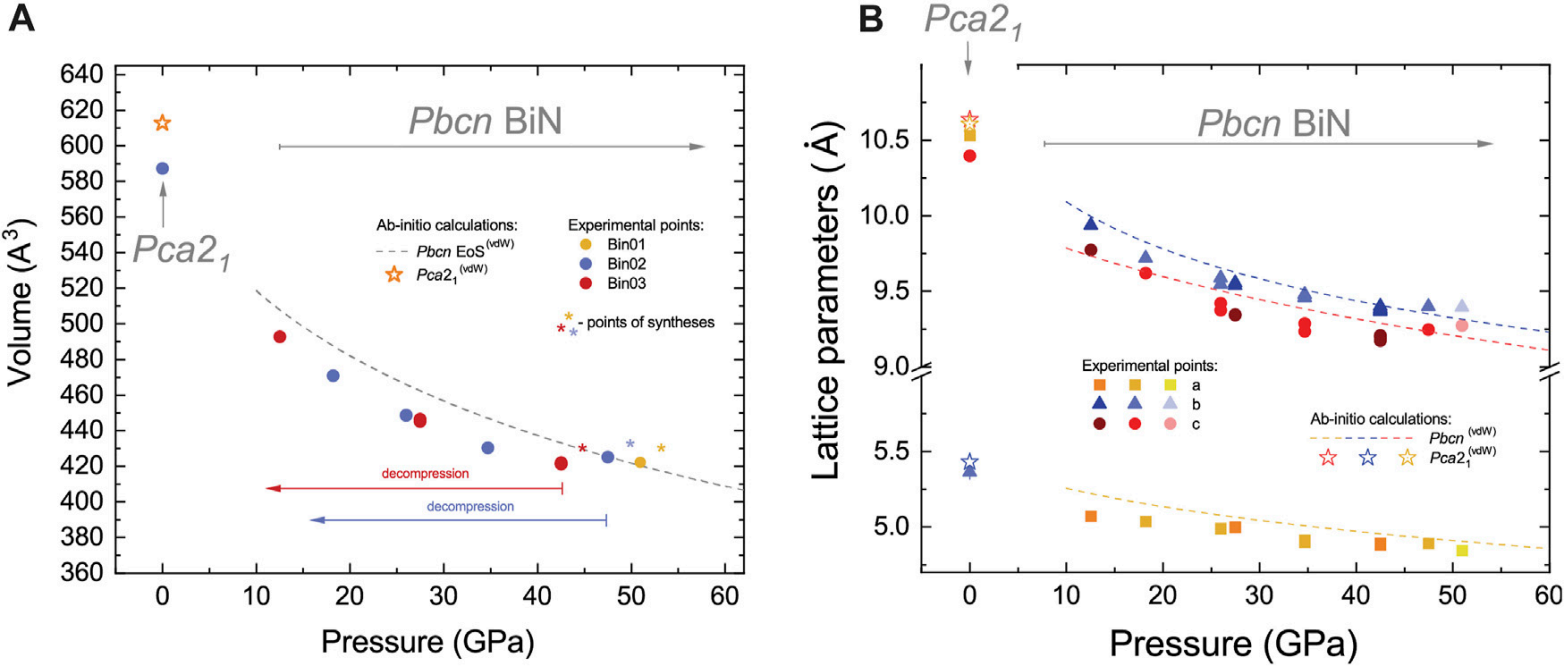
Variation in **(A)** the unit cell volume at ambient temperature and **(B)** the corresponding lattice parameters of *Pbcn* and *Pca*2_1_ BiN, as determined from experiments and compared with *ab initio* calculations. Star symbols in the vicinity of circles in **(A)** indicate the points of synthesis with the sample heated to ∼1800 (200) K and then subsequently quenched to the ambient temperature and measured. The gray dashed line corresponds to the theoretically calculated equation of state (EoS) of *Pbcn* BiN. The superscript (vdW) indicates *ab initio* calculations using van der Waals forces. These calculations provide a better agreement with the experimental data on the *Pca*2_1_ stability field (e.g., experimental volume) in comparison to calculations excluding this contribution. Considering the *Pbcn* phase stability field, the introduction of van der Waals forces has almost no effect on the calculation results. In both panels, we indicate independent sample loadings and datasets using individual color shading. The data from the individual datasets look consistent, but calculations, with an exception of higher pressures, tend to overestimate values as a function of pressure. Considering the *Pbcn* phase, at pressures above ∼38 GPa in **(B)**, we detect an anomaly of *b* and *c* unit cell parameter compression, resulting in a region of BiN strongly reduced compressibility. Error bars are within the size of the symbols.

The structural complexity of BiN finds a correlation with its peculiar compressibility, as shown in Figure 4 where we compare experimental data with *ab initio* predictions.

A closer look at experimental data provides the following observations. First, we detect an anomaly in the evolution of the lattice constants of *Pbcn* BiN at pressures above 35–38 GPa. Indeed, within the pressure range of 40–50 GPa, the volume of the unit cell does not change much. This peculiar behavior correlates with our observation of a strongly reduced compound volume compressibility, as confirmed in three different loadings and syntheses. We note that although nitrogen is far from being a perfect hydrostatic medium, laser heating tends to anneal undesirable deviatoric stress effects and micro-strains (Uts et al., 2013). Thus, we consider that our observations most likely reflect a real process of isostructural crystal chemistry readjustment within the compound at the points of synthesis indicated by star symbols in Figure 4A. Although these observations may look surprising, they are not impossible, as demonstrated in the case of another pnictogen-based compound, CoSb_3_ (Kraemer et al., 2007). In a moderate pressure range, CoSb_3_ exhibits a phenomenon of ‘self-insertion,’ resulting in an isostructural bond reconstruction under compression, resulting in an apparent strong decrease of compressibility. Although the presence of minor residual deviatoric stress and strain at the synthesis stage cannot be fully dismissed, it is essential to acknowledge that they might not be the sole factors contributing to our observations, and we require a careful analysis of theoretical and experimental results.

If we look at the bulk compressibility, the *ab initio* equation of state (EoS) seems to overestimate the experimental data at pressures below ∼47 GPa. The overestimation of the lattice parameters and unit cell volume in DFT-relaxed structures is known for other Bi-bearing compounds (Cheng and Ren, 2011; Li et al., 2018; Gao et al., 2021) and attributed to the imperfections in exchange-correlation calculations using the GGA functional. For the *Pbcn* phase, and for a pressure range of 10–80 GPa, the third-order Birch–Murnagham EoS (Birch, 1952) obtained from the calculated EoS results in parameters of *V*_*0*_ *=* 580(16) Å^3^, *K*_*0*_ = 64(19) GPa, and *K’* = 5.6(9), where the parameters correspond to the ambient pressure volume, isothermal bulk modulus at ambient pressure, and its pressure derivative. The theoretical *K*_*0*_ value is low, which is expected from a Bi-bearing compound (Akahama et al., 2002). The calculated theoretical volume of *Pca*2_1_ after relaxation at zero pressure is also overestimated and corresponds to 612.8 Å^3^ and 640.8 Å^3^ for calculations including van der Waals forces (A2) and those omitting them (A1), respectively. This contrast likely indicates a greater role of the forces for interatomic interactions within the lower pressure *Pca*2_1_ phase. Our calculations for *Pbcn* propose the suppression of van der Waals forces at higher pressures. Altogether, although our conventional calculations may not be perfect, we will show that they capture essential aspects of *Pbcn* BiN and the evolution of its crystal chemistry.

**FIGURE 5:**
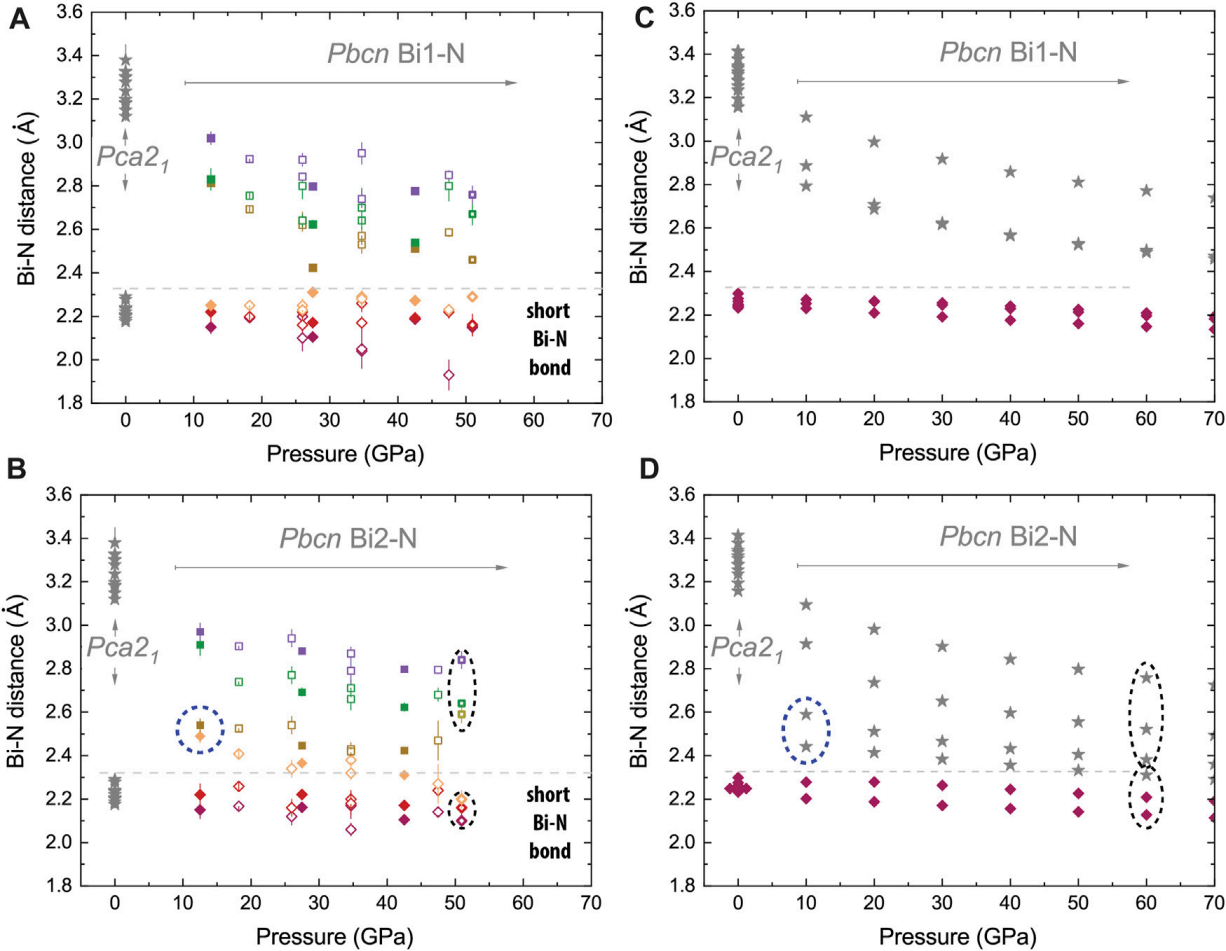
Effect of compression on next neighbor Bi–N distances in *Pbcn* BiN at ambient temperature. We compare experimental data **(A, B)** with the results of calculations involving van der Waals forces for *Pca*2_1_ and *Pbcn* phases shown in **(C, D)**. In **(A, B)** panels, we illustrate six different distances and use color and symbol linewidth to distinguish different syntheses and bonds. The gray lines shown in all panels are the eye guides helping to differentiate the shortest and longest Bi–N nearest neighbor distances (see also Figure 1). Considering the Bi1 site of the *Pbcn* phase, both the theory and experiment show a qualitative agreement. Bi1 is likely tightly bound to nitrogen and has three somewhat incompressible bonds and a coordination number CN = 3. Experimental data have some scatter, which can be related to the complexity of the experiment (single-crystal analysis of the polycrystalline aggregate) and non-hydrostatic stresses and strains (e.g., during decompression in N_2_); still, the trends of experimental data and the theory generally match for Bi1. We also see a good agreement between the *Pbcn* Bi2–N distances for shortest bonds. We see that at the lower pressure range (e.g., below 20 GPa), there are only two shortest bonds of the same distance, as in Bi1-N. Unlike Bi1-N, there is no third short bond for Bi2, but we see some close nitrogen neighbors at an intermediate distance of ∼2.5 Å. Our data highlighted by blue dashed ellipses suggest another type of Bi2-N *e* ^*-*^ orbit hybridization in comparison to Bi1-N. The longer bonds respond more eagerly to the stress change, and finally, we observe a change in hybridization with a change in the Bi2 coordination number from 3 to 4 on decompression with a crossover region in the vicinity of 40 GPa and 50 GPa for experiment and calculations, respectively.

**FIGURE 6:**
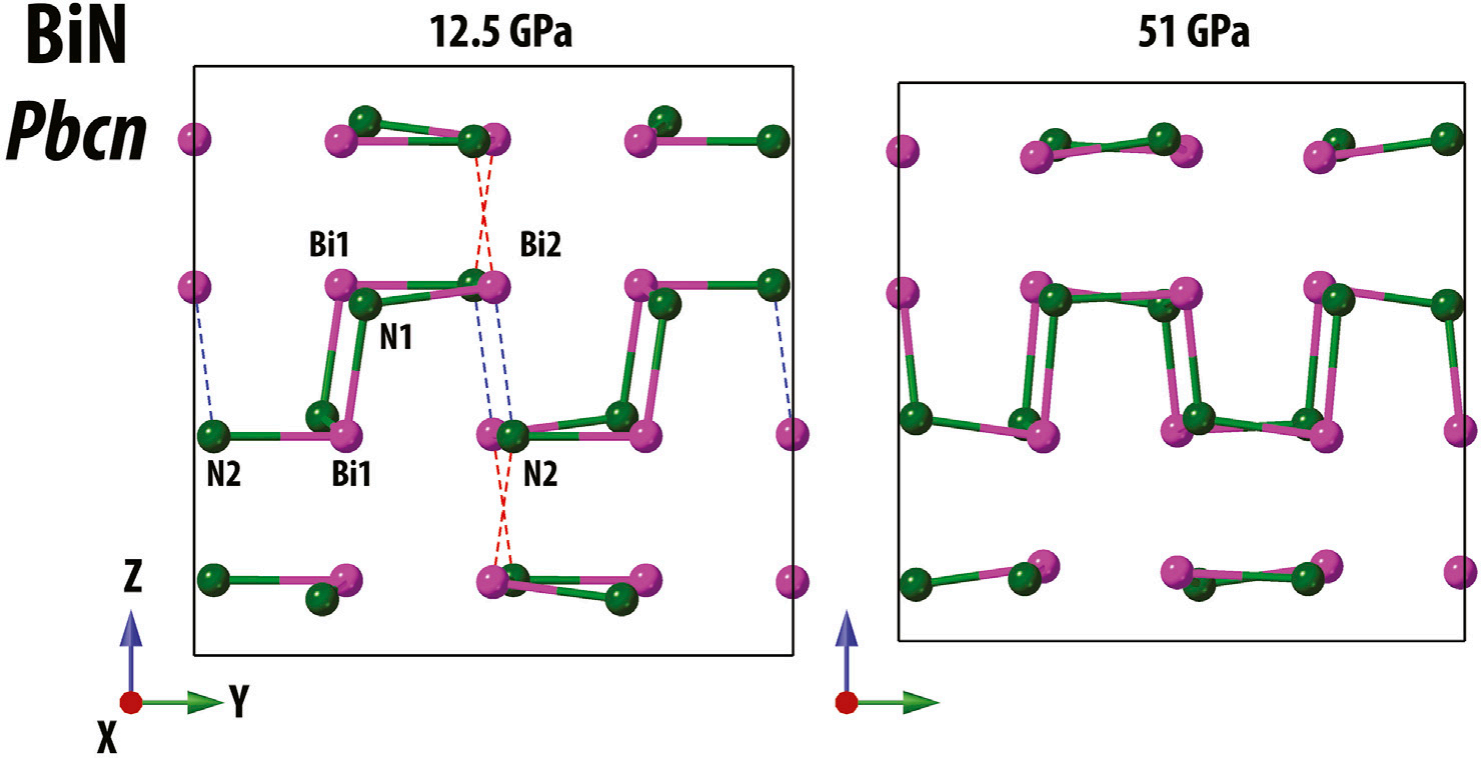
Illustration of Bi–N bonding in *Pbcn* Bi–N at ambient temperature with examples of structures solved at 12.5(4) and 51.0 (5) GPa. The structures are shown in the same scale. Short Bi–N bonds (distance below 2.35 Å) are shown as solid bi-color cylinders, and we indicate the longer, “intermediate” Bi2–N bonds as dashed lines (left panel, distance ∼2.5 Å).

As shown in Figure 5 for *Pbcn* BiN, both the theory and the experiment qualitatively agree with respect to the Bi1-N and Bi2-N distances and to the Bi1 and Bi2 coordination number change under stress. Bi1 has three shortest bonds (e.g., below 2.35 Å), the corresponding coordination number CN_Bi1_ = 3, and this situation does not change at different pressures. In contrast, the connectivity of Bi2 is different and does vary as a function of pressure. At ∼12.5(4) GPa, we observe two short Bi2-N bonds with lengths comparable to Bi1-N and two bonds of larger intermediate length (∼2.5–2.6 Å). The latter are illustrated using dashed ellipses in Figure 5 and using dashed lines in Figure 6. At lower pressures, the Bi–N layers are weakly bound through the indicated Bi2–N2 network with CN_Bi2_ = 4, but at higher pressures, the network is rearranged with the shortest Bi2–N bonds (below 2.35 Å) being enclosed within the Bi–N layers and resulting in CN_Bi2_ = 3 at 51.0(5) GPa. Additional graphical information comparing the unusual Bi near neighbor coordination at pressure points of 12.5(4) and 51.0(5) GPa is presented in the Supplementary Material. Considering *Pca*2_1_, we observe CN = 3 for all atoms.

We emphasize that the observation of CN_Bi2_ = 4 in *Pbcn* BiN is highly peculiar, and it is supported by both our experiment and calculations. It implies the same coordination number CN_N2_ = 4 for nitrogen occupying position N2 at 12.5(4) GPa and directs to a general discussion of the nitrogen strength as an electron acceptor in inorganic compounds and bonding type. Calculated Bader charges for BiN at 12.5(4) GPa (Bi: + [1.36–1.37], N: [1.36–1.37]) and electron localization function calculations (Supplementary Figure S7) demonstrate that ionic contribution to the Bi–N bonds is significant. This is in line with the difference in electronegativity between Bi and N atoms (Rahm et al., 2019; Tantardini and Oganov, 2021).

The presence of ionic bond may explain the intermediate coordination number CN_Bi2_ = CN_N2_ = 4 between *Pca*2_1_ and *Pbcn* phases if we think of the structures at the pressures of 0 GPa (CN_Bi_ = 3) and 51.0(5) GPa (CN_Bi_ = 3), respectively. A review of the literature will show various examples with the coordination number strictly increasing as a function of pressure (Prescher et al., 2017; Pakhomova et al., 2019; Spiekermann et al., 2019), and the case of BiN is a rare exception to this general empirical rule. A change of coordination is typically correlated with a change of electronic properties, and considering the pressure range, it fits well with our observation of unit cell-reduced compressibility and the anisotropic compressibility of the lattice parameters (Figure 4).

To conclude our discussion, the experimental evidence in combination with *ab initio* calculations demonstrates a peculiar compressibility related to the subtleties of binary BiN crystal chemistry. The polymorphs of this compound should have enhanced electronic properties, which may even stimulate the effect of thermoelectricity. Here, we direct to the similarities between *Pbcn* BiN and SnS, a compound made from elements belonging to the 14th and the 16th groups of the periodic table. SnS has been exhaustively investigated, and we note that it has a similar atomic arrangement band gap (∼1.3 eV) to *Pbcn* BiN and also exhibits thermoelectricity (Lanigan-Atkins et al., 2020), among many other fascinating properties (Tian et al., 2017; Ho et al., 2022).

At the same time, if we reflect on the *Pca*2_1_ polymorph of BiN, we find that it can be stabilized at ambient conditions in an inert atmosphere and potentially can be further stabilized by doping. In addition, the *Pca*2_1_ group is non-centrosymmetric, which leads to pyro- and piezo-electric properties. A review of the literature will show many materials attributed to the same space group exhibit piezoelectricity (Li et al., 2019; Silva et al., 2023). Altogether, the Bi–N system is highly interesting and will require further investigations.

## 4 Conclusion

With this investigation of the Bi–N system, we explored some of the blank spaces within the field of pnictogen chemistry and crystallography at extreme conditions. Using Bi and N_2_ as precursors, we synthesized the *Pbcn* polymorph of BiN and explored its phase stability upon decompression to ambient conditions. Upon quenching from high-pressure conditions, BiN is stabilized in the form of the non-centrosymmetric *Pca*2_1_ polymorph formed via a group–subgroup transformation from *Pbcn*.

Our results on the BiN compressibility revealed a region with an anomalous behavior of lattice parameters and unit cell volume as a function of pressure. After comparing the experimental results with the results of *ab initio* calculations, we confirmed a curious coordination number change for some of *Pbcn* crystallographic sites, one occupied by Bi and another one by N. In contrast to the conventional behavior of materials, where atomic coordination tends to increase at higher pressures, we see an intermediate CN = 4 confined to a broad range of pressures limited by CN = 3 (*Pca*2_1_ BiN) at 1 bar and CN = 3 (*Pbcn* BiN) at 40–50 GPa. We consider that the change in the coordination number related to the Bi–N bond rearrangement could be responsible for the observation of the BiN reduction of compressibility in the pressure range of 40–50 GPa.

Our study is one of many indicating the computational challenge for materials incorporating a significant concentration of heavy elements along with a large number of formula units per unit cell (e.g., Z = 16 for *Pca*21 and *Pbcn* BiN). We show that a conventional calculation approach is capable of uncovering important evidence but may not be fully sufficient to expose all the details of complex material behavior. Recent advances in the field of *ab initio* calculations are admirable, but many sophisticated techniques still have limited distribution due to limited access to the computational resources and extended duration of calculation. Improved accessibility to techniques like the continuous-time strong-coupling quantum Monte Carlo method coupled with state-of-the-art experimentation will considerably shorten the path between a synthesis discovery and a potential practical application. However, this is work for the future.

The case of BiN is special because this compound is formed from stable elements of the pnictogen group, with the highest contrast between constituents in terms of electron per group element. To the best of our knowledge, this is the first study investigating the crystal chemistry of BiN at ambient and extreme conditions. The reported findings are intriguing and expand our understanding of metal–nitrogen crystal chemistry in general and our knowledge of inter-pnictogen reactions in particular.

## Data Availability

The original contributions presented in the study are included in the article/Supplementary Material; further inquiries can be directed to the corresponding author.
