# Supplementary material for: High-pressure reactions between the pnictogens: the rediscovery of BiN
Source: Front Chem. 2023 Oct 12;11:1257942. doi: 10.3389/fchem.2023.1257942 (PMC10602720; doi:10.3389/fchem.2023.1257942)
Supplement: Supplementary file 6 [file Presentation1.pdf]

Supplementary Material

High-pressure reactions between the pnictogens: the rediscovery of BiN.

K. Glazyrin[1*], A. Aslandukov[2,3], A. Aslandukova[3], T. Fedotenko[1], S. Khandarkhaeva[3], D. Laniel[4], M. Bykov[5], L. Dubrovinskly[3]

[1]Deutsches Elektronen-Synchrotron DESY, Notkestr. 85, 22607 Hamburg

[2]Material Physics and Technology at Extreme Conditions, Laboratory of Crystallography, University of Bayreuth, Universitaetsstr. 30, Bayreuth, 95440, Germany

[3]Bayerisches Geoinstitut, University of Bayreuth, Universitätsstr. 30, 95447 Bayreuth, Germany

[4]Centre for Science at Extreme Conditions and School of Physics and Astronomy, University of Edinburgh, Edinburgh, UK

[5]Institute of Inorganic Chemistry, University of Cologne, Greinstr. 6, 50939 Cologne, Germany

Correspondence:
Corresponding Author
konstantin.glazyrin@desy.de

Supplementary Data

1 BiN structure: indexing and supporting information.

At the next pages we provide figures illustrating various steps of analysis process. First, we show specific slices of reciprocal space corresponding to *Pbcn* and *Pca*2_1 phases of BiN collected at 42.5(3) GPa (Figure S 1) and at 1 bar (Figure S 2), respectively. For data collection we used a small beam of 2·2 μm^2 (H·V, fullwidth at half maximum) at the focal spot we could isolate some individual domains corresponding to both phases and solve their structures. The slices represent a typical example of using single crystal methods with polycrystalline samples (overlap with diffraction coming from precursor materials, grains of the same phase but different orientation, etc).

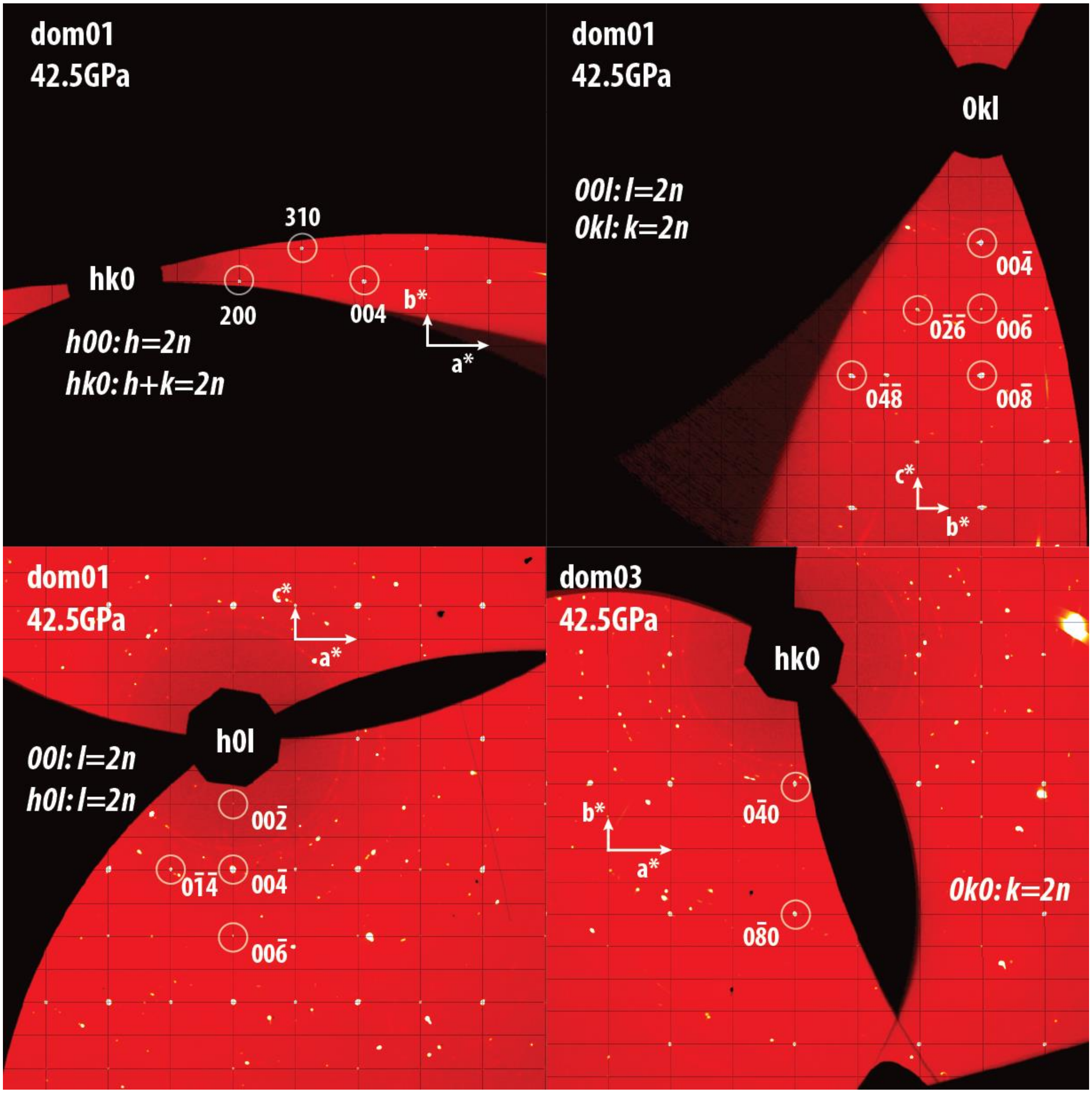

Figure S1 Information on reciprocal space collected from two individual domains (dom1, dom3). The domains belong to the same orthorhombic phase synthesized at 42.5(3) GPa from Bi and N_2 precursors. The indicated extinction rules correspond to *Pbcn* (S.G. 60). We also indicate a*, b* and c* reciprocal space basis vectors of the orthorhombic lattice. Additional signal present at the slices can be attributed to the presence of other domains of the same phase, to the signal of the environment (diamond Bragg or diffuse scattering) or the signal from the precursors. It was important to collect data with a small beam in order to boost the signal to noise ratio and avoid significant signal overlap.

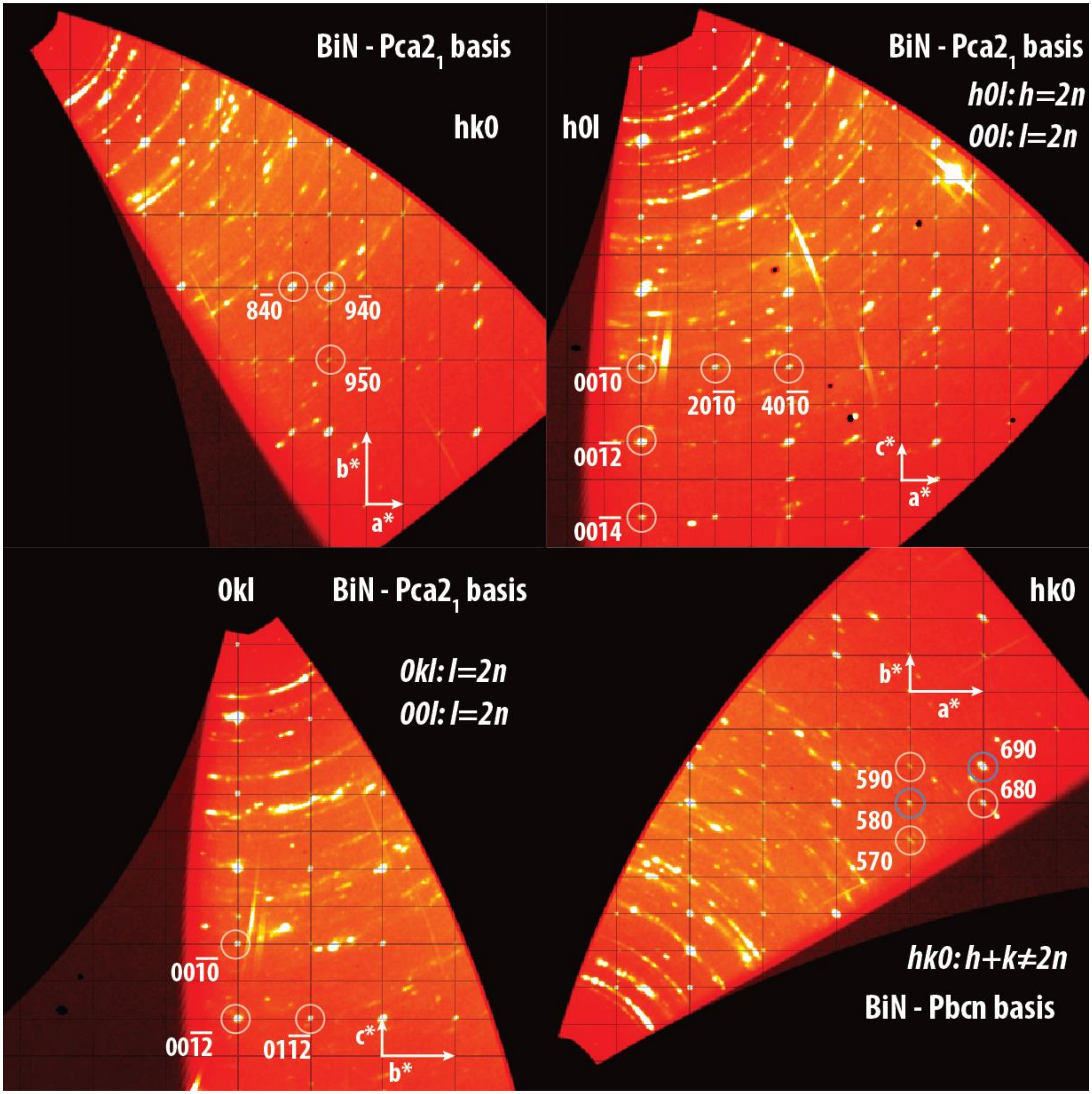

Figure S2 Information on reciprocal space collected from a single domain in different crystallographic basis. Upon decompression from ~10 GPa to 1 bar we saw an appearance of additional reflections contradicting extinction rules for *Pbcn* space group (lower right panel), we indicate those reflections using light blue circles. The structure quenched to 1 bar was finally indexed and solved as $Pca2_1$ (S.G. 29). Considering the choice between $Pca2_1$ over $Pbc2_1$, we selected the former as the standard setting choice. During decompression pressure was carefully released until we saw nitrogen Raman shift reaching 2330 cm^{-1} indicating ambient pressure (Ray et al., 2018).

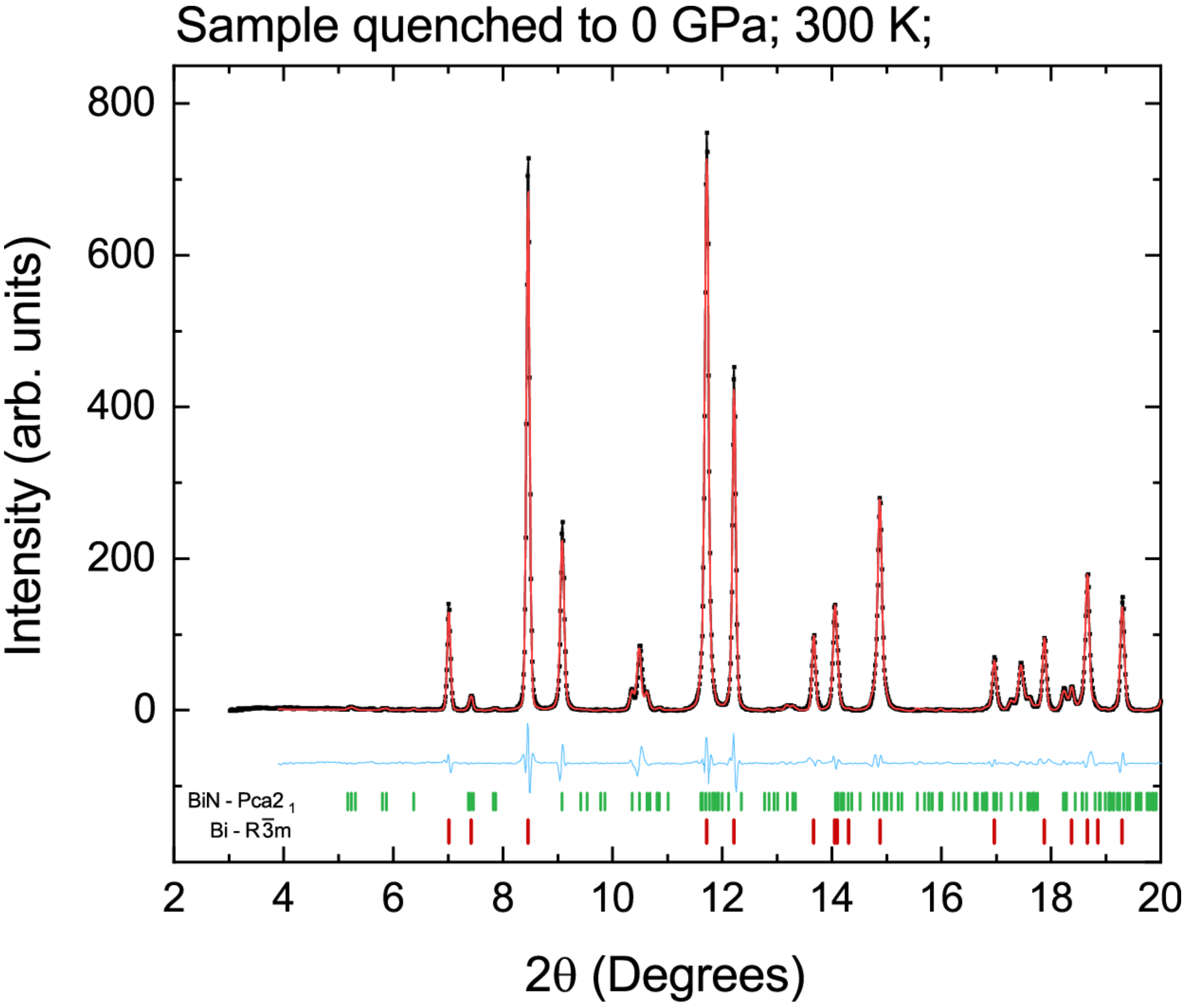

Figure S3 Le Bail profile matching for $Pca2_1$ phase quenched to 1 bar. In this case no reliable single crystal data collection was possible as sample got damaged during a Raman measuring attempt using 532 nm laser at ~40 mW power at a focal spot. Pure Bi contributes to the strongest recorded signal, but we also observe peaks which we can attribute to the BiN in $Pca2_1$ form.

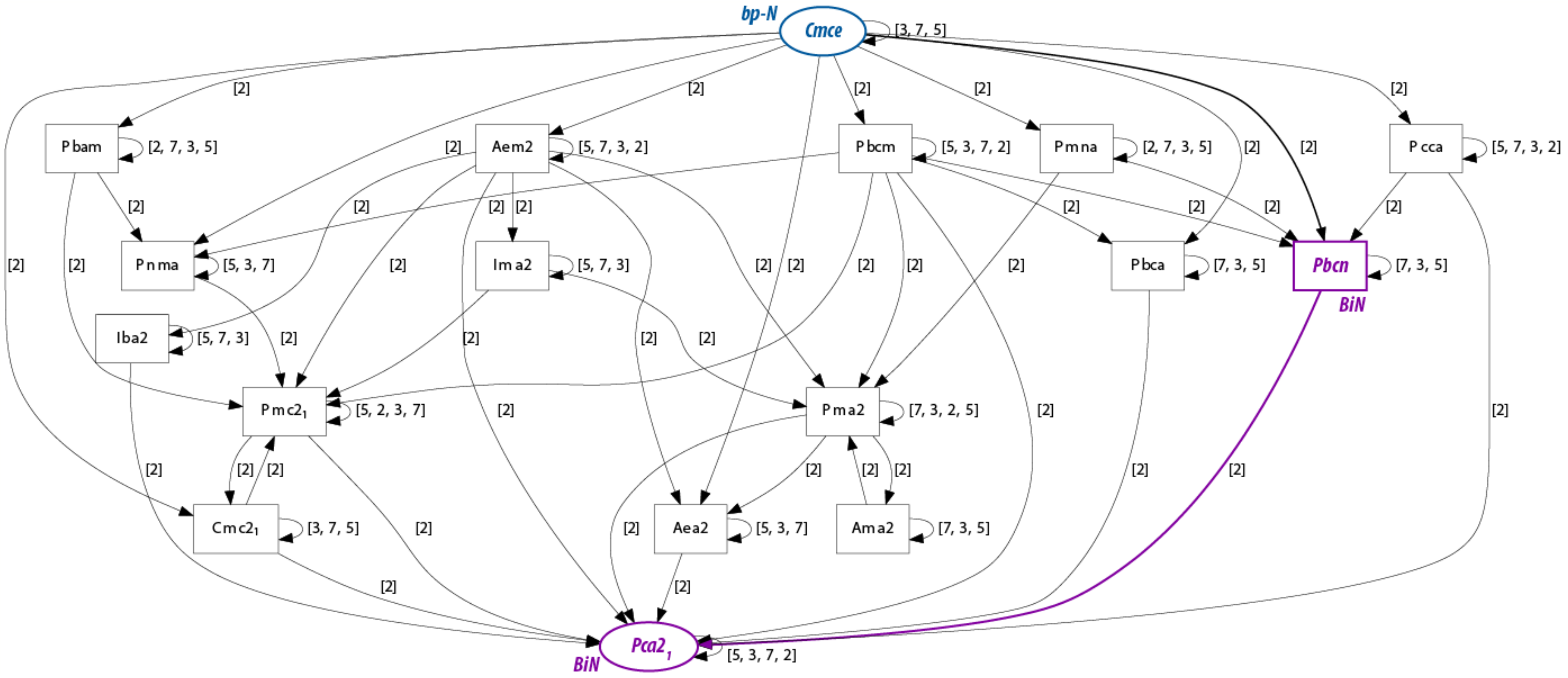

Figure S4 Group-subgroup relationship between *Cmce* and *Pca*2_1 space groups plotted with help of Bilbao Crystallographic Server (Aroyo et al., 2006b, 2006a). We observe a close relation between *Cmce* space group which was attributed to black phosphorus phase of nitrogen: bp-N (Laniel et al., 2020) as well as *Pbcn* and *Pca*2_1 of BiN which may indicate the a yet to be discovered binary or ternary pnictogen compounds may form within the same or expanded group-subgroup space in a contrast to cubic phases of AsN and cubic gauche N (cg-N) which, based on their crystal chemistry, a separate class of compounds (Eremets et al., 2004; Ceppatelli et al., 2022)

2 Calculation of electronic density of state and electron localization function (ELF), phonon dispersion curves, mechanical stability of Bi-N polymorphs.

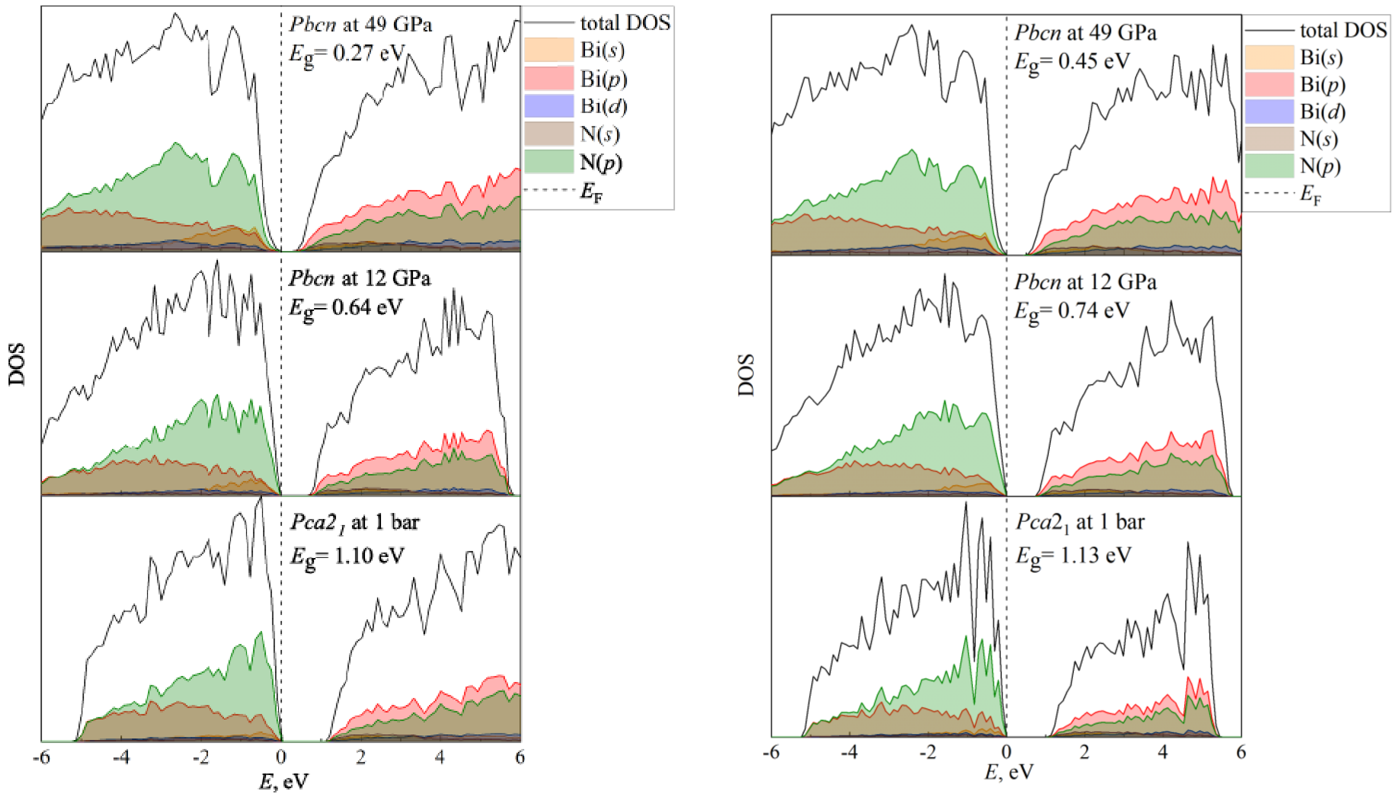

Figure S5 Results of *ab-initio* calculations on the electronic density of states of the two BiN polymorphs at various pressures. The calculations' details can be found in the main manuscript. Calculations indicate the semiconducting behavior of BiN and a band gap (E_g) reduction as a function of compression. Position $E=0$ corresponds to the position of the Fermi energy level. (Left) Shows calculations conducted without the inclusion of van der Waals contributions and to the (Right) we show the calculations employing the latter type of interaction. Obviously, although some details differ, both approximations are in good agreement, e.g. band gap values, compression induced suppression of the band gap.

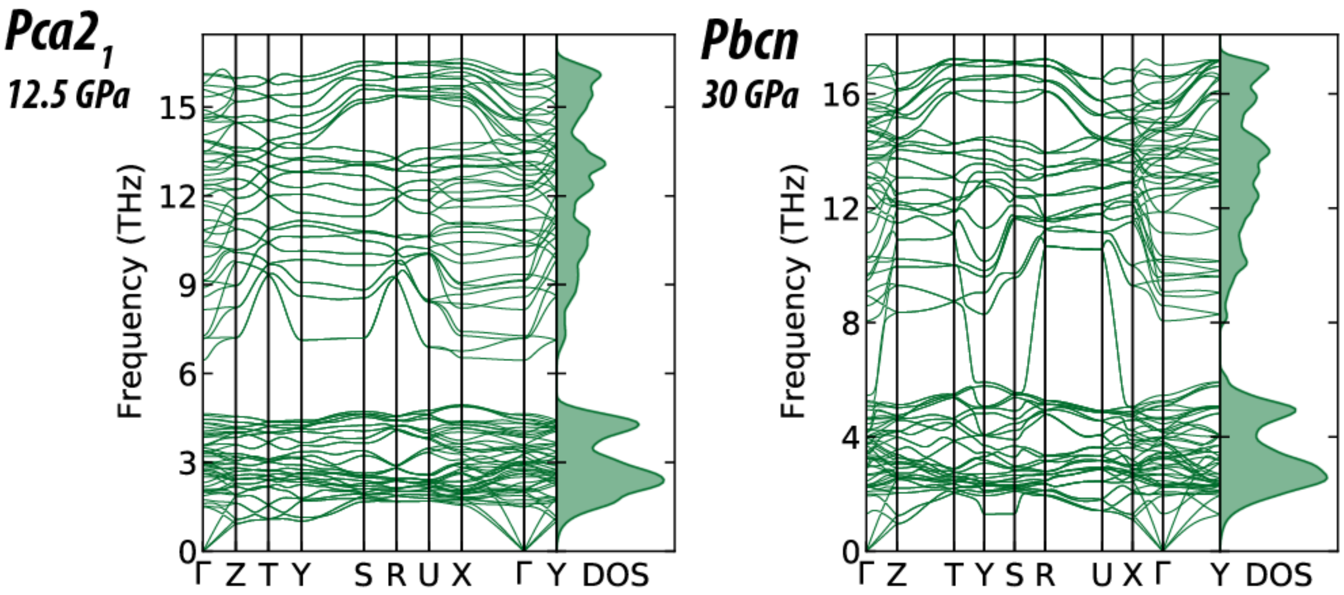

Figure S6 Our calculations of phonon dispersion curves conducted for the pressure points of 12.5 and 30 GPa for *Pca*2$_1$ and *Pbcn* BiN polymorphs, respectively, provide additional support to our enthalpy calculations and experimental data (see Figure 3 of the manuscript).

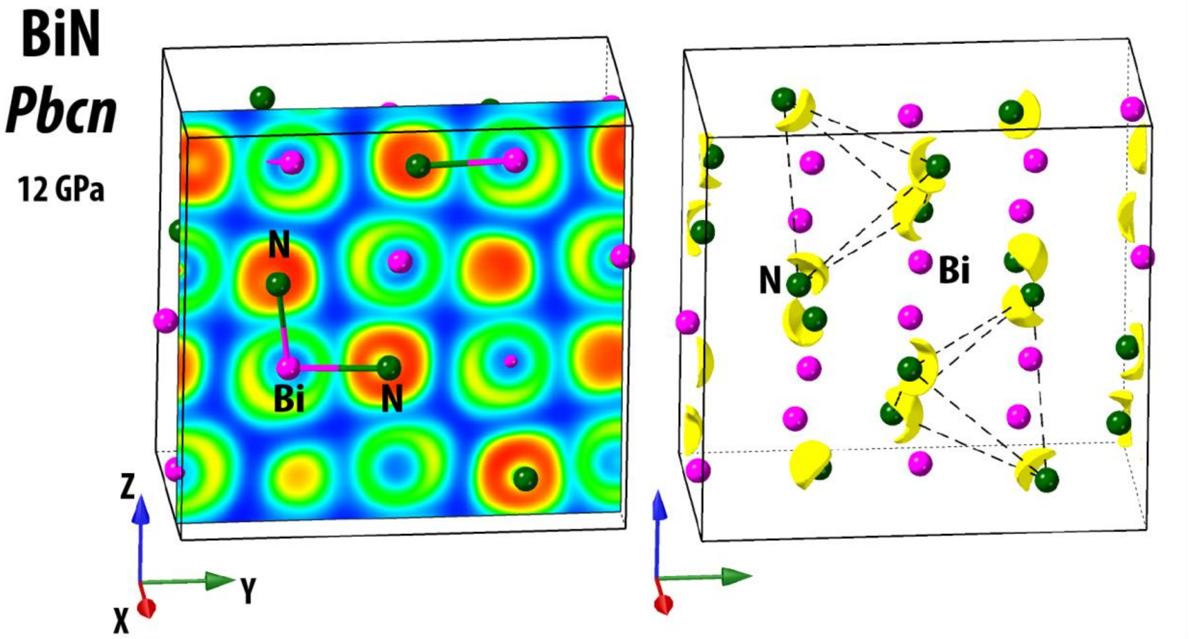

Figure S7 Results of electron localization function calculation (ELF) for *Pbcn* BiN at 12 GPa. To the left we show a ELF cross-section parallel to (100) offset close to the indicated Bi and N sites. Here we show the full scale of ELF values. Our calculations suggest a pronounced ionic Bi-N bond character. To the right, similar to (Savin et al., 1997; Laniel et al., 2022) we demonstrate electronic density above 0.84 $e^-/Å^3$. The yellow features likely correspond to lone pairs at nitrogen sites. These shapes are deformed in such a way that they face the center of a tetrahedron formed by nitrogen atoms indicated by dashed lines. Very similar features are visible in our ELF calculations for 49 GPa.

Additionally, we introduce calculations estimating the mechanical stability of the BiN phases (Mouhat & Coudert 2014). The calculated elastic tensors C_{ij} for both polymorphs are shown in Tables 1 and 2 below.

Table M1. Calculated elastic tensor C_{ij} (in GPa) of *Pbcn*-BiN at 49 GPa.

C_{ij}	1	2	3	4	5	6
1	376.0	186.7	171.2	0	0	0
2	186.7	445.0	108.8	0	0	0
3	171.2	108.8	459.9	0	0	0
4	0	0	0	105.0	0	0
5	0	0	0	0	37.9	0
6	0	0	0	0	0	75.8

Table M2. Elastic tensor C_{ij} (in GPa) of *Pca*2$_1$-BiN at 1 bar.

C_{ij}	1	2	3	4	5	6
1	44.6	13.0	11.1	0	0	0
2	13.0	54.9	11.2	0	0	0
3	11.1	11.2	36.6	0	0	0
4	0	0	0	26.3	0	0
5	0	0	0	0	20.6	0
6	0	0	0	0	0	22.8

The mechanical stability criteria for an orthorhombic system are:

$C_{11} > 0$,

$C_{11}C_{22} > C_{12}^2$,

$C_{11}C_{22}C_{33} + 2C_{12}C_{13}C_{23} - C_{11}C_{23}^2 - C_{22}C_{13}^2 - C_{33}C_{12}^2 > 0$,

$C_{44} > 0$, $C_{55} > 0$, $C_{66} > 0$.

The values of C_{ij} satisfy all of the criteria for mechanical stability for the corresponding pressure points.

3 Tables with experimental BiN crystallographic data

Tables are presented below as a function of increasing pressure. In the tables below, we present various information including unit lattice parameters, parameters from structure solution and refinement. The tables provide information from CIF files submitted to the Cambridge Crystallographic Data Centre (CCDC) under

deposition numbers 2280721, 2280722, 2280723, 2280724, 2280725, 2280726, 2280727, 2280728, 2280729. The limits in H, K, L and θ correspond to CIF file information fields representing minimum and the maximum values of the corresponding Miller indexes (e.g. link) as well as the minimum and maximum θ angles (e.g. link) in degrees for the measured diffraction intensities.

3.1 *Pbcn* – 12.5(4) GPa

Compound:	BiN
Pressure:	12.5(4) GPa
Wavelength:	0.4839 Å
Space group:	*Pbcn*, S.G. #60
Z:	16

Lattice parameters

a, Å	b, Å	c, Å	V, Å^3
5.0725(12)	9.938(8)	9.7748(15)	492.8(4)

Refinement information

R1, I>2σ(I)	3.3 %	ρ_{min}, ρ_{max}, $e^-/Å^3$	-0.75, 0.64
wR2, I>2σ(I)	9.1 %		-5 ≤ H ≤ 4
N_{par}/N_{obs}	27/144	2.84°≤ θ ≤17.12°	-4 ≤ K ≤ 6
			-8 ≤ L ≤ 9

Structural information

site	*x*	*y*	*z*	ADP
Bi1	0.2276(3)	0.2527(3)	0.1277(1)	0.0220(10)*
Bi2	0.2416(1)	0.4974(3)	0.3752(1)	0.0219(11)*
N1	0.360(3)	0.214(3)	0.4043(13)	0.035(4)
N2	0.160(5)	0.034(4)	0.1275(16)	0.059(6)

I – signal intensity; *σ(I)* – standard deviation of *I*; *R1, wR2* – crystallographic R-factors; N_{par}/N_{obs} – ratio between number of parameters to number of observed reflections for *I>2σ(I)*; ρ_{min}, ρ_{max} – minimum and maximum values of residual electronic density; ADP – atomic displacement parameters (e.g. * - U_{equiv}., otherwise - U_{iso}).

3.2 *Pbcn* – 18.2(2) GPa

Compound:	BiN			
Pressure:	18.2(2) GPa			
Wavelength:	0.2904 Å			
Space group:	*Pbcn*, S.G. #60			
Z:	16			
**Lattice parameters**				
a, Å	b, Å		c, Å	V, $Å^3$
5.0366(4)	9.7204(15)		9.621(3)	471.03(18)
**Refinement information**				
R1, I>2σ(I)	3.7 %		ρ_{min}, ρ_{max}, $e^-/Å^3$	-2.34, 3.32
wR2, I>2σ(I)	8.6 %			-9 ≤ H ≤ 9
N_{par}/N_{obs}	27/467		1.86°≤ θ ≤16.66°	-17 ≤ K ≤ 16<br>-14 ≤ L ≤ 10
**Structural information**				
site	*X*	*y*	*z*	ADP
Bi1	0.2234(1)	0.2542(1)	0.1292(1)	0.0099(2)*
Bi2	0.2382(1)	0.4956(1)	0.3757(1)	0.0099(2)*
N1	0.3496(20)	0.2216(11)	0.4045(17)	0.010(2)
N2	0.164(2)	0.0239(15)	0.120(2)	0.019(2)

I – signal intensity; *σ(I)* – standard deviation of *I*; *R1, wR2* – crystallographic R-factors; N_{par}/N_{obs} – ratio between number of parameters to number of observed reflections for *I>2σ(I)*; ρ_{min}, ρ_{max} – minimum and maximum values of residual electronic density; ADP – atomic displacement parameters (e.g. * - U_{equiv}., otherwise - U_{iso}).

3.3 *Pbcn* – 26.0(15) GPa

Compound:	BiN		
Pressure:	26.0(15) GPa		
Wavelength:	0.2904 Å		
Space group:	*Pbcn*, S.G. #60		
Z:	16		
Lattice parameters			
a, Å	b, Å	c, Å	V, Å^3
4.9919(10)	9.588(7)	9.376(3)	448.8(4)
Refinement information			
R1, I>2σ(I)	5.7 %	ρ_{min}, ρ_{max}, $e^-/Å^3$	-3.91, 2.95
wR2, I>2σ(I)	12.1 %		-9 ≤ H ≤ 8
N_{par}/N_{obs}	27/367	1.77°≤ θ ≤17.42°	-10 ≤ K ≤ 11<br>-17 ≤ L ≤ 15

Structural information

site	*x*	*y*	*z*	ADP
Bi1	0.22186(15)	0.2540(3)	0.13128(8)	0.0169(4)*
Bi2	0.23535(13)	0.4937(3)	0.37669(9)	0.0160(5)*
N1	0.151(6)	0.046(5)	0.116(3)	0.032(6)
N2	0.357(4)	0.210(3)	0.397(2)	0.016(4)

I – signal intensity; *σ(I)* – standard deviation of *I*; *R1, wR2* – crystallographic R-factors; N_{par}/N_{obs} – ratio between number of parameters to number of observed reflections for *I>2σ(I)*; ρ_{min}, ρ_{max} – minimum and maximum values of residual electronic density; ADP – atomic displacement parameters (e.g. * - U_{equiv}., otherwise - U_{iso}).

3.4 *Pbcn* – 27.0(2) GPa

Compound:	BiN		
Pressure:	27.5(2) GPa		
Wavelength:	0.4839 Å		
Space group:	*Pbcn*, S.G. #60		
Z:	16		
Lattice parameters			
a, Å	b, Å	c, Å	V, $Å^3$
5.0002(7)	9.556(4)	9.3468(12)	446.61(18)
Refinement information			
R1, I>2σ(I)	3.3 %	ρ_{min}, ρ_{max}, $e^-/Å^3$	-1.48, 2.13
wR2, I>2σ(I)	7.7 %	$2.97° \leq \theta \leq 17.72°$	$-5 \leq H \leq 5$
N_{par}/N_{obs}	27/123		$-7 \leq K \leq 6$<br>$-10 \leq L \leq 10$

Structural information

site	*x*	*y*	*z*	ADP
Bi1	0.2221(3)	0.2546(1)	0.13112(8)	0.0128(8)*
Bi2	0.2356(2)	0.4935(1)	0.37671(6)	0.0133(8)*
N1	0.356(3)	0.2201(17)	0.3996(14)	0.013(4)
N2	0.163(3)	0.028(2)	0.1228(12)	0.016(5)

I – signal intensity; *σ(I)* – standard deviation of *I*; *R1, wR2* – crystallographic R-factors; N_{par}/N_{obs} – ratio between number of parameters to number of observed reflections for *I>2σ(I)*; ρ_{min}, ρ_{max} – minimum and maximum values of residual electronic density; ADP – atomic displacement parameters (e.g. * - U_{equiv}., otherwise - U_{iso}).

3.5 *Pbcn* – 34.7(2) GPa

Compound:	BiN
Pressure:	34.7(2) GPa
Wavelength:	0.2904 Å
Space group:	*Pbcn*, S.G. #60
Z:	16

Lattice parameters

a, Å	b, Å	c, Å	V, Å^3
4.9002(18)	9.458(4)	9.287(13)	430.5(6)

Refinement information

R1, I>2σ(I)	6.4 %	ρ_{min}, ρ_{max}, $e^-/Å^3$	-5.61, 4.74
wR2, I>2σ(I)	11.8 %		-7 ≤ H ≤ 7
N_{par}/N_{obs}	26/254	1.76°≤ θ ≤17.77°	-14 ≤ K ≤ 14<br>-9 ≤ L ≤ 9

Structural information

site	*x*	*y*	*z*	ADP
Bi1	0.2206(2)	0.2556(3)	0.1315(4)	0.0088(9)*
Bi2	0.2327(2)	0.4925(3)	0.3762(5)	0.0067(8)*
N1	0.150(5)	0.022(3)	0.121(7)	0.003(3)
N2	0.328(5)	0.221(4)	0.411(7)	0.003(3)

I – signal intensity; *σ(I)* – standard deviation of *I*; *R1, wR2* – crystallographic R-factors; N_{par}/N_{obs} – ratio between number of parameters to number of observed reflections for *I>2σ(I)*; ρ_{min}, ρ_{max} – minimum and maximum values of residual electronic density; ADP – atomic displacement parameters (e.g. * - U_{equiv}., otherwise - U_{iso}).

3.6 ***Pbcn*** **– 42.5(3) GPa**

Compound:	BiN		
Pressure:	42.5(3) GPa		
Wavelength:	0.2909 Å		
Space group:	*Pbcn*, S.G. #60		
Z:	16		
Lattice parameters			
a, Å	b, Å	c, Å	V, Å^3
4.8941(5)	9.4020(10)	9.176(4)	422.22(19)
Refinement information			
R1, I>2σ(I)	2.6 %	ρ_{min}, ρ_{max}, $e^-/Å^3$	-1.84, 1.87
wR2, I>2σ(I)	5.3 %	1.92°≤ θ ≤17.28°	-8 ≤ H ≤ 8<br>-15 ≤ K ≤ 16<br>-8 ≤ L ≤ 10
N_{par}/N_{obs}	37/518		

Structural information

site	*x*	*y*	*z*	ADP
Bi1	0.21914(6)	0.25581(3)	0.13253(6)	0.0048(2)*
Bi2	0.23128(5)	0.49159(3)	0.37659(7)	0.0049(2)*
N1	0.3497(13)	0.2214(8)	0.3964(17)	0.010(4)*
N2	0.1616(13)	0.0240(8)	0.1242(19)	0.006(4)*

I – signal intensity; *σ(I)* – standard deviation of *I*; *R1, wR2* – crystallographic R-factors; N_{par}/N_{obs} – ratio between number of parameters to number of observed reflections for *I>2σ(I)*; ρ_{min}, ρ_{max} – minimum and maximum values of residual electronic density; ADP – atomic displacement parameters (e.g. * - U_{equiv}., otherwise - U_{iso}).

3.7 *Pbcn* – 47.5(2) GPa

Compound:	BiN
Pressure:	47.5(2) GPa
Wavelength:	0.2904 Å
Space group:	*Pbcn*, S.G. #60
Z:	16

Lattice parameters

a, Å	b, Å	c, Å	V, $Å^3$
4.8911(19)	9.471(3)	9.202(15)	426.3(7)

Refinement information

$R1, I>2\sigma(I)$	3.7 %	$\rho_{min}, \rho_{max}, e^-/Å^3$	-1.87, 1.90
$wR2, I>2\sigma(I)$	7.2 %		$-10 \leq H \leq 9$
N_{par}/N_{obs}	27/246	$1.91° \leq \theta \leq 17.58°$	$-18 \leq K \leq 18$<br>$-4 \leq L \leq 5$

Structural information

site	*x*	*y*	*z*	ADP
Bi1	0.2207(1)	0.2564(1)	0.1324(3)	0.0150(11)*
Bi2	0.2315(1)	0.4917(1)	0.3768(5)	0.0169(11)*
N1	0.346(3)	0.2198(16)	0.426(6)	0.013(3)
N2	0.164(3)	0.0231(16)	0.136(7)	0.014(2)

I – signal intensity; $\sigma(I)$ – standard deviation of *I*; *R1, wR2* – crystallographic R-factors; N_{par}/N_{obs} – ratio between number of parameters to number of observed reflections for $I>2\sigma(I)$; ρ_{min}, ρ_{max} – minimum and maximum values of residual electronic density; ADP – atomic displacement parameters (e.g. * - U_{equiv}., otherwise - U_{iso}).

3.8 *Pbcn* – 51.0(5) GPa

Compound:	BiN		
Pressure:	51.0(5) GPa		
Wavelength:	0.2904 Å		
Space group:	*Pbcn*, S.G. #60		
Z:	16		
**Lattice parameters**			
a, Å	b, Å	c, Å	V, Å^3
4.8613(19)	9.394(3)	9.249(15)	422.4(7)
**Refinement information**			
R1, I>2σ(I)	7.5 %	ρ_{min}, ρ_{max}, $e^-/Å^3$	-5.31, 5.27
wR2, I>2σ(I)	13.2 %	1.77°≤ θ ≤17.71°	-7 ≤ H ≤ 7<br>-16 ≤ K ≤ 15<br>-4 ≤ L ≤ 10
N_{par}/N_{obs}	26/205		

Structural information

site	*x*	*y*	*z*	ADP
Bi1	0.2205(3)	0.2558(2)	0.1324(4)	0.0092(9)*
Bi2	0.2314(3)	0.4923(2)	0.3756(6)	0.0070(10)*
N1	0.344(6)	0.223(4)	0.394(11)	0.008(4)
N2	0.155(7)	0.031(4)	0.127(10)	0.008(4)

I – signal intensity; *σ(I)* – standard deviation of *I*; *R1, wR2* – crystallographic R-factors; N_{par}/N_{obs} – ratio between number of parameters to number of observed reflections for *I>2σ(I)*; ρ_{min}, ρ_{max} – minimum and maximum values of residual electronic density; ADP – atomic displacement parameters (e.g. * - U_{equiv}., otherwise - U_{iso}).

4 Bi1-N and Bi2 bonding in *Pbcn* BiN.

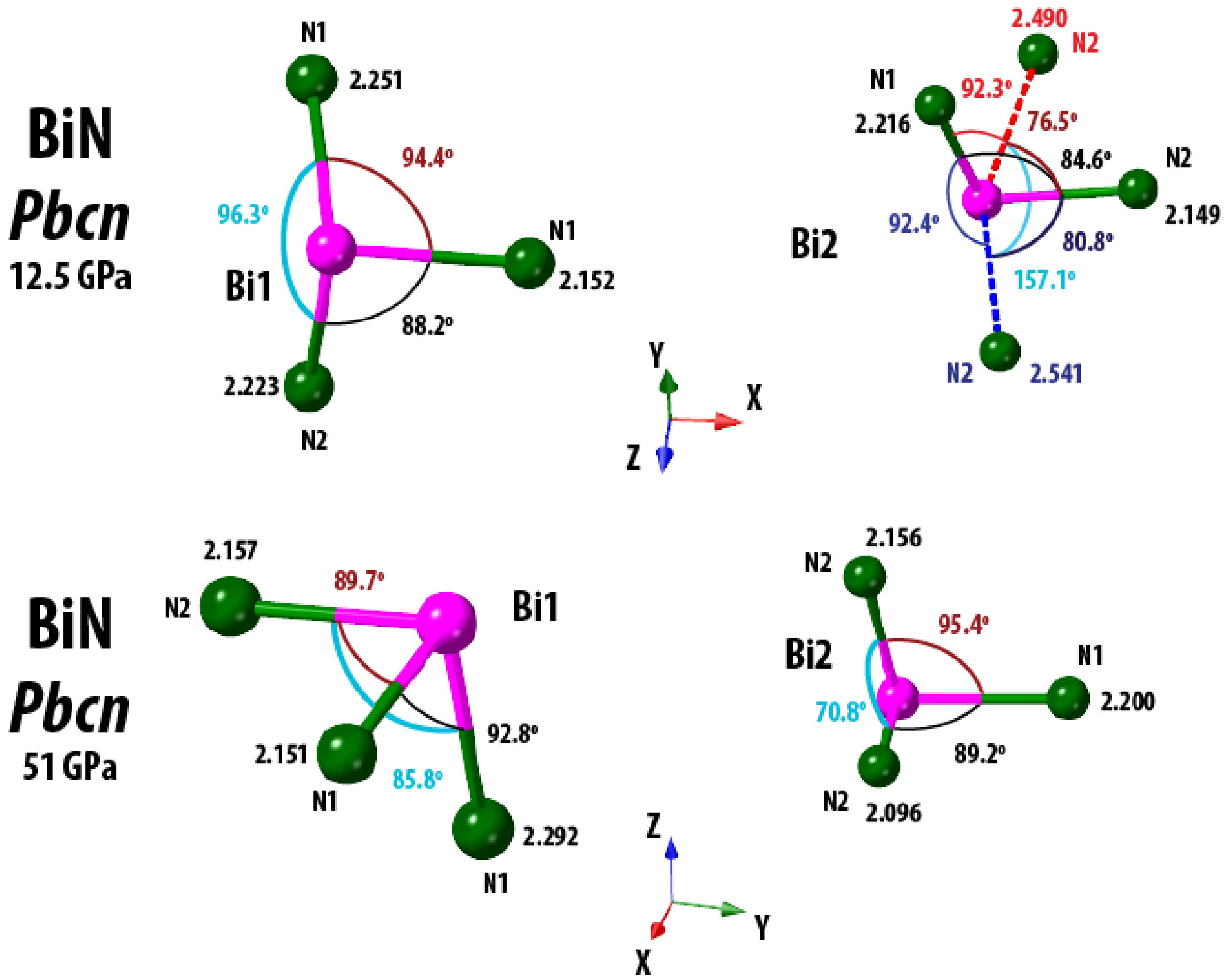

Figure S7 Bi1 and Bi2 crystallographic sites of *Pbcn* BiN and their nearest neighbor environment at different pressures. We indicate individual atoms, distances between the nitrogen and the bismuth atoms as well as angles between the segments. At the pressure of 12.5(3) GPa we observe a tetragonal coordination for Bi1 corresponding to a coordination number CN=3. Bi2 site is peculiar, and at the same pressure it can be described as a quarter of a distorted octahedron with CN=4. At a higher pressure of 51.0(5) GPa the Bi2 coordination number becomes CN=3. As we show in the Figure 6 of the main text, the blue dashed segment becomes a Bi2-N short bond within the Bi-N layers (XY plane). The red dashed line indicates an “intermediate” Bi2-N bond connecting individual layers of Bi-N which cease to exist at 51.0(5) GPa. The axes shown in figure correspond to the directions of unit cell basis for each given pressure. We do not show error bars for the simplicity of representation. This information can be easily extracted from the accompanying crystallographic information files (CIF) submitted with the publication.

References.

Aroyo, M. I., Kirov, A., Capillas, C., Perez-Mato, J. M., and Wondratschek, H. (2006a). Bilbao Crystallographic Server. II. Representations of crystallographic point groups and space groups. *Acta Crystallographica,* 62, 115–128. doi: 10.1107/S0108767305040286.

Aroyo, M. I., Perez-Mato, J. M., Capillas, C., Kroumova, E., Ivantchev, S., Madariaga, G., et al. (2006b). Bilbao Crystallographic Server: I. Databases and crystallographic computing programs. *Zeitschrift fur Krist.* 221, 15–27. doi: 10.1524/ZKRI.2006.221.1.15/MACHINEREADABLECITATION/RIS.

Ceppatelli, M., Scelta, D., Serrano-Ruiz, M., Dziubek, K., Morana, M., Svitlyk, V., et al. (2022). Single-Bonded Cubic AsN from High-Pressure and High-Temperature Chemical Reactivity of Arsenic and Nitrogen. *Angew. Chemie Int. Ed.* 61, e202114191. doi: 10.1002/ANIE.202114191.

Eremets, M. I., Gavriliuk, A. G., Trojan, I. A., Dzivenko, D. A., and Boehler, R. (2004). Single-bonded cubic form of nitrogen. *Nat. Mater. 2004 38* 3, 558–563. doi: 10.1038/nmat1146.

Laniel, D., Trybel, F., Néri, A., Yin, Y., Aslandukov, A., Fedotenko, T., et al. (2022). Revealing Phosphorus Nitrides up to the Megabar Regime: Synthesis of α′-P3N5, δ-P3N5 and PN2. *Chem. – A Eur. J.* 28, e202201998. doi: 10.1002/CHEM.202201998.

Laniel, D., Winkler, B., Fedotenko, T., Pakhomova, A., Chariton, S., Milman, V., et al. (2020). High-Pressure Polymeric Nitrogen Allotrope with the Black Phosphorus Structure. *Phys. Rev. Lett.* 124, 216001. doi: 10.1103/PhysRevLett.124.216001.

Mouhat, F., & Coudert, F. X. (2014). Necessary and sufficient elastic stability conditions in various crystal systems. *Physical review B*, 90(22), 224104 doi:10.1103/PhysRevB.90.224104

Ray, P., Xu, E., Crespi, V. H., Badding, J. V., and Lueking, A. D. (2018). In situ vibrational spectroscopy of adsorbed nitrogen in porous carbon materials. *Phys. Chem. Chem. Phys.* 20, 15411–15418. doi: 10.1039/C8CP01790E.

Savin, A., Nesper, R., Wengert, S., and Fässler, T. F. (1997). ELF: The Electron Localization Function. *Angew. Chemie Int. Ed. English* 36, 1808–1832. doi: 10.1002/ANIE.199718081.
