# Supplementary material for: High-pressure reactions between the pnictogens: the rediscovery of BiN
Source: Front Chem. 2023 Oct 12;11:1257942. doi: 10.3389/fchem.2023.1257942 (PMC10602720; doi:10.3389/fchem.2023.1257942)

checkCIF/PLATON report

Structure factors have been supplied for datablock(s) I

THIS REPORT IS FOR GUIDANCE ONLY. IF USED AS PART OF A REVIEW PROCEDURE FOR PUBLICATION, IT SHOULD NOT REPLACE THE EXPERTISE OF AN EXPERIENCED CRYSTALLOGRAPHIC REFEREE.

No syntax errors found. CIF dictionary Interpreting this report

Datablock: I

```
Bond precision:   Bi- N = 0.0418 A         Wavelength=0.29040

Cell:               a=4.9002(18)       b=9.458(4)      c=9.287(13)
                    alpha=90           beta=90         gamma=90
Temperature:        293 K

                 Calculated                  Reported
Volume           430.4(6)                    430.5(6)
Space group      P b c n                     P b c n
Hall group       -P 2n 2ab                   -P -2xab;-2
Moiety formula   Bi2 N2                      ?
Sum formula      Bi2 N2                      Bi1 N1
Mr               445.98                      223.00
Dx,g cm-3        13.765                      13.763
Z                8                           16
Mu (mm-1)        16.105                      16.307
F000             1440.0                      1440.0
F000'            1426.84
h,k,lmax         10,19,19                    7,14,9
Nref             2101                        437
Tmin,Tmax                                    0.555,1.000
Tmin'

Correction method= # Reported T Limits: Tmin=0.555 Tmax=1.000
AbsCorr = MULTI-SCAN

Data completeness= 0.208           Theta(max)= 17.770

R(reflections)= 0.0640( 254)                      wR2(reflections)=
                                                  0.1290( 437)
S = 1.230                  Npar= 26
```

The following ALERTS were generated. Each ALERT has the format
test-name_ALERT_alert-type_alert-level.
Click on the hyperlinks for more details of the test.

Alert level A

PLAT029_ALERT_3_A _diffrn_measured_fraction_theta_full value Low . 0.400 Why?

Author Response: Multigrain sample in a diamond anvil cell (DAC). Aperture is limited by the DAC geometry.

PLAT971_ALERT_2_A Check Calcd Resid. Dens. 1.54Ang From Bi1 4.61 eA-3

Author Response: Multigrain sample in a DAC. Strong electronic contrast between Bi and N and limited completeness.

PLAT971_ALERT_2_A Check Calcd Resid. Dens. 0.79Ang From N1 4.43 eA-3

Author Response: Multigrain sample in a DAC. Strong electronic contrast between Bi and N and limited completeness.

PLAT971_ALERT_2_A Check Calcd Resid. Dens. 1.46Ang From N2 4.17 eA-3

Author Response: Multigrain sample in a DAC. Strong electronic contrast between Bi and N and limited completeness.

PLAT971_ALERT_2_A Check Calcd Resid. Dens. 0.88Ang From Bi2 3.99 eA-3

Author Response: Multigrain sample in a DAC. Strong electronic contrast between Bi and N and limited completeness.

PLAT971_ALERT_2_A Check Calcd Resid. Dens. 1.23Ang From N1 3.88 eA-3

Author Response: Multigrain sample in a DAC. Strong electronic contrast between Bi and N and limited completeness.

PLAT971_ALERT_2_A Check Calcd Resid. Dens. 1.38Ang From N1 3.82 eA-3

Author Response: Multigrain sample in a DAC. Strong electronic contrast between Bi and N and limited completeness.

PLAT971_ALERT_2_A Check Calcd Resid. Dens. 1.17Ang From N1 3.76 eA-3

Author Response: Multigrain sample in a DAC. Strong electronic contrast between Bi and N and limited completeness.

```
PLAT971_ALERT_2_A Check Calcd Resid. Dens.  1.01Ang From Bi1            3.74 eA-3
```

Author Response: Multigrain sample in a DAC. Strong electronic contrast between Bi and N and limited completeness.

```
PLAT971_ALERT_2_A Check Calcd Resid. Dens.  1.50Ang From N2             3.71 eA-3
```

Author Response: Multigrain sample in a DAC. Strong electronic contrast between Bi and N and limited completeness.

```
PLAT971_ALERT_2_A Check Calcd Resid. Dens.  1.03Ang From N2             3.69 eA-3
```

Author Response: Multigrain sample in a DAC. Strong electronic contrast between Bi and N and limited completeness.

```
PLAT971_ALERT_2_A Check Calcd Resid. Dens.  1.25Ang From N2             3.68 eA-3
```

Author Response: Multigrain sample in a DAC. Strong electronic contrast between Bi and N and limited completeness.

```
PLAT971_ALERT_2_A Check Calcd Resid. Dens.  0.91Ang From Bi1            3.60 eA-3
```

Author Response: Multigrain sample in a DAC. Strong electronic contrast between Bi and N and limited completeness.

```
PLAT971_ALERT_2_A Check Calcd Resid. Dens.  1.47Ang From N1             3.56 eA-3
```

Author Response: Multigrain sample in a DAC. Strong electronic contrast between Bi and N and limited completeness.

```
PLAT971_ALERT_2_A Check Calcd Resid. Dens.  1.57Ang From Bi2            3.55 eA-3
```

Author Response: Multigrain sample in a DAC. Strong electronic contrast between Bi and N and limited completeness.

```
PLAT972_ALERT_2_A Check Calcd Resid. Dens.  1.24Ang From Bi1           -5.36 eA-3
```

Author Response: Multigrain sample in a DAC. Strong electronic contrast between Bi and N and limited completeness.

PLAT972_ALERT_2_A Check Calcd Resid. Dens. 0.60Ang From Bi1 -4.74 eA-3

Author Response: Multigrain sample in a DAC. Strong electronic contrast between Bi and N and limited completeness.

PLAT972_ALERT_2_A Check Calcd Resid. Dens. 1.12Ang From N1 -4.35 eA-3

Author Response: Multigrain sample in a DAC. Strong electronic contrast between Bi and N and limited completeness.

PLAT972_ALERT_2_A Check Calcd Resid. Dens. 1.22Ang From Bi2 -4.30 eA-3

Author Response: Multigrain sample in a DAC. Strong electronic contrast between Bi and N and limited completeness.

PLAT972_ALERT_2_A Check Calcd Resid. Dens. 0.74Ang From Bi2 -4.30 eA-3

Author Response: Multigrain sample in a DAC. Strong electronic contrast between Bi and N and limited completeness.

PLAT972_ALERT_2_A Check Calcd Resid. Dens. 0.73Ang From Bi1 -4.27 eA-3

Author Response: Multigrain sample in a DAC. Strong electronic contrast between Bi and N and limited completeness.

PLAT972_ALERT_2_A Check Calcd Resid. Dens. 0.95Ang From N2 -4.20 eA-3

Author Response: Multigrain sample in a DAC. Strong electronic contrast between Bi and N and limited completeness.

PLAT972_ALERT_2_A Check Calcd Resid. Dens. 0.56Ang From Bi1 -3.99 eA-3

Author Response: Multigrain sample in a DAC. Strong electronic contrast between Bi and N and limited completeness.

PLAT972_ALERT_2_A Check Calcd Resid. Dens. 0.53Ang From Bi1 -3.96 eA-3

Author Response: Multigrain sample in a DAC. Strong electronic contrast between Bi and N and limited completeness.

PLAT972_ALERT_2_A Check Calcd Resid. Dens. 0.58Ang From Bi2 -3.94 eA-3

Author Response: Multigrain sample in a DAC. Strong electronic contrast between Bi and N and limited completeness.

```
PLAT972_ALERT_2_A Check Calcd Resid. Dens.  1.58Ang From N1            -3.62 eA-3
```

Author Response: Multigrain sample in a DAC. Strong electronic contrast between Bi and N and limited completeness.

```
PLAT972_ALERT_2_A Check Calcd Resid. Dens.  0.78Ang From N2            -3.61 eA-3
```

Author Response: Multigrain sample in a DAC. Strong electronic contrast between Bi and N and limited completeness.

```
PLAT972_ALERT_2_A Check Calcd Resid. Dens.  1.27Ang From N1            -3.51 eA-3
```

Author Response: Multigrain sample in a DAC. Strong electronic contrast between Bi and N and limited completeness.

```
PLAT973_ALERT_2_A Check Calcd Positive Resid. Density on       Bi1       5.04 eA-3
```

Author Response: Multigrain sample in a DAC. Strong electronic contrast between Bi and N and limited completeness.

```
PLAT973_ALERT_2_A Check Calcd Positive Resid. Density on       Bi2       3.87 eA-3
```

Author Response: Multigrain sample in a DAC. Strong electronic contrast between Bi and N and limited completeness.

```
PLAT975_ALERT_2_A Check Calcd Resid. Dens.  0.79Ang From N1      .       4.43 eA-3
```

Author Response: Multigrain sample in a DAC. Strong electronic contrast between Bi and N and limited completeness.

```
PLAT976_ALERT_2_A Check Calcd Resid. Dens.  0.95Ang From N2      .      -4.20 eA-3
```

Author Response: Multigrain sample in a DAC. Strong electronic contrast between Bi and N and limited completeness.

Alert level B

```
PLAT911_ALERT_3_B Missing FCF Refl Between Thmin & STh/L=    0.600        218 Report
```

Author Response: Multigrain sample in a diamond anvil cell (DAC). Aperture is limited by the DAC geometry.

```
PLAT921_ALERT_1_B R1  in the CIF and FCF Differ by ..............     0.0068 Check
```

Author Response: Both files were prepared using the same program - JANA2006. Should be an internal program issue.

```
PLAT926_ALERT_1_B Reported and Calculated   R1 Differ by .........     0.0066 Check
```

Author Response: Both files were prepared using the same program - JANA2006. Should be an internal program issue.

```
PLAT971_ALERT_2_B Check Calcd Resid. Dens.  1.02Ang From Bi1            3.50 eA-3
```

Author Response: Multigrain sample in a DAC. Strong electronic contrast between Bi and N and limited completeness.

```
PLAT971_ALERT_2_B Check Calcd Resid. Dens.  1.44Ang From N1             3.46 eA-3
```

Author Response: Multigrain sample in a DAC. Strong electronic contrast between Bi and N and limited completeness.

```
PLAT971_ALERT_2_B Check Calcd Resid. Dens.  1.33Ang From N2             3.42 eA-3
```

Author Response: Multigrain sample in a DAC. Strong electronic contrast between Bi and N and limited completeness.

```
PLAT971_ALERT_2_B Check Calcd Resid. Dens.  0.91Ang From N2             3.39 eA-3
```

Author Response: Multigrain sample in a DAC. Strong electronic contrast between Bi and N and limited completeness.

```
PLAT971_ALERT_2_B Check Calcd Resid. Dens.  1.40Ang From Bi1            3.38 eA-3
```

Author Response: Multigrain sample in a DAC. Strong electronic contrast between Bi and N and limited completeness.

```
PLAT971_ALERT_2_B Check Calcd Resid. Dens.  0.92Ang From Bi2            3.35 eA-3
```

Author Response: Multigrain sample in a DAC. Strong electronic contrast between Bi and N and limited completeness.

PLAT971_ALERT_2_B Check Calcd Resid. Dens. 1.48Ang From N1 3.29 eA-3

Author Response: Multigrain sample in a DAC. Strong electronic contrast between Bi and N and limited completeness.

PLAT971_ALERT_2_B Check Calcd Resid. Dens. 1.26Ang From Bi1 3.27 eA-3

Author Response: Multigrain sample in a DAC. Strong electronic contrast between Bi and N and limited completeness.

PLAT971_ALERT_2_B Check Calcd Resid. Dens. 1.33Ang From N1 3.26 eA-3

Author Response: Multigrain sample in a DAC. Strong electronic contrast between Bi and N and limited completeness.

PLAT972_ALERT_2_B Check Calcd Resid. Dens. 1.00Ang From Bi2 -3.50 eA-3

Author Response: Multigrain sample in a DAC. Strong electronic contrast between Bi and N and limited completeness.

PLAT972_ALERT_2_B Check Calcd Resid. Dens. 1.45Ang From N2 -3.50 eA-3

Author Response: Multigrain sample in a DAC. Strong electronic contrast between Bi and N and limited completeness.

PLAT972_ALERT_2_B Check Calcd Resid. Dens. 1.61Ang From N1 -3.47 eA-3

Author Response: Multigrain sample in a DAC. Strong electronic contrast between Bi and N and limited completeness.

PLAT972_ALERT_2_B Check Calcd Resid. Dens. 1.04Ang From N1 -3.29 eA-3

Author Response: Multigrain sample in a DAC. Strong electronic contrast between Bi and N and limited completeness.

PLAT972_ALERT_2_B Check Calcd Resid. Dens. 0.76Ang From Bi1 -3.29 eA-3

Author Response: Multigrain sample in a DAC. Strong electronic contrast between Bi and N and limited completeness.

PLAT972_ALERT_2_B Check Calcd Resid. Dens. 1.61Ang From Bi2 -3.28 eA-3

Author Response: Multigrain sample in a DAC. Strong electronic contrast between Bi and N and limited completeness.

PLAT972_ALERT_2_B Check Calcd Resid. Dens. 1.05Ang From Bi1   -3.25 eA-3

Author Response: Multigrain sample in a DAC. Strong electronic contrast between Bi and N and limited completeness.

PLAT972_ALERT_2_B Check Calcd Resid. Dens. 1.29Ang From N2   -3.23 eA-3

Author Response: Multigrain sample in a DAC. Strong electronic contrast between Bi and N and limited completeness.

PLAT972_ALERT_2_B Check Calcd Resid. Dens. 0.39Ang From Bi2   -3.21 eA-3

Author Response: Multigrain sample in a DAC. Strong electronic contrast between Bi and N and limited completeness.

PLAT972_ALERT_2_B Check Calcd Resid. Dens. 0.40Ang From N2   -3.17 eA-3

Author Response: Multigrain sample in a DAC. Strong electronic contrast between Bi and N and limited completeness.

PLAT972_ALERT_2_B Check Calcd Resid. Dens. 0.99Ang From Bi2   -3.15 eA-3

Author Response: Multigrain sample in a DAC. Strong electronic contrast between Bi and N and limited completeness.

PLAT972_ALERT_2_B Check Calcd Resid. Dens. 0.65Ang From Bi2   -3.07 eA-3

Author Response: Multigrain sample in a DAC. Strong electronic contrast between Bi and N and limited completeness.

Alert level C

```
PLAT041_ALERT_1_C Calc. and Reported SumFormula    Strings  Differ     Please Check
PLAT051_ALERT_1_C Mu(calc) and Mu(CIF) Ratio Differs from 1.0 by .       1.24 %
PLAT053_ALERT_1_C Minimum Crystal Dimension Missing (or Error) ...     Please Check
PLAT054_ALERT_1_C Medium  Crystal Dimension Missing (or Error) ...     Please Check
PLAT055_ALERT_1_C Maximum Crystal Dimension Missing (or Error) ...     Please Check
PLAT127_ALERT_1_C Implicit Hall Symbol  Inconsistent with Explicit -P -2xab;- Check
PLAT148_ALERT_3_C s.u. on the      c    - Axis is (Too) Large ....      0.013 Ang.
PLAT906_ALERT_3_C Large K Value in the Analysis of Variance ......      6.939 Check
PLAT920_ALERT_1_C Theta(Max)  in CIF and FCF Differ by ..........        0.42 Degree
PLAT992_ALERT_5_C Repd & Actual _reflns_number_gt Values Differ by         39 Check
```

Alert level G

```
ABSMU01_ALERT_1_G  Calculation of _exptl_absorpt_correction_mu
                not performed for this radiation type.
PLAT004_ALERT_5_G Polymeric Structure Found with Maximum Dimension          3 Info
PLAT005_ALERT_5_G No Embedded Refinement Details Found  in the CIF     Please Do !
PLAT045_ALERT_1_G Calculated and Reported Z Differ by a Factor ...      0.500 Check
PLAT092_ALERT_4_G Check: Wavelength Given is not Cu,Ga,Mo,Ag,In Ka    0.29040 Ang.
PLAT199_ALERT_1_G Reported _cell_measurement_temperature ..... (K)        293 Check
PLAT200_ALERT_1_G Reported   _diffrn_ambient_temperature ..... (K)        293 Check
PLAT794_ALERT_5_G Tentative Bond Valency for Bi1       (III)     .       2.36 Info
PLAT794_ALERT_5_G Tentative Bond Valency for Bi2       (III)     .       2.32 Info
PLAT883_ALERT_1_G No Info/Value for _atom_sites_solution_primary .     Please Do !
PLAT910_ALERT_3_G Missing # of FCF Reflection(s) Below Theta(Min).          3 Note
PLAT912_ALERT_4_G Missing # of FCF Reflections Above STh/L=  0.600        358 Note
PLAT950_ALERT_5_G Calculated (ThMax) and CIF-Reported Hmax Differ           3 Units
PLAT951_ALERT_5_G Calculated (ThMax) and CIF-Reported Kmax Differ           5 Units
PLAT952_ALERT_5_G Calculated (ThMax) and CIF-Reported Lmax Differ.         10 Units
PLAT956_ALERT_1_G Calculated (ThMax) and Actual (FCF) Hmax Differ           3 Units
PLAT957_ALERT_1_G Calculated (ThMax) and Actual (FCF) Kmax Differ           5 Units
PLAT958_ALERT_1_G Calculated (ThMax) and Actual (FCF) Lmax Differ.         10 Units
PLAT961_ALERT_5_G Dataset Contains no Negative Intensities .......     Please Check
PLAT984_ALERT_1_G The Bi-f'=   -0.8743 Deviates from the B&C-Value    -0.8048 Check
PLAT985_ALERT_1_G The Bi-f"=    2.4721 Deviates from the B&C-Value     2.4496 Check
```

---

32 **ALERT level A** = Most likely a serious problem - resolve or explain
24 **ALERT level B** = A potentially serious problem, consider carefully
10 **ALERT level C** = Check. Ensure it is not caused by an omission or oversight
21 **ALERT level G** = General information/check it is not something unexpected

19 ALERT type 1 CIF construction/syntax error, inconsistent or missing data
52 ALERT type 2 Indicator that the structure model may be wrong or deficient
5 ALERT type 3 Indicator that the structure quality may be low
2 ALERT type 4 Improvement, methodology, query or suggestion
9 ALERT type 5 Informative message, check

It is advisable to attempt to resolve as many as possible of the alerts in all categories. Often the minor alerts point to easily fixed oversights, errors and omissions in your CIF or refinement strategy, so attention to these fine details can be worthwhile. In order to resolve some of the more serious problems it may be necessary to carry out additional measurements or structure refinements. However, the purpose of your study may justify the reported deviations and the more serious of these should normally be commented upon in the discussion or experimental section of a paper or in the "special_details" fields of the CIF. checkCIF was carefully designed to identify outliers and unusual parameters, but every test has its limitations and alerts that are not important in a particular case may appear. Conversely, the absence of alerts does not guarantee there are no aspects of the results needing attention. It is up to the individual to critically assess their own results and, if necessary, seek expert advice.

Publication of your CIF in IUCr journals

A basic structural check has been run on your CIF. These basic checks will be run on all CIFs submitted for publication in IUCr journals (*Acta Crystallographica*, *Journal of Applied Crystallography*, *Journal of Synchrotron Radiation*); however, if you intend to submit to *Acta Crystallographica Section C* or *E* or *IUCrData*, you should make sure that full publication checks are run on the final version of your CIF prior to submission.

Publication of your CIF in other journals

Please refer to the *Notes for Authors* of the relevant journal for any special instructions relating to CIF submission.

PLATON version of 10/05/2023; check.def file version of 10/05/2023

Datablock I - ellipsoid plot

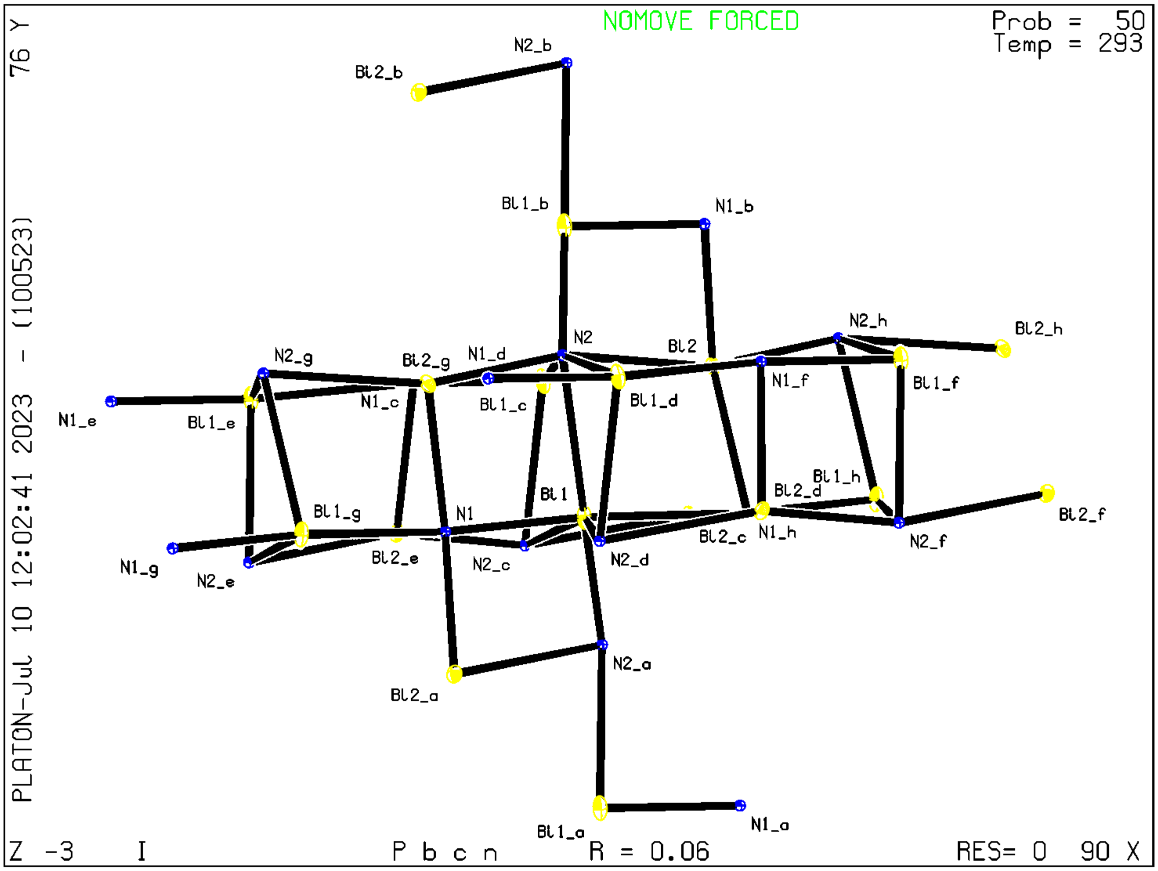

Supplement: Supplementary file 3 [file DataSheet4.PDF]
