# Supplementary material for: High-pressure reactions between the pnictogens: the rediscovery of BiN
Source: Front Chem. 2023 Oct 12;11:1257942. doi: 10.3389/fchem.2023.1257942 (PMC10602720; doi:10.3389/fchem.2023.1257942)

checkCIF/PLATON report

Structure factors have been supplied for datablock(s) I

THIS REPORT IS FOR GUIDANCE ONLY. IF USED AS PART OF A REVIEW PROCEDURE FOR PUBLICATION, IT SHOULD NOT REPLACE THE EXPERTISE OF AN EXPERIENCED CRYSTALLOGRAPHIC REFEREE.

No syntax errors found. CIF dictionary Interpreting this report

Datablock: I

```
Bond precision:   Bi- N = 0.0573 A         Wavelength=0.29040

Cell:               a=4.8613(19)       b=9.394(3)      c=9.249(15)
                    alpha=90           beta=90         gamma=90
Temperature:        293 K

                 Calculated                   Reported
Volume           422.4(7)                     422.4(7)
Space group      P b c n                      P b c n
Hall group       -P 2n 2ab                    -P -2xab;-2
Moiety formula   Bi2 N2                       ?
Sum formula      Bi2 N2                       Bi1 N1
Mr               445.98                       223.00
Dx,g cm-3        14.030                       14.026
Z                8                            16
Mu (mm-1)        16.410                       16.618
F000             1440.0                       1440.0
F000'            1426.84
h,k,lmax         10,19,19                     7,16,10
Nref             2036                         413
Tmin,Tmax                                     0.720,1.000
Tmin'

Correction method= # Reported T Limits: Tmin=0.720 Tmax=1.000
AbsCorr = MULTI-SCAN

Data completeness= 0.203           Theta(max)= 17.710

R(reflections)= 0.0755( 205)                         wR2(reflections)=
                                                     0.1561( 413)
S = 0.890                  Npar= 26
```

The following ALERTS were generated. Each ALERT has the format
test-name_ALERT_alert-type_alert-level.
Click on the hyperlinks for more details of the test.

Alert level A

PLAT029_ALERT_3_A _diffrn_measured_fraction_theta_full value Low . 0.360 Why?

Author Response: Multigrain sample in a diamond anvil cell (DAC). Aperture is limited by the DAC geometry. Weakly scattering sample.

PLAT971_ALERT_2_A Check Calcd Resid. Dens. 0.99Ang From Bi2 4.10 eA-3

Author Response: Multigrain sample in a DAC. Strong electronic contrast between Bi and N and limited completeness. Weakly scattering sample.

PLAT971_ALERT_2_A Check Calcd Resid. Dens. 1.22Ang From Bi2 3.68 eA-3

Author Response: Multigrain sample in a DAC. Strong electronic contrast between Bi and N and limited completeness. Weakly scattering sample.

PLAT971_ALERT_2_A Check Calcd Resid. Dens. 0.80Ang From Bi1 3.67 eA-3

Author Response: Multigrain sample in a DAC. Strong electronic contrast between Bi and N and limited completeness. Weakly scattering sample.

PLAT971_ALERT_2_A Check Calcd Resid. Dens. 0.82Ang From N1 3.66 eA-3

Author Response: Multigrain sample in a DAC. Strong electronic contrast between Bi and N and limited completeness. Weakly scattering sample.

PLAT971_ALERT_2_A Check Calcd Resid. Dens. 0.63Ang From N2 3.58 eA-3

Author Response: Multigrain sample in a DAC. Strong electronic contrast between Bi and N and limited completeness. Weakly scattering sample.

PLAT972_ALERT_2_A Check Calcd Resid. Dens. 0.73Ang From Bi1 -5.01 eA-3

Author Response: Multigrain sample in a DAC. Strong electronic contrast between Bi and N and limited completeness. Weakly scattering sample.

PLAT972_ALERT_2_A Check Calcd Resid. Dens. 1.18Ang From N2 -4.12 eA-3

Author Response: Multigrain sample in a DAC. Strong electronic contrast between Bi and N and limited completeness. Weakly scattering sample.

```
PLAT972_ALERT_2_A Check Calcd Resid. Dens.  1.19Ang From Bi1           -4.00 eA-3
```

Author Response: Multigrain sample in a DAC. Strong electronic contrast between Bi and N and limited completeness. Weakly scattering sample.

```
PLAT972_ALERT_2_A Check Calcd Resid. Dens.  0.63Ang From Bi2           -3.79 eA-3
```

Author Response: Multigrain sample in a DAC. Strong electronic contrast between Bi and N and limited completeness. Weakly scattering sample.

```
PLAT972_ALERT_2_A Check Calcd Resid. Dens.  0.87Ang From N1            -3.72 eA-3
```

Author Response: Multigrain sample in a DAC. Strong electronic contrast between Bi and N and limited completeness. Weakly scattering sample.

```
PLAT972_ALERT_2_A Check Calcd Resid. Dens.  0.66Ang From Bi1           -3.64 eA-3
```

Author Response: Multigrain sample in a DAC. Strong electronic contrast between Bi and N and limited completeness. Weakly scattering sample.

```
PLAT972_ALERT_2_A Check Calcd Resid. Dens.  0.98Ang From Bi2           -3.58 eA-3
```

Author Response: Multigrain sample in a DAC. Strong electronic contrast between Bi and N and limited completeness. Weakly scattering sample.

```
PLAT972_ALERT_2_A Check Calcd Resid. Dens.  1.08Ang From N1            -3.51 eA-3
```

Author Response: Multigrain sample in a DAC. Strong electronic contrast between Bi and N and limited completeness. Weakly scattering sample.

```
PLAT975_ALERT_2_A Check Calcd Resid. Dens.  0.63Ang From N2      .      3.58 eA-3
```

Author Response: Multigrain sample in a DAC. Strong electronic contrast between Bi and N and limited completeness. Weakly scattering sample.

```
PLAT975_ALERT_2_A Check Calcd Resid. Dens.  0.95Ang From N2      .      2.84 eA-3
```

Author Response: Multigrain sample in a DAC. Strong electronic contrast between Bi and N and limited completeness. Weakly scattering sample.

```
PLAT976_ALERT_2_A Check Calcd Resid. Dens.  0.87Ang From N1      .      -3.72 eA-3
```

Author Response: Multigrain sample in a DAC. Strong electronic contrast between Bi and N and limited completeness. Weakly scattering sample.

```
PLAT976_ALERT_2_A Check Calcd Resid. Dens.  1.01Ang From N2      .      -3.04 eA-3
```

Author Response: Multigrain sample in a DAC. Strong electronic contrast between Bi and N and limited completeness. Weakly scattering sample.

```
PLAT976_ALERT_2_A Check Calcd Resid. Dens.  0.71Ang From N2      .      -2.75 eA-3
```

Author Response: Multigrain sample in a DAC. Strong electronic contrast between Bi and N and limited completeness. Weakly scattering sample.

Alert level B

```
PLAT911_ALERT_3_B Missing FCF Refl Between Thmin & STh/L=    0.600        229 Report
```

Author Response: Multigrain sample in a DAC. Strong electronic contrast between Bi and N and limited completeness. Weakly scattering sample.

```
PLAT921_ALERT_1_B R1  in the CIF and FCF Differ by ..............     0.0101 Check
```

Author Response: Values were calculated by JANA2006, must be an internal bug.

```
PLAT926_ALERT_1_B Reported and Calculated   R1 Differ by .........     0.0105 Check
```

Author Response: Values were calculated by JANA2006, must be an internal bug.

```
PLAT971_ALERT_2_B Check Calcd Resid. Dens.  0.74Ang From N1             3.46 eA-3
```

Author Response: Multigrain sample in a DAC. Strong electronic contrast between Bi and N and limited completeness. Weakly scattering sample.

```
PLAT971_ALERT_2_B Check Calcd Resid. Dens.  1.03Ang From Bi1            3.38 eA-3
```

Author Response: Multigrain sample in a DAC. Strong electronic contrast between Bi and N and limited completeness. Weakly scattering sample.

```
PLAT971_ALERT_2_B Check Calcd Resid. Dens.  0.20Ang From Bi1            3.29 eA-3
```

Author Response: Multigrain sample in a DAC. Strong electronic contrast between Bi and N and limited completeness. Weakly scattering sample.

```
PLAT971_ALERT_2_B Check Calcd Resid. Dens.  1.25Ang From N2            3.24 eA-3
```

Author Response: Multigrain sample in a DAC. Strong electronic contrast between Bi and N and limited completeness. Weakly scattering sample.

```
PLAT971_ALERT_2_B Check Calcd Resid. Dens.  1.38Ang From N1            3.22 eA-3
```

Author Response: Multigrain sample in a DAC. Strong electronic contrast between Bi and N and limited completeness. Weakly scattering sample.

```
PLAT971_ALERT_2_B Check Calcd Resid. Dens.  1.46Ang From Bi1           3.13 eA-3
```

Author Response: Multigrain sample in a DAC. Strong electronic contrast between Bi and N and limited completeness. Weakly scattering sample.

```
PLAT971_ALERT_2_B Check Calcd Resid. Dens.  1.13Ang From Bi1           3.11 eA-3
```

Author Response: Multigrain sample in a DAC. Strong electronic contrast between Bi and N and limited completeness. Weakly scattering sample.

```
PLAT971_ALERT_2_B Check Calcd Resid. Dens.  1.50Ang From N1            3.02 eA-3
```

Author Response: Multigrain sample in a DAC. Strong electronic contrast between Bi and N and limited completeness. Weakly scattering sample.

```
PLAT971_ALERT_2_B Check Calcd Resid. Dens.  1.30Ang From Bi1           3.02 eA-3
```

Author Response: Multigrain sample in a DAC. Strong electronic contrast between Bi and N and limited completeness. Weakly scattering sample.

```
PLAT971_ALERT_2_B Check Calcd Resid. Dens.  1.36Ang From Bi2           2.88 eA-3
```

Author Response: Multigrain sample in a DAC. Strong electronic contrast between Bi and N and limited completeness. Weakly scattering sample.

```
PLAT971_ALERT_2_B Check Calcd Resid. Dens.  1.45Ang From N2            2.88 eA-3
```

Author Response: Multigrain sample in a DAC. Strong electronic contrast between Bi and N and limited completeness. Weakly scattering sample.

PLAT971_ALERT_2_B Check Calcd Resid. Dens. 1.54Ang From N1 2.86 eA-3

Author Response: Multigrain sample in a DAC. Strong electronic contrast between Bi and N and limited completeness. Weakly scattering sample.

PLAT971_ALERT_2_B Check Calcd Resid. Dens. 0.91Ang From Bi1 2.85 eA-3

Author Response: Multigrain sample in a DAC. Strong electronic contrast between Bi and N and limited completeness. Weakly scattering sample.

PLAT971_ALERT_2_B Check Calcd Resid. Dens. 0.95Ang From N2 2.84 eA-3

Author Response: Multigrain sample in a DAC. Strong electronic contrast between Bi and N and limited completeness. Weakly scattering sample.

PLAT971_ALERT_2_B Check Calcd Resid. Dens. 0.99Ang From Bi1 2.80 eA-3

Author Response: Multigrain sample in a DAC. Strong electronic contrast between Bi and N and limited completeness. Weakly scattering sample.

PLAT971_ALERT_2_B Check Calcd Resid. Dens. 1.29Ang From Bi2 2.78 eA-3

Author Response: Multigrain sample in a DAC. Strong electronic contrast between Bi and N and limited completeness. Weakly scattering sample.

PLAT971_ALERT_2_B Check Calcd Resid. Dens. 1.31Ang From N1 2.77 eA-3

Author Response: Multigrain sample in a DAC. Strong electronic contrast between Bi and N and limited completeness. Weakly scattering sample.

PLAT971_ALERT_2_B Check Calcd Resid. Dens. 1.44Ang From Bi2 2.77 eA-3

Author Response: Multigrain sample in a DAC. Strong electronic contrast between Bi and N and limited completeness. Weakly scattering sample.

PLAT971_ALERT_2_B Check Calcd Resid. Dens. 1.40Ang From Bi2 2.76 eA-3

Author Response: Multigrain sample in a DAC. Strong electronic contrast between Bi and N and limited completeness. Weakly scattering sample.

PLAT971_ALERT_2_B Check Calcd Resid. Dens. 1.51Ang From Bi2 2.76 eA-3

Author Response: Multigrain sample in a DAC. Strong electronic contrast between Bi and N and limited completeness. Weakly scattering sample.

```
PLAT972_ALERT_2_B Check Calcd Resid. Dens.  1.05Ang From N1            -3.34 eA-3
```

Author Response: Multigrain sample in a DAC. Strong electronic contrast between Bi and N and limited completeness. Weakly scattering sample.

```
PLAT972_ALERT_2_B Check Calcd Resid. Dens.  1.42Ang From Bi1           -3.29 eA-3
```

Author Response: Multigrain sample in a DAC. Strong electronic contrast between Bi and N and limited completeness. Weakly scattering sample.

```
PLAT972_ALERT_2_B Check Calcd Resid. Dens.  0.45Ang From N1            -3.26 eA-3
```

Author Response: Multigrain sample in a DAC. Strong electronic contrast between Bi and N and limited completeness. Weakly scattering sample.

```
PLAT972_ALERT_2_B Check Calcd Resid. Dens.  0.66Ang From N2            -3.25 eA-3
```

Author Response: Multigrain sample in a DAC. Strong electronic contrast between Bi and N and limited completeness. Weakly scattering sample.

```
PLAT972_ALERT_2_B Check Calcd Resid. Dens.  1.53Ang From N1            -3.22 eA-3
```

Author Response: Multigrain sample in a DAC. Strong electronic contrast between Bi and N and limited completeness. Weakly scattering sample.

```
PLAT972_ALERT_2_B Check Calcd Resid. Dens.  1.26Ang From Bi2           -3.22 eA-3
```

Author Response: Multigrain sample in a DAC. Strong electronic contrast between Bi and N and limited completeness. Weakly scattering sample.

```
PLAT972_ALERT_2_B Check Calcd Resid. Dens.  1.32Ang From Bi2           -3.18 eA-3
```

Author Response: Multigrain sample in a DAC. Strong electronic contrast between Bi and N and limited completeness. Weakly scattering sample.

```
PLAT972_ALERT_2_B Check Calcd Resid. Dens.  1.32Ang From Bi2           -3.18 eA-3
```

Author Response: Multigrain sample in a DAC. Strong electronic contrast between Bi and N and limited completeness. Weakly scattering sample.

```
PLAT972_ALERT_2_B Check Calcd Resid. Dens.  1.01Ang From N2            -3.04 eA-3
```

Author Response: Multigrain sample in a DAC. Strong electronic contrast between Bi and N and limited completeness. Weakly scattering sample.

```
PLAT972_ALERT_2_B Check Calcd Resid. Dens.  1.73Ang From Bi2           -3.02 eA-3
```

Author Response: Multigrain sample in a DAC. Strong electronic contrast between Bi and N and limited completeness. Weakly scattering sample.

```
PLAT972_ALERT_2_B Check Calcd Resid. Dens.  1.07Ang From N1            -2.97 eA-3
```

Author Response: Multigrain sample in a DAC. Strong electronic contrast between Bi and N and limited completeness. Weakly scattering sample.

```
PLAT972_ALERT_2_B Check Calcd Resid. Dens.  1.12Ang From N1            -2.95 eA-3
```

Author Response: Multigrain sample in a DAC. Strong electronic contrast between Bi and N and limited completeness. Weakly scattering sample.

```
PLAT972_ALERT_2_B Check Calcd Resid. Dens.  0.85Ang From Bi1           -2.93 eA-3
```

Author Response: Multigrain sample in a DAC. Strong electronic contrast between Bi and N and limited completeness. Weakly scattering sample.

```
PLAT972_ALERT_2_B Check Calcd Resid. Dens.  1.16Ang From N2            -2.91 eA-3
```

Author Response: Multigrain sample in a DAC. Strong electronic contrast between Bi and N and limited completeness. Weakly scattering sample.

```
PLAT972_ALERT_2_B Check Calcd Resid. Dens.  1.54Ang From N1            -2.87 eA-3
```

Author Response: Multigrain sample in a DAC. Strong electronic contrast between Bi and N and limited completeness. Weakly scattering sample.

```
PLAT972_ALERT_2_B Check Calcd Resid. Dens.  1.08Ang From Bi1           -2.84 eA-3
```

Author Response: Multigrain sample in a DAC. Strong electronic contrast between Bi and N and limited completeness. Weakly scattering sample.

```
PLAT972_ALERT_2_B Check Calcd Resid. Dens.  0.71Ang From N2            -2.75 eA-3
```

Author Response: Multigrain sample in a DAC. Strong electronic contrast between Bi and N and limited completeness. Weakly scattering sample.

```
PLAT992_ALERT_5_B Repd & Actual _reflns_number_gt Values Differ by         53 Check
```

Author Response: Values were calculated by JANA2006, must be an internal bug.

Alert level C

```
PLAT041_ALERT_1_C Calc. and Reported SumFormula    Strings  Differ     Please Check
PLAT051_ALERT_1_C Mu(calc) and Mu(CIF) Ratio Differs from 1.0 by .       1.25 %
PLAT053_ALERT_1_C Minimum Crystal Dimension Missing (or Error) ...     Please Check
PLAT054_ALERT_1_C Medium  Crystal Dimension Missing (or Error) ...     Please Check
PLAT055_ALERT_1_C Maximum Crystal Dimension Missing (or Error) ...     Please Check
PLAT127_ALERT_1_C Implicit Hall Symbol  Inconsistent with Explicit -P -2xab;- Check
PLAT148_ALERT_3_C s.u. on the      c    - Axis is (Too) Large ....      0.015 Ang.
PLAT906_ALERT_3_C Large K Value in the Analysis of Variance ......      9.831 Check
PLAT913_ALERT_3_C Missing # of Very Strong Reflections in FCF ....          6 Note
PLAT925_ALERT_1_C The Reported and Calculated Rho(max) Differ by .       1.17 eA-3
```

Alert level G

```
ABSMU01_ALERT_1_G  Calculation of _exptl_absorpt_correction_mu
                not performed for this radiation type.
PLAT004_ALERT_5_G Polymeric Structure Found with Maximum Dimension          3 Info
PLAT005_ALERT_5_G No Embedded Refinement Details Found  in the CIF     Please Do !
PLAT045_ALERT_1_G Calculated and Reported Z Differ by a Factor ...      0.500 Check
PLAT092_ALERT_4_G Check: Wavelength Given is not Cu,Ga,Mo,Ag,In Ka    0.29040 Ang.
PLAT199_ALERT_1_G Reported _cell_measurement_temperature ..... (K)        293 Check
PLAT200_ALERT_1_G Reported   _diffrn_ambient_temperature ..... (K)        293 Check
PLAT794_ALERT_5_G Tentative Bond Valency for Bi1       (III)     .       2.34 Info
PLAT794_ALERT_5_G Tentative Bond Valency for Bi2       (III)     .       2.28 Info
PLAT883_ALERT_1_G No Info/Value for _atom_sites_solution_primary .     Please Do !
PLAT908_ALERT_2_G Max. Perc. Data with I > 2*s(I) per Res.Shell .      71.43% Note
PLAT910_ALERT_3_G Missing # of FCF Reflection(s) Below Theta(Min).          3 Note
PLAT912_ALERT_4_G Missing # of FCF Reflections Above STh/L=  0.600        481 Note
PLAT950_ALERT_5_G Calculated (ThMax) and CIF-Reported Hmax Differ           3 Units
PLAT951_ALERT_5_G Calculated (ThMax) and CIF-Reported Kmax Differ           3 Units
PLAT952_ALERT_5_G Calculated (ThMax) and CIF-Reported Lmax Differ.          9 Units
PLAT955_ALERT_1_G Reported (CIF) and Actual (FCF) Lmax Differ by .          1 Units
PLAT956_ALERT_1_G Calculated (ThMax) and Actual (FCF) Hmax Differ           3 Units
PLAT957_ALERT_1_G Calculated (ThMax) and Actual (FCF) Kmax Differ           3 Units
PLAT958_ALERT_1_G Calculated (ThMax) and Actual (FCF) Lmax Differ.         10 Units
PLAT961_ALERT_5_G Dataset Contains no Negative Intensities .......     Please Check
PLAT984_ALERT_1_G The Bi-f'=   -0.8743 Deviates from the B&C-Value    -0.8048 Check
PLAT985_ALERT_1_G The Bi-f"=    2.4721 Deviates from the B&C-Value     2.4496 Check
```

---

19 **ALERT level A** = Most likely a serious problem - resolve or explain
41 **ALERT level B** = A potentially serious problem, consider carefully
10 **ALERT level C** = Check. Ensure it is not caused by an omission or oversight
23 **ALERT level G** = General information/check it is not something unexpected

```
20 ALERT type 1 CIF construction/syntax error, inconsistent or missing data
56 ALERT type 2 Indicator that the structure model may be wrong or deficient
 6 ALERT type 3 Indicator that the structure quality may be low
 2 ALERT type 4 Improvement, methodology, query or suggestion
 9 ALERT type 5 Informative message, check
```

It is advisable to attempt to resolve as many as possible of the alerts in all categories. Often the minor alerts point to easily fixed oversights, errors and omissions in your CIF or refinement strategy, so attention to these fine details can be worthwhile. In order to resolve some of the more serious problems it may be necessary to carry out additional measurements or structure refinements. However, the purpose of your study may justify the reported deviations and the more serious of these should normally be commented upon in the discussion or experimental section of a paper or in the "special_details" fields of the CIF. checkCIF was carefully designed to identify outliers and unusual parameters, but every test has its limitations and alerts that are not important in a particular case may appear. Conversely, the absence of alerts does not guarantee there are no aspects of the results needing attention. It is up to the individual to critically assess their own results and, if necessary, seek expert advice.

Publication of your CIF in IUCr journals

A basic structural check has been run on your CIF. These basic checks will be run on all CIFs submitted for publication in IUCr journals (*Acta Crystallographica*, *Journal of Applied Crystallography*, *Journal of Synchrotron Radiation*); however, if you intend to submit to *Acta Crystallographica Section C* or *E* or *IUCrData*, you should make sure that full publication checks are run on the final version of your CIF prior to submission.

Publication of your CIF in other journals

Please refer to the *Notes for Authors* of the relevant journal for any special instructions relating to CIF submission.

PLATON version of 10/05/2023; check.def file version of 10/05/2023

Datablock I - ellipsoid plot

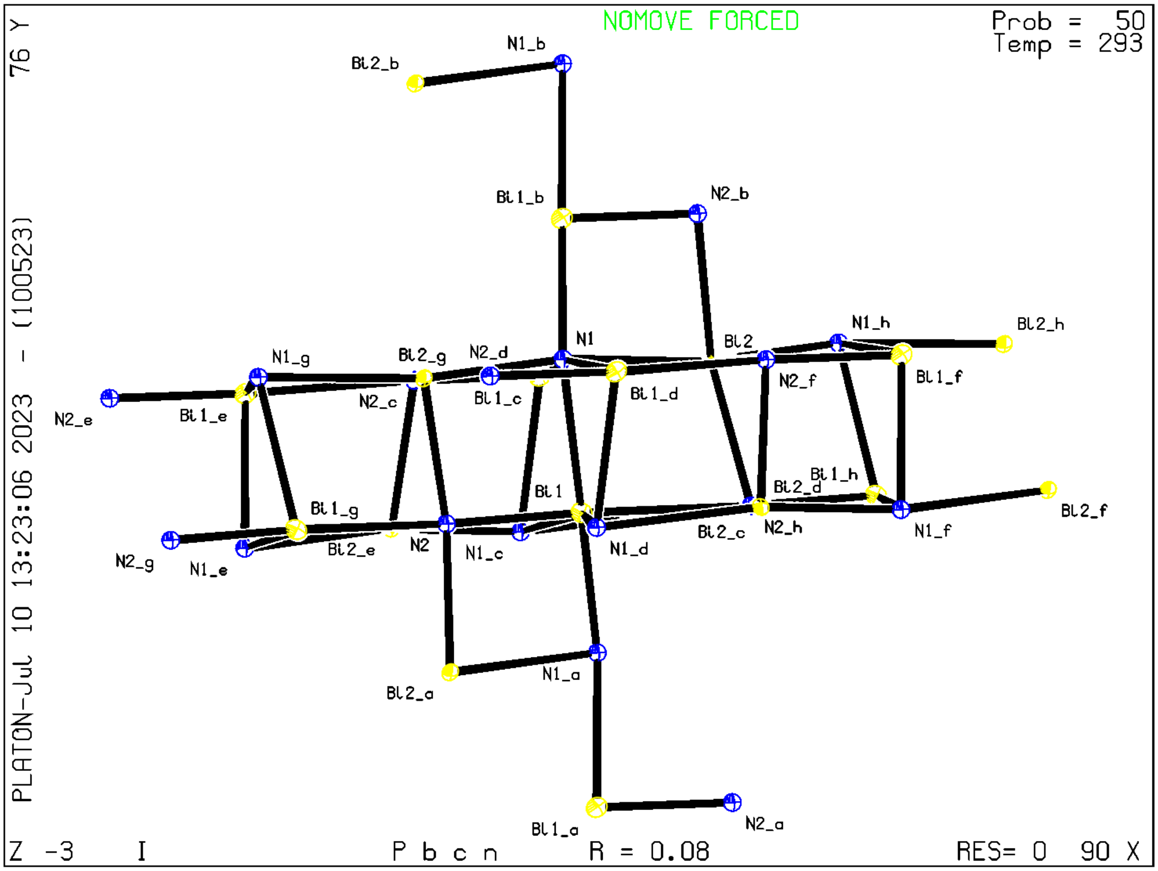

Supplement: Supplementary file 5 [file DataSheet9.PDF]
