# Supplementary material for: High-pressure reactions between the pnictogens: the rediscovery of BiN
Source: Front Chem. 2023 Oct 12;11:1257942. doi: 10.3389/fchem.2023.1257942 (PMC10602720; doi:10.3389/fchem.2023.1257942)

checkCIF/PLATON report

Structure factors have been supplied for datablock(s) I

THIS REPORT IS FOR GUIDANCE ONLY. IF USED AS PART OF A REVIEW PROCEDURE FOR PUBLICATION, IT SHOULD NOT REPLACE THE EXPERTISE OF AN EXPERIENCED CRYSTALLOGRAPHIC REFEREE.

No syntax errors found. CIF dictionary Interpreting this report

Datablock: I

```
Bond precision:   Bi- N = 0.0150 A         Wavelength=0.48390

Cell:               a=5.0002(7)       b=9.556(4)      c=9.3468(12)
                    alpha=90          beta=90         gamma=90
Temperature:        293 K

                 Calculated                    Reported
Volume           446.6(2)                      446.61(18)
Space group      P b c n                       P b c n
Hall group       -P 2n 2ab                     -P -2xab;-2
Moiety formula   Bi2 N2                        ?
Sum formula      Bi2 N2                        Bi1 N1
Mr               445.98                        223.00
Dx,g cm-3        13.266                        13.266
Z                8                             16
Mu (mm-1)        58.352                        58.629
F000             1440.0                        1440.0
F000'            1430.38
h,k,lmax         6,12,11                       5,7,10
Nref             467                           146
Tmin,Tmax                                      0.788,0.794
Tmin'

Correction method= # Reported T Limits: Tmin=0.788 Tmax=0.794
AbsCorr = SPHERE

Data completeness= 0.313           Theta(max)= 17.720

R(reflections)= 0.0328( 123)                         wR2(reflections)=
                                                     0.0788( 146)
S = 2.810                   Npar= 27
```

The following ALERTS were generated. Each ALERT has the format
test-name_ALERT_alert-type_alert-level.
Click on the hyperlinks for more details of the test.

Alert level A

```
PLAT027_ALERT_3_A _diffrn_reflns_theta_full value (too) Low ......       7.99 Degree
```

Author Response: Multigrain sample in a diamond anvil cell (DAC). Aperture is limited by the DAC geometry.

```
PLAT029_ALERT_3_A _diffrn_measured_fraction_theta_full value Low .      0.660 Why?
```

Author Response: Multigrain sample in a diamond anvil cell (DAC). Aperture is limited by the DAC geometry.

```
PLAT088_ALERT_3_A Poor Data / Parameter Ratio ....................       5.41 Note
```

Author Response: Multigrain sample in a diamond anvil cell (DAC). Aperture is limited by the DAC geometry. Low energy for data acquisition (25.6 keV)

```
PLAT975_ALERT_2_A Check Calcd Resid. Dens.  0.52Ang From N2      .       2.50 eA-3
```

Author Response: Multigrain sample in a DAC. Strong electronic contrast between Bi and N and limited completeness. Low energy for data acquisition (25.6 keV)

```
PLAT975_ALERT_2_A Check Calcd Resid. Dens.  0.66Ang From N1      .       2.40 eA-3
```

Author Response: Multigrain sample in a DAC. Strong electronic contrast between Bi and N and limited completeness. Low energy for data acquisition (25.6 keV)

Alert level B

```
PLAT911_ALERT_3_B Missing FCF Refl Between Thmin & STh/L=    0.600        187 Report
```

Author Response: Multigrain sample in a DAC. Strong electronic contrast between Bi and N and limited completeness.

Alert level C

```
GOODF01_ALERT_2_C  The least squares goodness of fit parameter lies
            outside the range 0.80 <> 2.00
            Goodness of fit given =      2.810
PLAT041_ALERT_1_C Calc. and Reported SumFormula    Strings  Differ     Please Check
PLAT127_ALERT_1_C Implicit Hall Symbol  Inconsistent with Explicit -P -2xab;- Check
PLAT905_ALERT_3_C Negative K value in the Analysis of Variance ...     -0.489 Report
PLAT913_ALERT_3_C Missing # of Very Strong Reflections in FCF ....          7 Note
PLAT918_ALERT_3_C Reflection(s) with I(obs) much Smaller I(calc) .          1 Check
PLAT921_ALERT_1_C R1  in the CIF and FCF Differ by ..............     0.0013 Check
PLAT923_ALERT_1_C S    Values in the CIF and FCF Differ by .......      0.013 Check
PLAT926_ALERT_1_C Reported and Calculated   R1 Differ by .........     0.0012 Check
PLAT939_ALERT_3_C Large Value of Not (SHELXL) Weight Optimized S .      16.15 Check
PLAT971_ALERT_2_C Check Calcd Resid. Dens.  0.52Ang From N2             2.50 eA-3
PLAT971_ALERT_2_C Check Calcd Resid. Dens.  0.66Ang From N1             2.40 eA-3
PLAT976_ALERT_2_C Check Calcd Resid. Dens.  0.42Ang From N1      .      -1.10 eA-3
PLAT976_ALERT_2_C Check Calcd Resid. Dens.  0.66Ang From N2      .      -1.09 eA-3
PLAT976_ALERT_2_C Check Calcd Resid. Dens.  0.49Ang From N2      .      -0.89 eA-3
PLAT976_ALERT_2_C Check Calcd Resid. Dens.  0.81Ang From N1      .      -0.83 eA-3
```

Alert level G

```
ABSMU01_ALERT_1_G  Calculation of _exptl_absorpt_correction_mu
                not performed for this radiation type.
PLAT004_ALERT_5_G Polymeric Structure Found with Maximum Dimension          3 Info
PLAT005_ALERT_5_G No Embedded Refinement Details Found  in the CIF     Please Do !
PLAT045_ALERT_1_G Calculated and Reported Z Differ by a Factor ...      0.500 Check
PLAT063_ALERT_4_G Crystal Size Possibly too Large for Beam Size ..       2.00 mm
PLAT092_ALERT_4_G Check: Wavelength Given is not Cu,Ga,Mo,Ag,In Ka    0.48390 Ang.
PLAT152_ALERT_1_G The Supplied and Calc. Volume s.u. Differ by ...          2 Units
PLAT199_ALERT_1_G Reported _cell_measurement_temperature ..... (K)        293 Check
PLAT200_ALERT_1_G Reported   _diffrn_ambient_temperature ..... (K)        293 Check
PLAT794_ALERT_5_G Tentative Bond Valency for Bi1       (III)     .       2.21 Info
PLAT794_ALERT_5_G Tentative Bond Valency for Bi2       (III)     .       2.04 Info
PLAT883_ALERT_1_G No Info/Value for _atom_sites_solution_primary .     Please Do !
PLAT910_ALERT_3_G Missing # of FCF Reflection(s) Below Theta(Min).          1 Note
PLAT912_ALERT_4_G Missing # of FCF Reflections Above STh/L=  0.600         30 Note
PLAT951_ALERT_5_G Calculated (ThMax) and CIF-Reported Kmax Differ           5 Units
PLAT957_ALERT_1_G Calculated (ThMax) and Actual (FCF) Kmax Differ           5 Units
PLAT960_ALERT_3_G Number of Intensities with I  <   - 2*sig(I) ...          1 Check
PLAT984_ALERT_1_G The Bi-f'=   -0.8138 Deviates from the B&C-Value    -0.7371 Check
PLAT985_ALERT_1_G The Bi-f"=    5.7893 Deviates from the B&C-Value     5.7397 Check
PLAT992_ALERT_5_G Repd & Actual _reflns_number_gt Values Differ by          4 Check
```

---

5 **ALERT level A** = Most likely a serious problem - resolve or explain
1 **ALERT level B** = A potentially serious problem, consider carefully
16 **ALERT level C** = Check. Ensure it is not caused by an omission or oversight
20 **ALERT level G** = General information/check it is not something unexpected

14 ALERT type 1 CIF construction/syntax error, inconsistent or missing data
9 ALERT type 2 Indicator that the structure model may be wrong or deficient
10 ALERT type 3 Indicator that the structure quality may be low
3 ALERT type 4 Improvement, methodology, query or suggestion
6 ALERT type 5 Informative message, check

It is advisable to attempt to resolve as many as possible of the alerts in all categories. Often the minor alerts point to easily fixed oversights, errors and omissions in your CIF or refinement strategy, so attention to these fine details can be worthwhile. In order to resolve some of the more serious problems it may be necessary to carry out additional measurements or structure refinements. However, the purpose of your study may justify the reported deviations and the more serious of these should normally be commented upon in the discussion or experimental section of a paper or in the "special_details" fields of the CIF. checkCIF was carefully designed to identify outliers and unusual parameters, but every test has its limitations and alerts that are not important in a particular case may appear. Conversely, the absence of alerts does not guarantee there are no aspects of the results needing attention. It is up to the individual to critically assess their own results and, if necessary, seek expert advice.

Publication of your CIF in IUCr journals

A basic structural check has been run on your CIF. These basic checks will be run on all CIFs submitted for publication in IUCr journals (*Acta Crystallographica*, *Journal of Applied Crystallography*, *Journal of Synchrotron Radiation*); however, if you intend to submit to *Acta Crystallographica Section C* or *E* or *IUCrData*, you should make sure that full publication checks are run on the final version of your CIF prior to submission.

Publication of your CIF in other journals

Please refer to the *Notes for Authors* of the relevant journal for any special instructions relating to CIF submission.

PLATON version of 10/05/2023; check.def file version of 10/05/2023

Datablock I - ellipsoid plot

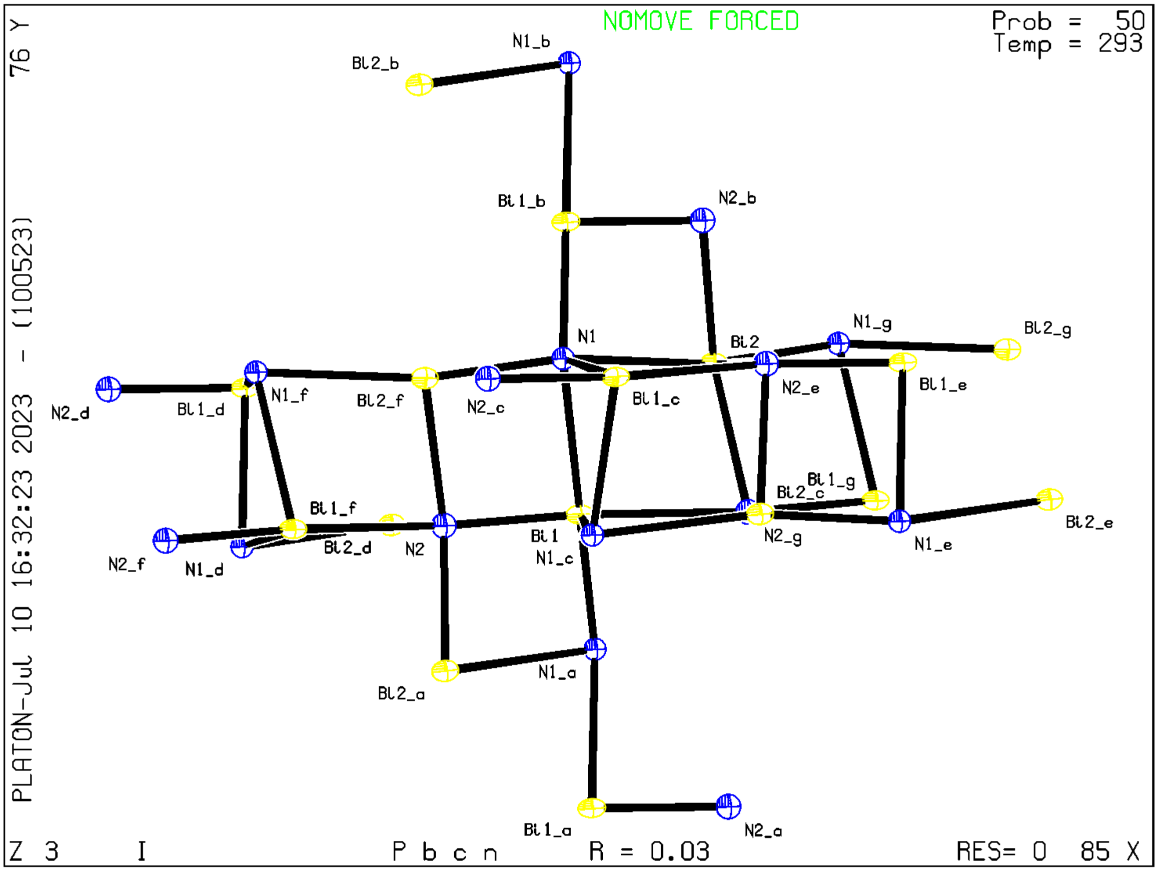

Supplement: Supplementary file 7 [file DataSheet3.PDF]
