# Supplementary material for: High-pressure reactions between the pnictogens: the rediscovery of BiN
Source: Front Chem. 2023 Oct 12;11:1257942. doi: 10.3389/fchem.2023.1257942 (PMC10602720; doi:10.3389/fchem.2023.1257942)

checkCIF/PLATON report

Structure factors have been supplied for datablock(s) I

THIS REPORT IS FOR GUIDANCE ONLY. IF USED AS PART OF A REVIEW PROCEDURE FOR PUBLICATION, IT SHOULD NOT REPLACE THE EXPERTISE OF AN EXPERIENCED CRYSTALLOGRAPHIC REFEREE.

No syntax errors found. CIF dictionary Interpreting this report

Datablock: I

Bond precision: Bi- N = 0.0158 A Wavelength=0.29040

Cell:	a=10.5329(6)	b=5.3629(6)	c=10.3989(4)
	alpha=90	beta=90	gamma=90
Temperature:	293 K		

	Calculated	Reported
Volume	587.40(8)	587.40(8)
Space group	P c a 21	P c a 21
Hall group	P 2c -2ac	P -2xac;-2y
Moiety formula	Bi4 N4	?
Sum formula	Bi4 N4	Bi1 N1
Mr	891.96	223.00
Dx,g cm-3	10.086	10.086
Z	4	16
Mu (mm-1)	11.801	11.950
F000	1440.0	1440.0
F000’	1426.84	
h,k,lmax	21,10,21	19,7,20
Nref	5149[2668]	1580
Tmin,Tmax		0.877,1.000
Tmin’		

Correction method= # Reported T Limits: Tmin=0.877 Tmax=1.000
AbsCorr = MULTI-SCAN

Data completeness= 0.59/0.31 Theta(max)= 17.170

R(reflections)= 0.0289(1443) wR2(reflections)= 0.0509(1580)

S = 1.870 Npar= 52

The following ALERTS were generated. Each ALERT has the format
test-name_ALERT_alert-type_alert-level.
Click on the hyperlinks for more details of the test.

Alert level A

PLAT029_ALERT_3_A _diffrn_measured_fraction_theta_full value Low . 0.620 Why?

Author Response: multigrain sample in a diamond anvil cell

PLAT975_ALERT_2_A Check Calcd Resid. Dens. 0.60Ang From N2 . 2.06 eA-3

Author Response: Strong electronic density contrast Bi/N, multigrain sample under high pressure conditions (diamond anvil cell)

PLAT975_ALERT_2_A Check Calcd Resid. Dens. 0.66Ang From N2 . 2.06 eA-3

Author Response: Strong electronic density contrast Bi/N, multigrain sample under high pressure conditions (diamond anvil cell)

Alert level B

PLAT911_ALERT_3_B Missing FCF Refl Between Thmin & STh/L= 0.600 214 Report

Author Response: High pressure experiment on multigrain sample in a diamond anvil cell.

PLAT934_ALERT_3_B Number of (Iobs-Icalc)/Sigma(W) > 10 Outliers .. 3 Check

Author Response: High pressure experiment on multigrain sample in a diamond anvil cell.

PLAT975_ALERT_2_B Check Calcd Resid. Dens. 0.44Ang From N1 . 1.85 eA-3

Author Response: Strong electronic density contrast Bi/N, multigrain sample under high pressure conditions (diamond anvil cell)

PLAT975_ALERT_2_B Check Calcd Resid. Dens. 0.85Ang From N3 . 1.75 eA-3

Author Response: Strong electronic density contrast Bi/N, multigrain sample under high pressure conditions (diamond anvil cell)

PLAT975_ALERT_2_B Check Calcd Resid. Dens. 0.70Ang From N1 . 1.69 eA-3

Author Response: Strong electronic density contrast Bi/N, multigrain sample under high pressure conditions (diamond anvil cell)

```
PLAT976_ALERT_2_B Check Calcd Resid. Dens.  0.76Ang From N4      .      -1.62 eA-3
```

Author Response: Strong electronic density contrast Bi/N, multigrain sample under high pressure conditions (diamond anvil cell)

```
PLAT992_ALERT_5_B Repd & Actual _reflns_number_gt Values Differ by         53 Check
```

Author Response: Probably an artifact by JANA2006. Dataset was prepared by this softwar

---

Alert level C

```
PLAT034_ALERT_1_C No Flack Parameter Given. Z > Si, NonCentro ....     Please Do !
PLAT041_ALERT_1_C Calc. and Reported SumFormula    Strings  Differ     Please Check
PLAT051_ALERT_1_C Mu(calc) and Mu(CIF) Ratio Differs from 1.0 by .       1.25 %
PLAT053_ALERT_1_C Minimum Crystal Dimension Missing (or Error) ...     Please Check
PLAT054_ALERT_1_C Medium  Crystal Dimension Missing (or Error) ...     Please Check
PLAT055_ALERT_1_C Maximum Crystal Dimension Missing (or Error) ...     Please Check
PLAT127_ALERT_1_C Implicit Hall Symbol  Inconsistent with Explicit P -2xac;-2 Check
PLAT324_ALERT_2_C Check for Possibly Missing H on Coordinating....         N1 Check
PLAT324_ALERT_2_C Check for Possibly Missing H on Coordinating....         N2 Check
PLAT324_ALERT_2_C Check for Possibly Missing H on Coordinating....         N3 Check
PLAT324_ALERT_2_C Check for Possibly Missing H on Coordinating....         N4 Check
PLAT913_ALERT_3_C Missing # of Very Strong Reflections in FCF ....         12 Note
PLAT921_ALERT_1_C R1  in the CIF and FCF Differ by ..............     0.0013 Check
PLAT926_ALERT_1_C Reported and Calculated   R1 Differ by .........     0.0011 Check
PLAT971_ALERT_2_C Check Calcd Resid. Dens.  0.69Ang From Bi1           2.32 eA-3
PLAT971_ALERT_2_C Check Calcd Resid. Dens.  1.07Ang From N3            2.17 eA-3
PLAT971_ALERT_2_C Check Calcd Resid. Dens.  0.60Ang From Bi2           2.07 eA-3
PLAT971_ALERT_2_C Check Calcd Resid. Dens.  0.60Ang From N2            2.06 eA-3
PLAT971_ALERT_2_C Check Calcd Resid. Dens.  0.66Ang From N2            2.06 eA-3
PLAT971_ALERT_2_C Check Calcd Resid. Dens.  1.02Ang From N1            2.04 eA-3
PLAT971_ALERT_2_C Check Calcd Resid. Dens.  1.34Ang From Bi3           2.04 eA-3
PLAT971_ALERT_2_C Check Calcd Resid. Dens.  1.37Ang From Bi3           1.94 eA-3
PLAT971_ALERT_2_C Check Calcd Resid. Dens.  1.24Ang From Bi3           1.94 eA-3
PLAT971_ALERT_2_C Check Calcd Resid. Dens.  1.40Ang From Bi3           1.93 eA-3
PLAT971_ALERT_2_C Check Calcd Resid. Dens.  0.64Ang From Bi3           1.92 eA-3
PLAT971_ALERT_2_C Check Calcd Resid. Dens.  0.60Ang From Bi4           1.90 eA-3
PLAT971_ALERT_2_C Check Calcd Resid. Dens.  1.07Ang From Bi3           1.86 eA-3
PLAT971_ALERT_2_C Check Calcd Resid. Dens.  0.44Ang From N1            1.85 eA-3
PLAT971_ALERT_2_C Check Calcd Resid. Dens.  1.34Ang From Bi1           1.82 eA-3
PLAT971_ALERT_2_C Check Calcd Resid. Dens.  1.37Ang From N1            1.79 eA-3
PLAT971_ALERT_2_C Check Calcd Resid. Dens.  0.47Ang From Bi2           1.79 eA-3
PLAT971_ALERT_2_C Check Calcd Resid. Dens.  0.85Ang From N3            1.75 eA-3
PLAT971_ALERT_2_C Check Calcd Resid. Dens.  1.08Ang From N4            1.75 eA-3
PLAT971_ALERT_2_C Check Calcd Resid. Dens.  1.09Ang From N4            1.75 eA-3
PLAT971_ALERT_2_C Check Calcd Resid. Dens.  1.34Ang From Bi4           1.74 eA-3
PLAT971_ALERT_2_C Check Calcd Resid. Dens.  1.31Ang From Bi4           1.72 eA-3
PLAT971_ALERT_2_C Check Calcd Resid. Dens.  0.74Ang From Bi1           1.71 eA-3
PLAT971_ALERT_2_C Check Calcd Resid. Dens.  0.99Ang From Bi2           1.71 eA-3
```

```
PLAT971_ALERT_2_C Check Calcd Resid. Dens.  0.70Ang From N1             1.69 eA-3
PLAT972_ALERT_2_C Check Calcd Resid. Dens.  0.86Ang From Bi1           -2.28 eA-3
PLAT972_ALERT_2_C Check Calcd Resid. Dens.  0.92Ang From Bi2           -2.20 eA-3
PLAT972_ALERT_2_C Check Calcd Resid. Dens.  1.44Ang From N3            -2.12 eA-3
PLAT972_ALERT_2_C Check Calcd Resid. Dens.  0.98Ang From Bi3           -2.10 eA-3
PLAT972_ALERT_2_C Check Calcd Resid. Dens.  1.79Ang From N2            -2.05 eA-3
PLAT972_ALERT_2_C Check Calcd Resid. Dens.  0.93Ang From Bi4           -2.05 eA-3
PLAT972_ALERT_2_C Check Calcd Resid. Dens.  1.15Ang From Bi1           -2.04 eA-3
PLAT972_ALERT_2_C Check Calcd Resid. Dens.  1.00Ang From Bi3           -2.03 eA-3
PLAT972_ALERT_2_C Check Calcd Resid. Dens.  1.19Ang From Bi4           -2.02 eA-3
PLAT972_ALERT_2_C Check Calcd Resid. Dens.  0.89Ang From Bi1           -2.01 eA-3
PLAT972_ALERT_2_C Check Calcd Resid. Dens.  0.95Ang From Bi3           -1.98 eA-3
PLAT972_ALERT_2_C Check Calcd Resid. Dens.  1.10Ang From Bi4           -1.93 eA-3
PLAT972_ALERT_2_C Check Calcd Resid. Dens.  1.48Ang From Bi3           -1.92 eA-3
PLAT972_ALERT_2_C Check Calcd Resid. Dens.  1.64Ang From Bi3           -1.89 eA-3
PLAT972_ALERT_2_C Check Calcd Resid. Dens.  1.13Ang From N3            -1.87 eA-3
PLAT972_ALERT_2_C Check Calcd Resid. Dens.  0.70Ang From Bi3           -1.81 eA-3
PLAT972_ALERT_2_C Check Calcd Resid. Dens.  1.12Ang From Bi2           -1.79 eA-3
PLAT972_ALERT_2_C Check Calcd Resid. Dens.  0.94Ang From N1            -1.78 eA-3
PLAT972_ALERT_2_C Check Calcd Resid. Dens.  1.24Ang From Bi3           -1.74 eA-3
PLAT972_ALERT_2_C Check Calcd Resid. Dens.  1.62Ang From Bi2           -1.71 eA-3
PLAT972_ALERT_2_C Check Calcd Resid. Dens.  1.08Ang From Bi1           -1.69 eA-3
PLAT972_ALERT_2_C Check Calcd Resid. Dens.  0.94Ang From Bi1           -1.68 eA-3
PLAT972_ALERT_2_C Check Calcd Resid. Dens.  1.61Ang From N1            -1.67 eA-3
PLAT972_ALERT_2_C Check Calcd Resid. Dens.  0.76Ang From N4            -1.62 eA-3
PLAT972_ALERT_2_C Check Calcd Resid. Dens.  1.74Ang From Bi4           -1.61 eA-3
```

Alert level G

```
ABSMU01_ALERT_1_G  Calculation of _exptl_absorpt_correction_mu
                not performed for this radiation type.
PLAT004_ALERT_5_G Polymeric Structure Found with Maximum Dimension          3 Info
PLAT005_ALERT_5_G No Embedded Refinement Details Found  in the CIF     Please Do !
PLAT045_ALERT_1_G Calculated and Reported Z Differ by a Factor ...      0.250 Check
PLAT092_ALERT_4_G Check: Wavelength Given is not Cu,Ga,Mo,Ag,In Ka    0.29040 Ang.
PLAT199_ALERT_1_G Reported _cell_measurement_temperature ..... (K)        293 Check
PLAT200_ALERT_1_G Reported   _diffrn_ambient_temperature ..... (K)        293 Check
PLAT883_ALERT_1_G No Info/Value for _atom_sites_solution_primary .     Please Do !
PLAT910_ALERT_3_G Missing # of FCF Reflection(s) Below Theta(Min).          4 Note
PLAT912_ALERT_4_G Missing # of FCF Reflections Above STh/L=  0.600       1054 Note
PLAT915_ALERT_3_G No Flack x Check Done: Low Friedel Pair Coverage         23 %
PLAT950_ALERT_5_G Calculated (ThMax) and CIF-Reported Hmax Differ           2 Units
PLAT951_ALERT_5_G Calculated (ThMax) and CIF-Reported Kmax Differ           3 Units
PLAT956_ALERT_1_G Calculated (ThMax) and Actual (FCF) Hmax Differ           2 Units
PLAT957_ALERT_1_G Calculated (ThMax) and Actual (FCF) Kmax Differ           3 Units
PLAT961_ALERT_5_G Dataset Contains no Negative Intensities .......     Please Check
PLAT984_ALERT_1_G The Bi-f'=   -0.8743 Deviates from the B&C-Value    -0.8048 Check
PLAT985_ALERT_1_G The Bi-f"=    2.4721 Deviates from the B&C-Value     2.4496 Check
```

---

 3 **ALERT level A** = Most likely a serious problem - resolve or explain
 7 **ALERT level B** = A potentially serious problem, consider carefully
64 **ALERT level C** = Check. Ensure it is not caused by an omission or oversight
18 **ALERT level G** = General information/check it is not something unexpected

18 ALERT type 1 CIF construction/syntax error, inconsistent or missing data

```
60 ALERT type 2 Indicator that the structure model may be wrong or deficient
 6 ALERT type 3 Indicator that the structure quality may be low
 2 ALERT type 4 Improvement, methodology, query or suggestion
 6 ALERT type 5 Informative message, check
```

It is advisable to attempt to resolve as many as possible of the alerts in all categories. Often the minor alerts point to easily fixed oversights, errors and omissions in your CIF or refinement strategy, so attention to these fine details can be worthwhile. In order to resolve some of the more serious problems it may be necessary to carry out additional measurements or structure refinements. However, the purpose of your study may justify the reported deviations and the more serious of these should normally be commented upon in the discussion or experimental section of a paper or in the "special_details" fields of the CIF. checkCIF was carefully designed to identify outliers and unusual parameters, but every test has its limitations and alerts that are not important in a particular case may appear. Conversely, the absence of alerts does not guarantee there are no aspects of the results needing attention. It is up to the individual to critically assess their own results and, if necessary, seek expert advice.

Publication of your CIF in IUCr journals

A basic structural check has been run on your CIF. These basic checks will be run on all CIFs submitted for publication in IUCr journals (*Acta Crystallographica*, *Journal of Applied Crystallography*, *Journal of Synchrotron Radiation*); however, if you intend to submit to *Acta Crystallographica Section C* or *E* or *IUCrData*, you should make sure that full publication checks are run on the final version of your CIF prior to submission.

Publication of your CIF in other journals

Please refer to the *Notes for Authors* of the relevant journal for any special instructions relating to CIF submission.

PLATON version of 10/05/2023; check.def file version of 10/05/2023

Datablock I - ellipsoid plot

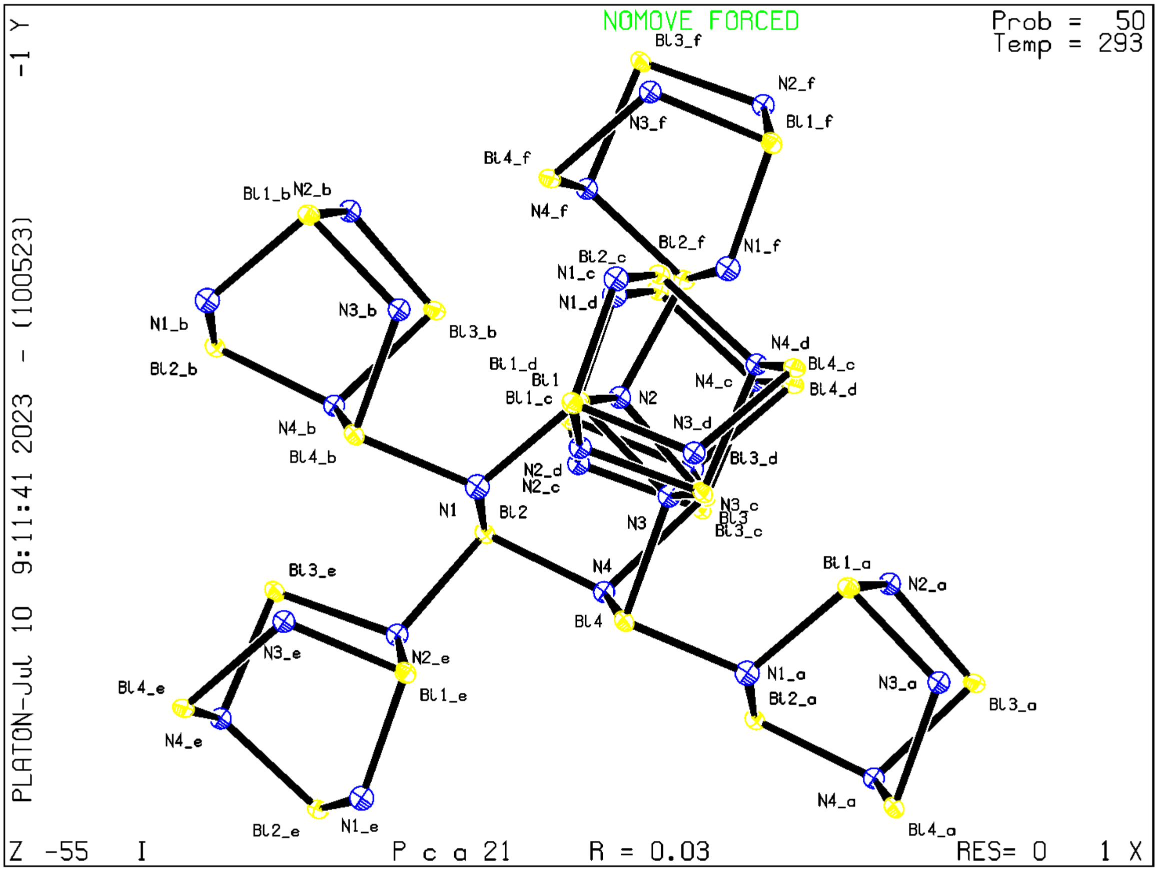

Supplement: Supplementary file 8 [file DataSheet1.PDF]
