# Supplementary material for: High-pressure reactions between the pnictogens: the rediscovery of BiN
Source: Front Chem. 2023 Oct 12;11:1257942. doi: 10.3389/fchem.2023.1257942 (PMC10602720; doi:10.3389/fchem.2023.1257942)

checkCIF/PLATON report

Structure factors have been supplied for datablock(s) I

THIS REPORT IS FOR GUIDANCE ONLY. IF USED AS PART OF A REVIEW PROCEDURE FOR PUBLICATION, IT SHOULD NOT REPLACE THE EXPERTISE OF AN EXPERIENCED CRYSTALLOGRAPHIC REFEREE.

No syntax errors found. CIF dictionary Interpreting this report

Datablock: I

Bond precision: Bi- N = 0.0307 A Wavelength=0.29040

Cell:	a=4.8911(19)	b=9.471(3)	c=9.202(15)
	alpha=90	beta=90	gamma=90
Temperature:	293 K		

	Calculated	Reported
Volume	426.3(7)	426.3(7)
Space group	P b c n	P b c n
Hall group	-P 2n 2ab	-P -2xab;-2
Moiety formula	Bi2 N2	?
Sum formula	Bi2 N2	Bi1 N1
Mr	445.98	223.00
Dx,g cm-3	13.900	13.899
Z	8	16
Mu (mm-1)	16.260	16.467
F000	1440.0	1440.0
F000'	1426.84	
h,k,lmax	10,19,19	10,18,5
Nref	2009	345
Tmin,Tmax		0.003,0.008
Tmin'		

Correction method= # Reported T Limits: Tmin=0.003 Tmax=0.008
AbsCorr = SPHERE

Data completeness= 0.172 Theta(max)= 17.580

R(reflections)= 0.0372(246) wR2(reflections)= 0.0754(345)

S = 1.730 Npar= 27

The following ALERTS were generated. Each ALERT has the format
test-name_ALERT_alert-type_alert-level.
Click on the hyperlinks for more details of the test.

Alert level A

PLAT027_ALERT_3_A _diffrn_reflns_theta_full value (too) Low ...... 6.57 Degree

Author Response: Multigrain sample in a diamond anvil cell (DAC). Aperture is limited by the DAC geometry.

PLAT029_ALERT_3_A _diffrn_measured_fraction_theta_full value Low . 0.400 Why?

Author Response: Multigrain sample in a diamond anvil cell (DAC). Aperture is limited by the DAC geometry.

Alert level B

PLAT911_ALERT_3_B Missing FCF Refl Between Thmin & STh/L= 0.600 145 Report

Author Response: Multigrain sample in a diamond anvil cell (DAC). Aperture is limited by the DAC geometry.

PLAT972_ALERT_2_B Check Calcd Resid. Dens. 0.84Ang From Bi1 -2.69 eA-3

Author Response: Multigrain sample in a DAC. Strong electronic contrast between Bi and N and limited completeness.

PLAT975_ALERT_2_B Check Calcd Resid. Dens. 0.58Ang From N1 . 1.65 eA-3

Author Response: Multigrain sample in a DAC. Strong electronic contrast between Bi and N and limited completeness.

PLAT976_ALERT_2_B Check Calcd Resid. Dens. 0.85Ang From N2 . -1.71 eA-3

Author Response: Multigrain sample in a DAC. Strong electronic contrast between Bi and N and limited completeness.

PLAT976_ALERT_2_B Check Calcd Resid. Dens. 0.47Ang From N2 . -1.56 eA-3

Author Response: Multigrain sample in a DAC. Strong electronic contrast between Bi and N and limited completeness.

Alert level C

```
PLAT041_ALERT_1_C Calc. and Reported SumFormula    Strings  Differ     Please Check
PLAT051_ALERT_1_C Mu(calc) and Mu(CIF) Ratio Differs from 1.0 by .       1.26 %
PLAT127_ALERT_1_C Implicit Hall Symbol  Inconsistent with Explicit -P -2xab;- Check
PLAT148_ALERT_3_C s.u. on the      c    - Axis is (Too) Large ....      0.015 Ang.
PLAT906_ALERT_3_C Large K Value in the Analysis of Variance ......      7.669 Check
PLAT921_ALERT_1_C R1  in the CIF and FCF Differ by ..............     0.0031 Check
PLAT926_ALERT_1_C Reported and Calculated   R1 Differ by .........     0.0031 Check
PLAT971_ALERT_2_C Check Calcd Resid. Dens.  1.43Ang From N1              1.78 eA-3
PLAT971_ALERT_2_C Check Calcd Resid. Dens.  1.32Ang From Bi2             1.78 eA-3
PLAT971_ALERT_2_C Check Calcd Resid. Dens.  1.30Ang From N1              1.73 eA-3
PLAT971_ALERT_2_C Check Calcd Resid. Dens.  1.48Ang From Bi1             1.68 eA-3
PLAT971_ALERT_2_C Check Calcd Resid. Dens.  0.58Ang From N1              1.65 eA-3
PLAT971_ALERT_2_C Check Calcd Resid. Dens.  1.13Ang From N1              1.60 eA-3
PLAT971_ALERT_2_C Check Calcd Resid. Dens.  0.54Ang From Bi2             1.58 eA-3
PLAT971_ALERT_2_C Check Calcd Resid. Dens.  1.17Ang From N2              1.54 eA-3
PLAT972_ALERT_2_C Check Calcd Resid. Dens.  0.96Ang From Bi1            -2.31 eA-3
```

Author Response: Multigrain sample in a DAC. Strong electronic contrast between Bi and N and limited completeness.

```
PLAT972_ALERT_2_C Check Calcd Resid. Dens.  1.06Ang From Bi2            -2.07 eA-3
```

Author Response: Multigrain sample in a DAC. Strong electronic contrast between Bi and N and limited completeness.

```
PLAT972_ALERT_2_C Check Calcd Resid. Dens.  0.97Ang From Bi1            -1.83 eA-3
```

Author Response: Multigrain sample in a DAC. Strong electronic contrast between Bi and N and limited completeness.

```
PLAT972_ALERT_2_C Check Calcd Resid. Dens.  0.85Ang From Bi2            -1.79 eA-3
```

Author Response: Multigrain sample in a DAC. Strong electronic contrast between Bi and N and limited completeness.

```
PLAT972_ALERT_2_C Check Calcd Resid. Dens.  0.85Ang From N2             -1.71 eA-3
```

Author Response: Multigrain sample in a DAC. Strong electronic contrast between Bi and N and limited completeness.

```
PLAT972_ALERT_2_C Check Calcd Resid. Dens.  0.90Ang From Bi2            -1.67 eA-3
```

Author Response: Multigrain sample in a DAC. Strong electronic contrast between Bi and N and limited completeness.

PLAT972_ALERT_2_C Check Calcd Resid. Dens. 1.18Ang From N1 -1.60 eA-3

Author Response: Multigrain sample in a DAC. Strong electronic contrast between Bi and N and limited completeness.

PLAT972_ALERT_2_C Check Calcd Resid. Dens. 1.48Ang From N2 -1.57 eA-3

Author Response: Multigrain sample in a DAC. Strong electronic contrast between Bi and N and limited completeness.

PLAT972_ALERT_2_C Check Calcd Resid. Dens. 0.47Ang From N2 -1.56 eA-3

Author Response: Multigrain sample in a DAC. Strong electronic contrast between Bi and N and limited completeness.

PLAT972_ALERT_2_C Check Calcd Resid. Dens. 1.28Ang From N1 -1.53 eA-3

Author Response: Multigrain sample in a DAC. Strong electronic contrast between Bi and N and limited completeness.

PLAT972_ALERT_2_C Check Calcd Resid. Dens. 1.30Ang From N1 -1.53 eA-3

Author Response: Multigrain sample in a DAC. Strong electronic contrast between Bi and N and limited completeness.

PLAT975_ALERT_2_C Check Calcd Resid. Dens. 0.64Ang From N2 . 1.42 eA-3

Author Response: Multigrain sample in a DAC. Strong electronic contrast between Bi and N and limited completeness.

PLAT976_ALERT_2_C Check Calcd Resid. Dens. 0.73Ang From N1 . -1.26 eA-3

Author Response: Multigrain sample in a DAC. Strong electronic contrast between Bi and N and limited completeness.

PLAT992_ALERT_5_C Repd & Actual _reflns_number_gt Values Differ by 26 Check

---

Alert level G

```
ABSMU01_ALERT_1_G  Calculation of _exptl_absorpt_correction_mu
                   not performed for this radiation type.
PLAT004_ALERT_5_G Polymeric Structure Found with Maximum Dimension          3 Info
PLAT005_ALERT_5_G No Embedded Refinement Details Found  in the CIF     Please Do !
PLAT045_ALERT_1_G Calculated and Reported Z Differ by a Factor ...      0.500 Check
PLAT063_ALERT_4_G Crystal Size Possibly too Large for Beam Size ..       2.00 mm
PLAT092_ALERT_4_G Check: Wavelength Given is not Cu,Ga,Mo,Ag,In Ka    0.29040 Ang.
```

```
PLAT199_ALERT_1_G Reported _cell_measurement_temperature ..... (K)        293 Check
PLAT200_ALERT_1_G Reported   _diffrn_ambient_temperature ..... (K)        293 Check
PLAT794_ALERT_5_G Tentative Bond Valency for Bi1       (III)     .       2.58 Info
PLAT794_ALERT_5_G Tentative Bond Valency for Bi2       (III)     .       2.16 Info
PLAT883_ALERT_1_G No Info/Value for _atom_sites_solution_primary .     Please Do !
PLAT910_ALERT_3_G Missing # of FCF Reflection(s) Below Theta(Min).          2 Note
PLAT912_ALERT_4_G Missing # of FCF Reflections Above STh/L=  0.600        342 Note
PLAT913_ALERT_3_G Missing # of Very Strong Reflections in FCF ....          2 Note
PLAT952_ALERT_5_G Calculated (ThMax) and CIF-Reported Lmax Differ.         14 Units
PLAT958_ALERT_1_G Calculated (ThMax) and Actual (FCF) Lmax Differ.         14 Units
PLAT961_ALERT_5_G Dataset Contains no Negative Intensities .......     Please Check
PLAT984_ALERT_1_G The Bi-f'=   -0.8743 Deviates from the B&C-Value    -0.8048 Check
PLAT985_ALERT_1_G The Bi-f"=    2.4721 Deviates from the B&C-Value     2.4496 Check
```

```
   2 ALERT level A = Most likely a serious problem - resolve or explain
   5 ALERT level B = A potentially serious problem, consider carefully
  29 ALERT level C = Check. Ensure it is not caused by an omission or oversight
  19 ALERT level G = General information/check it is not something unexpected

  13 ALERT type 1 CIF construction/syntax error, inconsistent or missing data
  25 ALERT type 2 Indicator that the structure model may be wrong or deficient
   7 ALERT type 3 Indicator that the structure quality may be low
   3 ALERT type 4 Improvement, methodology, query or suggestion
   7 ALERT type 5 Informative message, check
```

It is advisable to attempt to resolve as many as possible of the alerts in all categories. Often the minor alerts point to easily fixed oversights, errors and omissions in your CIF or refinement strategy, so attention to these fine details can be worthwhile. In order to resolve some of the more serious problems it may be necessary to carry out additional measurements or structure refinements. However, the purpose of your study may justify the reported deviations and the more serious of these should normally be commented upon in the discussion or experimental section of a paper or in the "special_details" fields of the CIF. checkCIF was carefully designed to identify outliers and unusual parameters, but every test has its limitations and alerts that are not important in a particular case may appear. Conversely, the absence of alerts does not guarantee there are no aspects of the results needing attention. It is up to the individual to critically assess their own results and, if necessary, seek expert advice.

Publication of your CIF in IUCr journals

A basic structural check has been run on your CIF. These basic checks will be run on all CIFs submitted for publication in IUCr journals (*Acta Crystallographica*, *Journal of Applied Crystallography*, *Journal of Synchrotron Radiation*); however, if you intend to submit to *Acta Crystallographica Section C* or *E* or *IUCrData*, you should make sure that full publication checks are run on the final version of your CIF prior to submission.

Publication of your CIF in other journals

Please refer to the *Notes for Authors* of the relevant journal for any special instructions relating to CIF submission.

PLATON version of 10/05/2023; check.def file version of 10/05/2023

Datablock I - ellipsoid plot

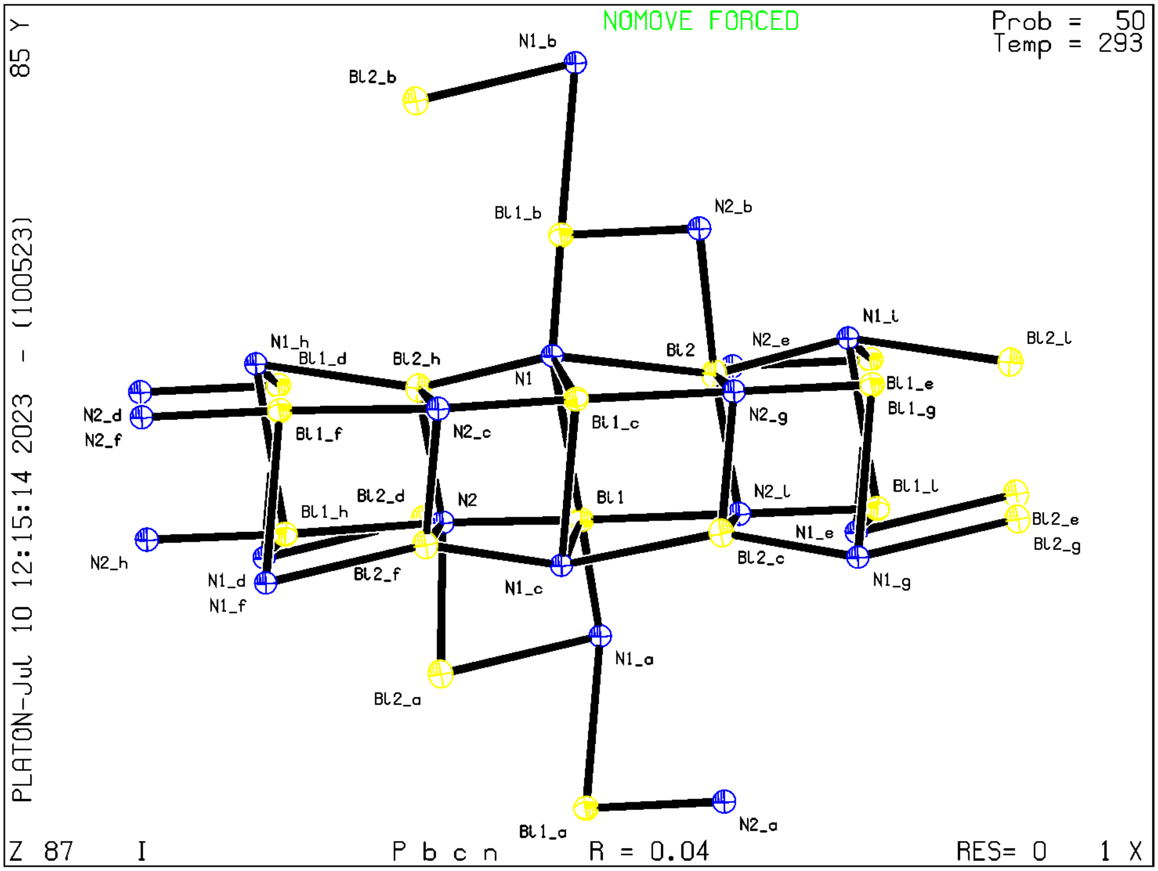

Supplement: Supplementary file 9 [file DataSheet5.PDF]
