# Supplementary material for: High-pressure reactions between the pnictogens: the rediscovery of BiN
Source: Front Chem. 2023 Oct 12;11:1257942. doi: 10.3389/fchem.2023.1257942 (PMC10602720; doi:10.3389/fchem.2023.1257942)

checkCIF/PLATON report

Structure factors have been supplied for datablock(s) I

THIS REPORT IS FOR GUIDANCE ONLY. IF USED AS PART OF A REVIEW PROCEDURE FOR PUBLICATION, IT SHOULD NOT REPLACE THE EXPERTISE OF AN EXPERIENCED CRYSTALLOGRAPHIC REFEREE.

No syntax errors found. CIF dictionary Interpreting this report

Datablock: I

Bond precision: Bi- N = 0.0109 A Wavelength=0.29090

Cell:	a=4.8941(5)	b=9.402(1)	c=9.176(4)
	alpha=90	beta=90	gamma=90
Temperature:	293 K		

	Calculated	Reported
Volume	422.23(19)	422.22(19)
Space group	P b c n	P b c n
Hall group	-P 2n 2ab	-P -2xab;-2
Moiety formula	Bi2 N2	?
Sum formula	Bi2 N2	Bi1 N1
Mr	445.98	223.00
Dx,g cm-3	14.032	14.032
Z	8	16
Mu (mm-1)	16.490	16.697
F000	1440.0	1440.0
F000’	1426.87	
h,k,lmax	9,19,18	8,16,10
Nref	1886	687
Tmin,Tmax		0.812,1.000
Tmin’		

Correction method= # Reported T Limits: Tmin=0.812 Tmax=1.000
AbsCorr = MULTI-SCAN

Data completeness= 0.364 Theta(max)= 17.280

R(reflections)= 0.0257(518) wR2(reflections)= 0.0579(687)

S = 0.960 Npar= 37

The following ALERTS were generated. Each ALERT has the format
test-name_ALERT_alert-type_alert-level.
Click on the hyperlinks for more details of the test.

Alert level A

```
PLAT029_ALERT_3_A _diffrn_measured_fraction_theta_full value Low .      0.540 Why?
```

Author Response: Multigrain sample in a diamond anvil cell (DAC). Aperture is limited by the DAC geometry.

Alert level B

```
PLAT911_ALERT_3_B Missing FCF Refl Between Thmin & STh/L=    0.600        176 Report
```

Author Response: Multigrain sample in a diamond anvil cell (DAC). Aperture is limited by the DAC geometry.

```
PLAT975_ALERT_2_B Check Calcd Resid. Dens.  0.85Ang From N2      .       1.77 eA-3
```

Author Response: Multigrain sample in a DAC. Strong electronic contrast between Bi and N and limited completeness.

```
PLAT975_ALERT_2_B Check Calcd Resid. Dens.  0.70Ang From N2      .       1.61 eA-3
```

Author Response: Multigrain sample in a DAC. Strong electronic contrast between Bi and N and limited completeness.

```
PLAT976_ALERT_2_B Check Calcd Resid. Dens.  1.03Ang From N1      .      -1.63 eA-3
```

Author Response: Multigrain sample in a DAC. Strong electronic contrast between Bi and N and limited completeness.

Alert level C

```
PLAT041_ALERT_1_C Calc. and Reported SumFormula    Strings  Differ     Please Check
PLAT051_ALERT_1_C Mu(calc) and Mu(CIF) Ratio Differs from 1.0 by .       1.24 %
PLAT053_ALERT_1_C Minimum Crystal Dimension Missing (or Error) ...     Please Check
PLAT054_ALERT_1_C Medium  Crystal Dimension Missing (or Error) ...     Please Check
PLAT055_ALERT_1_C Maximum Crystal Dimension Missing (or Error) ...     Please Check
PLAT127_ALERT_1_C Implicit Hall Symbol  Inconsistent with Explicit -P -2xab;- Check
PLAT905_ALERT_3_C Negative K value in the Analysis of Variance ...     -0.205 Report
PLAT913_ALERT_3_C Missing # of Very Strong Reflections in FCF ....          7 Note
PLAT921_ALERT_1_C R1  in the CIF and FCF Differ by ..............     0.0020 Check
PLAT926_ALERT_1_C Reported and Calculated   R1 Differ by .........     0.0020 Check
PLAT971_ALERT_2_C Check Calcd Resid. Dens.  0.72Ang From Bi2             2.38 eA-3
```

```
PLAT971_ALERT_2_C Check Calcd Resid. Dens.  0.79Ang From Bi1            2.31 eA-3
PLAT971_ALERT_2_C Check Calcd Resid. Dens.  0.64Ang From Bi2            2.25 eA-3
PLAT971_ALERT_2_C Check Calcd Resid. Dens.  0.79Ang From Bi1            2.04 eA-3
PLAT971_ALERT_2_C Check Calcd Resid. Dens.  1.85Ang From N1             1.99 eA-3
PLAT971_ALERT_2_C Check Calcd Resid. Dens.  1.23Ang From N1             1.94 eA-3
PLAT971_ALERT_2_C Check Calcd Resid. Dens.  0.92Ang From N2             1.90 eA-3
PLAT971_ALERT_2_C Check Calcd Resid. Dens.  0.82Ang From Bi2            1.90 eA-3
PLAT971_ALERT_2_C Check Calcd Resid. Dens.  1.00Ang From N1             1.80 eA-3
PLAT971_ALERT_2_C Check Calcd Resid. Dens.  1.37Ang From N2             1.80 eA-3
PLAT971_ALERT_2_C Check Calcd Resid. Dens.  0.85Ang From N2             1.77 eA-3
PLAT971_ALERT_2_C Check Calcd Resid. Dens.  0.68Ang From Bi2            1.76 eA-3
PLAT971_ALERT_2_C Check Calcd Resid. Dens.  1.06Ang From Bi1            1.75 eA-3
PLAT971_ALERT_2_C Check Calcd Resid. Dens.  0.95Ang From N1             1.74 eA-3
PLAT971_ALERT_2_C Check Calcd Resid. Dens.  0.95Ang From N1             1.74 eA-3
PLAT971_ALERT_2_C Check Calcd Resid. Dens.  0.97Ang From N1             1.74 eA-3
PLAT971_ALERT_2_C Check Calcd Resid. Dens.  1.72Ang From N2             1.73 eA-3
PLAT971_ALERT_2_C Check Calcd Resid. Dens.  1.76Ang From N2             1.68 eA-3
PLAT971_ALERT_2_C Check Calcd Resid. Dens.  0.61Ang From Bi1            1.68 eA-3
PLAT971_ALERT_2_C Check Calcd Resid. Dens.  0.61Ang From Bi1            1.68 eA-3
PLAT971_ALERT_2_C Check Calcd Resid. Dens.  0.63Ang From N1             1.68 eA-3
PLAT971_ALERT_2_C Check Calcd Resid. Dens.  1.22Ang From Bi2            1.66 eA-3
PLAT971_ALERT_2_C Check Calcd Resid. Dens.  1.46Ang From N1             1.66 eA-3
PLAT971_ALERT_2_C Check Calcd Resid. Dens.  0.70Ang From N2             1.61 eA-3
PLAT971_ALERT_2_C Check Calcd Resid. Dens.  1.35Ang From N1             1.61 eA-3
PLAT972_ALERT_2_C Check Calcd Resid. Dens.  0.61Ang From Bi2           -2.32 eA-3
PLAT972_ALERT_2_C Check Calcd Resid. Dens.  0.62Ang From Bi2           -2.32 eA-3
PLAT972_ALERT_2_C Check Calcd Resid. Dens.  1.29Ang From Bi2           -2.27 eA-3
PLAT972_ALERT_2_C Check Calcd Resid. Dens.  1.31Ang From N2            -2.09 eA-3
PLAT972_ALERT_2_C Check Calcd Resid. Dens.  1.07Ang From Bi1           -2.05 eA-3
PLAT972_ALERT_2_C Check Calcd Resid. Dens.  1.34Ang From N1            -2.03 eA-3
PLAT972_ALERT_2_C Check Calcd Resid. Dens.  1.18Ang From Bi1           -1.97 eA-3
PLAT972_ALERT_2_C Check Calcd Resid. Dens.  1.14Ang From Bi1           -1.97 eA-3
PLAT972_ALERT_2_C Check Calcd Resid. Dens.  1.05Ang From Bi1           -1.96 eA-3
PLAT972_ALERT_2_C Check Calcd Resid. Dens.  1.18Ang From Bi2           -1.90 eA-3
PLAT972_ALERT_2_C Check Calcd Resid. Dens.  1.18Ang From Bi2           -1.90 eA-3
PLAT972_ALERT_2_C Check Calcd Resid. Dens.  1.22Ang From N2            -1.88 eA-3
PLAT972_ALERT_2_C Check Calcd Resid. Dens.  1.18Ang From Bi1           -1.81 eA-3
PLAT972_ALERT_2_C Check Calcd Resid. Dens.  1.10Ang From Bi2           -1.80 eA-3
PLAT972_ALERT_2_C Check Calcd Resid. Dens.  0.96Ang From Bi2           -1.71 eA-3
PLAT972_ALERT_2_C Check Calcd Resid. Dens.  1.29Ang From Bi1           -1.70 eA-3
PLAT972_ALERT_2_C Check Calcd Resid. Dens.  1.27Ang From Bi2           -1.70 eA-3
PLAT972_ALERT_2_C Check Calcd Resid. Dens.  1.29Ang From Bi2           -1.68 eA-3
PLAT972_ALERT_2_C Check Calcd Resid. Dens.  1.42Ang From Bi1           -1.66 eA-3
PLAT972_ALERT_2_C Check Calcd Resid. Dens.  0.22Ang From Bi2           -1.66 eA-3
PLAT972_ALERT_2_C Check Calcd Resid. Dens.  1.24Ang From N1            -1.65 eA-3
PLAT972_ALERT_2_C Check Calcd Resid. Dens.  1.67Ang From N1            -1.64 eA-3
PLAT972_ALERT_2_C Check Calcd Resid. Dens.  1.16Ang From N1            -1.63 eA-3
PLAT972_ALERT_2_C Check Calcd Resid. Dens.  1.03Ang From N1            -1.63 eA-3
PLAT972_ALERT_2_C Check Calcd Resid. Dens.  1.62Ang From Bi1           -1.61 eA-3
PLAT992_ALERT_5_C Repd & Actual _reflns_number_gt Values Differ by        39 Check
```

Alert level G

```
ABSMU01_ALERT_1_G  Calculation of _exptl_absorpt_correction_mu
                not performed for this radiation type.
PLAT004_ALERT_5_G Polymeric Structure Found with Maximum Dimension         3 Info
PLAT005_ALERT_5_G No Embedded Refinement Details Found  in the CIF     Please Do !
```

```
PLAT045_ALERT_1_G Calculated and Reported Z Differ by a Factor ...      0.500 Check
PLAT092_ALERT_4_G Check: Wavelength Given is not Cu,Ga,Mo,Ag,In Ka    0.29090 Ang.
PLAT199_ALERT_1_G Reported _cell_measurement_temperature ..... (K)        293 Check
PLAT200_ALERT_1_G Reported   _diffrn_ambient_temperature ..... (K)        293 Check
PLAT794_ALERT_5_G Tentative Bond Valency for Bi1       (III)     .       2.31 Info
PLAT794_ALERT_5_G Tentative Bond Valency for Bi2       (III)     .       2.26 Info
PLAT883_ALERT_1_G No Info/Value for _atom_sites_solution_primary .     Please Do !
PLAT910_ALERT_3_G Missing # of FCF Reflection(s) Below Theta(Min).          2 Note
PLAT912_ALERT_4_G Missing # of FCF Reflections Above STh/L=  0.600        452 Note
PLAT951_ALERT_5_G Calculated (ThMax) and CIF-Reported Kmax Differ           3 Units
PLAT952_ALERT_5_G Calculated (ThMax) and CIF-Reported Lmax Differ.          8 Units
PLAT957_ALERT_1_G Calculated (ThMax) and Actual (FCF) Kmax Differ           3 Units
PLAT958_ALERT_1_G Calculated (ThMax) and Actual (FCF) Lmax Differ.          8 Units
PLAT984_ALERT_1_G The Bi-f'=   -0.8723 Deviates from the B&C-Value    -0.8028 Check
PLAT985_ALERT_1_G The Bi-f"=    2.4794 Deviates from the B&C-Value     2.4568 Check
```

```
   1 ALERT level A = Most likely a serious problem - resolve or explain
   4 ALERT level B = A potentially serious problem, consider carefully
  61 ALERT level C = Check. Ensure it is not caused by an omission or oversight
  18 ALERT level G = General information/check it is not something unexpected

  17 ALERT type 1 CIF construction/syntax error, inconsistent or missing data
  53 ALERT type 2 Indicator that the structure model may be wrong or deficient
   5 ALERT type 3 Indicator that the structure quality may be low
   2 ALERT type 4 Improvement, methodology, query or suggestion
   7 ALERT type 5 Informative message, check
```

It is advisable to attempt to resolve as many as possible of the alerts in all categories. Often the minor alerts point to easily fixed oversights, errors and omissions in your CIF or refinement strategy, so attention to these fine details can be worthwhile. In order to resolve some of the more serious problems it may be necessary to carry out additional measurements or structure refinements. However, the purpose of your study may justify the reported deviations and the more serious of these should normally be commented upon in the discussion or experimental section of a paper or in the "special_details" fields of the CIF. checkCIF was carefully designed to identify outliers and unusual parameters, but every test has its limitations and alerts that are not important in a particular case may appear. Conversely, the absence of alerts does not guarantee there are no aspects of the results needing attention. It is up to the individual to critically assess their own results and, if necessary, seek expert advice.

Publication of your CIF in IUCr journals

A basic structural check has been run on your CIF. These basic checks will be run on all CIFs submitted for publication in IUCr journals (*Acta Crystallographica*, *Journal of Applied Crystallography*, *Journal of Synchrotron Radiation*); however, if you intend to submit to *Acta Crystallographica Section C* or *E* or *IUCrData*, you should make sure that full publication checks are run on the final version of your CIF prior to submission.

Publication of your CIF in other journals

Please refer to the *Notes for Authors* of the relevant journal for any special instructions relating to CIF submission.

PLATON version of 10/05/2023; check.def file version of 10/05/2023

Datablock I - ellipsoid plot

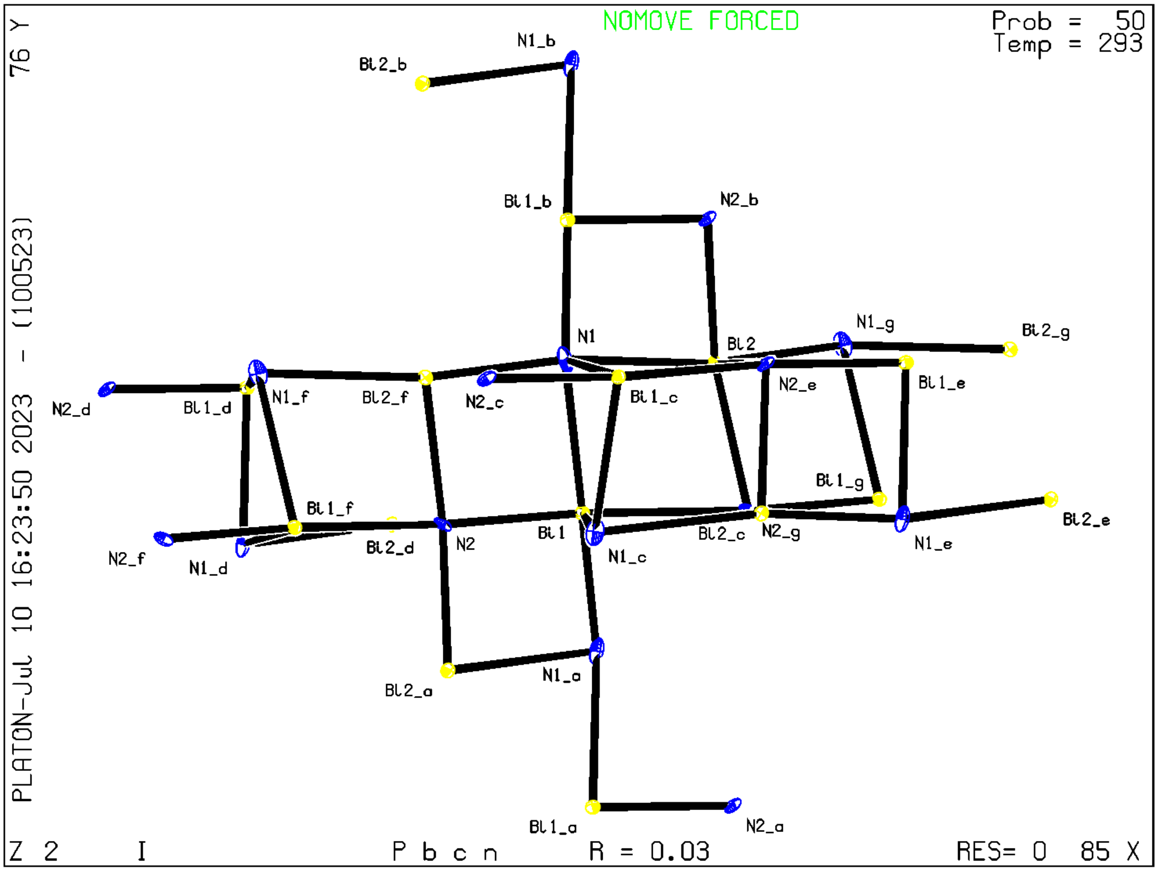

Supplement: Supplementary file 10 [file DataSheet8.PDF]
